# A Universal Trend of Reduced mRNA Stability near the Translation-Initiation Site in Prokaryotes and Eukaryotes

**DOI:** 10.1371/journal.pcbi.1000664

**Published:** 2010-02-05

**Authors:** Wanjun Gu, Tong Zhou, Claus O. Wilke

**Affiliations:** 1 Key Laboratory of Child Development and Learning Science of Ministry of Education of China, Southeast University, Nanjing, Jiangsu, China; 2 Center for Computational Biology and Bioinformatics, The University of Texas at Austin, Austin, Texas, United States of America; 3 Section of Integrative Biology, The University of Texas at Austin, Austin, Texas, United States of America; 4 Institute for Cell and Molecular Biology, The University of Texas at Austin, Austin, Texas, United States of America; Utrecht University, Netherlands

## Abstract

Recent studies have suggested that the thermodynamic stability of mRNA secondary structure near the start codon can regulate translation efficiency in *Escherichia coli*, and that translation is more efficient the less stable the secondary structure. We survey the complete genomes of 340 species for signals of reduced mRNA secondary structure near the start codon. Our analysis includes bacteria, archaea, fungi, plants, insects, fishes, birds, and mammals. We find that nearly all species show evidence for reduced mRNA stability near the start codon. The reduction in stability generally increases with increasing genomic GC content. In prokaryotes, the reduction also increases with decreasing optimal growth temperature. Within genomes, there is variation in the stability among genes, and this variation correlates with gene GC content, codon bias, and gene expression level. For birds and mammals, however, we do not find a genome-wide trend of reduced mRNA stability near the start codon. Yet the most GC rich genes in these organisms do show such a signal. We conclude that reduced stability of the mRNA secondary structure near the start codon is a universal feature of all cellular life. We suggest that the origin of this reduction is selection for efficient recognition of the start codon by initiator-tRNA.

## Author Summary

Synonymous mutations are mutations that change the nucleotide sequence of a gene without changing the amino-acid sequence. Because these mutations don't alter the expressed protein, they are frequently also called silent mutations. Yet increasing evidence demonstrates that synonymous mutations are not that silent. In particular, experimental work in *Escherichia coli* has shown that the choice of synonymous codons near the start codon can greatly influence protein production. Codons that allow the mRNA to fold into a stable secondary structure seem to inhibit efficient translation initiation. This observation suggests that selection should prefer reduced mRNA stability near the start codon in many organisms. Here, we show that this prediction generally holds true in most organisms, including bacteria, archaea, fungi, plants, insects, and fishes. In birds and mammals it doesn't hold true genome-wide, but it does hold true in the most GC-rich genes. In all organisms, the extent to which mRNA stability is reduced increases with increasing GC content. In prokaryotes, it also increases with decreasing optimal growing temperature. Thus, it seems that all organisms have to optimize their synonymous sites near the start codon to guarantee efficient protein translation.

## Introduction

Synonymous mutations are frequently used as a neutral baseline to detect selection pressures at the amino-acid level [1]. Yet many mechanisms are now known that cause selection pressure on synonymous sites. Translationally preferred codons are selected for accurate and efficient translation in bacteria, yeast, worm, fly, and even in mammals [2]–[13]. Selection on synonymous sites acts to increase the thermodynamic stability of DNA and RNA secondary structure [14]–[18], to improve splicing efficiency [19]–[21], and to assist protein co-translational folding [22]–[27].

Synonymous codon choice can also affect translation initiation. Most of the sequence elements that control translation initiation (e.g. the Shine-Dalgarno sequence in prokaryotes and the 5′ cap and Kozak consensus sequence in eukaryotes) are located in 5′ untranslated regions (UTRs) [28]–[30], where high conservation and AU-richness have been observed [31]–[34]. Yet Zalucki et al. found a significant bias towards usage of the AAA codon at the second amino acid position in *Escherichia coli* secretory proteins [35]. They proposed that selective pressure for high translation-initiation efficiency accounts for this codon usage bias. Other studies have demonstrated altered expression levels in *E. coli* after changing synonymous codons in the region downstream from the start codon [36]–[39]. Kudla et al. synthesized a library of 154 genes of green fluorescent protein (GFP) that had random changes at synonymous sites without any change in the amino-acid sequence [40]. They found that the GFP expression level varied 250-fold across the library. In this library, the stability of mRNA secondary structure near the start codon explained more than half of the variation in expression level: mRNAs with more stable local structure in this region had reduced protein expression [40]. These observations suggest that translation initiation is facilitated by a choice of synonymous codons that destabilize local mRNA secondary structure.

Here, we analyzed the local mRNA secondary structure at the 5′ end of the coding region in 340 species, including bacteria, archaea, fungi, plants, fishes, birds, and mammals. We used computational methods to predict the thermodynamic stability of local mRNA secondary structure in sliding windows downstream from the start codon, and used permutation tests to assess deviation from random expectation. We addressed the following questions: (i) Is there a selection pressure on synonymous sites to reduce the stability of local mRNA secondary structure at the translation-initiation region? (ii) Is such a selection pressure a general characteristic for all organisms? (iii) Does 5′ mRNA stability correlate with GC composition, codon usage bias, or gene expression level? (iv) In prokaryotes, does 5′ mRNA stability vary with the optimal growth temperature of the organism?

## Results

### mRNA stability is reduced near the translation-initiation region

We calculated the local folding energy (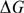) along the mRNA sequence using a sliding window of 30 nucleotides (nt) in length, moving from the start codon to the 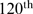 downstream nucleotide in steps of 10 nt (for a total of 13 windows). To quantify the deviation from expectation given a gene's amino-acid sequence and codon usage bias, we also calculated 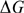 for 1000 permuted mRNA sequences. We obtained permuted sequences by randomly reshuffling synonymous codons within each gene. We then calculated a 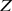-score, 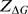, by comparing the 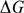 of the real mRNA segment to the distribution of 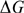 values of the permuted sequences (see Materials and Methods). 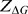 measures the extent to which local mRNA stability deviates from expectation. A positive 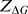 means that local mRNA stability is reduced, and a negative 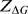 means that it is increased. For each window, we calculated a genome-wide mean 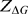 by averaging the corresponding 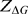 values over all genes in a genome.

**Figure 1:**
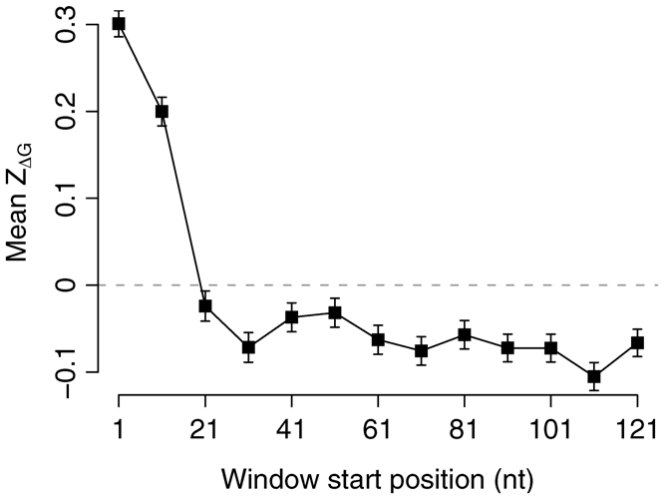
The mean and standard error of 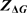 of each sliding window in *E. coli*.

We performed the sliding window analysis in 340 species, which included 276 bacteria, 35 archaea, 11 fungi, 2 plants, 2 insects, 4 fishes, 2 birds, and 8 mammals. Figure 1 shows an example of the mean 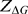 for 13 windows in *E. coli*. We observed a significant positive deviation of 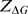 from zero in the first two windows (t-test: 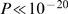 in both cases). The positive values of 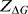 suggest selection for reduced mRNA stability at the 5′ end of the coding region. The 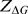 values further downstream decrease quickly and we observe negative 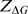 values in most downstream windows.

**Figure 2:**
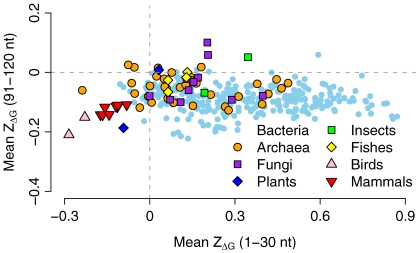
The mean 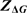 of the tenth window vs. the mean 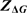 of the first window (5′ 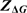). Each data point represents the entire genome of one organism.

Most species we studied showed a similar pattern to the one we observed in *E. coli* (Figure S1 and Table S1), except for plants and warm-blooded animals (birds and mammals). There was a clear increase in mean 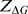 for windows close to the start codon. Because the 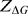 at the very start of the coding sequence generally showed the strongest signal of reduced mRNA stability, we will focus on this value for the remainder of this study. In the following, we refer to the 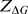 at the very start of the coding sequence also as the 5′ 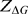. In prokaryotes, 262 out of 276 bacteria and 28 out of 35 archaea showed a positive 5′ 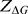 (Figure 2). In eukaryotes, 10 out of 11 fungi, 1 out of 2 plant, both insect species, and all four fish species we analyzed showed this pattern as well. All warm-blooded animals showed a negative 5′ 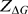 throughout the coding sequence. We list the mean and standard error of 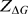 for all species and all windows in Table S1.

To investigate whether window size affected our results, we redid our analysis for four species (two bacteria, one archaeon, one fungus) using sliding windows of 20 nt and 40 nt, respectively. Results for these two window sizes were comparable to those obtained with a window size of 30 nt (Figures S2 and S3). For the same four species, we also recalculated 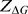 controlling for dinucleotide content, by using the DicodonShuffle algorithm [41]. The results were virtually unchanged compared to our standard shuffling method. (Figure S4).

### Genomic GC composition explains the major variation in 5′ 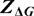


We found substantial variation in the mean 5′ 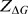 among different species (Figure 2). Therefore, we next aimed to identify the determinants of 5′ 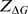 in different genomes. We first considered genomic nucleotide content.

**Figure 3:**
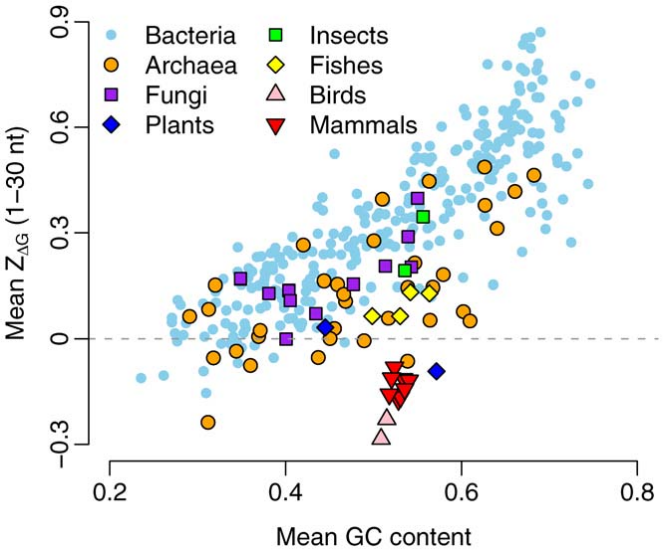
The mean 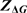 of the first window as a function of the genomic GC content. Each data point represents one organism.

We compared the mean 5′ 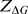 in each genome to the genome's GC content in coding sequences. We observed a strong positive correlation between the mean 5′ 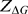 and the genomic GC content (Spearman's 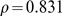, 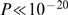) when plants and warm-blooded animals were excluded (Figure 3). Genomes with higher GC content had comparatively less stable mRNA secondary structure at the translation-initiation region. Since the thermodynamic stability of RNA secondary structure tends to be correlated to the RNA's GC content, we also looked into local deviations in a gene's GC content. We calculated 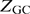, which measures the deviation in GC content in a 30 nt window relative to the average in the gene (see Materials and Methods). We found a negative correlation between genomic GC content and the mean 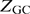 of the first window (Spearman's 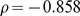, 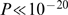). Thus, in GC-rich genomes, the sequence regions immediately downstream of the start codon were particularly GC poor (Figure S5).

Because mRNA stability was reduced only near the translation-initiation region, we expected that similarly GC content was reduced only near the start codon. Therefore, the correlation between genomic GC content and mean 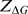 should decrease for windows further downstream. We found that indeed the correlation declined continuously and reached approximately zero at the 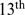 window (Figure S6).

Besides the statistical measure 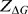, we also considered 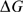 directly (Table S2). We found that the mean 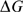 value of the first window (5′ 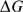) varied greatly among different species and was largely determined by genomic GC content (Spearman's 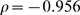, 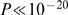, Figure S7). As expected, mRNA stability increased with increasing genomic GC content. In fact, we found similar relationships for windows further downstream, but the stability at the 5′ end of the mRNA was generally lower than the stability further downstream (Figure S8). Moreover, the difference between the mean 5′ 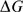 and the mean 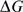 of the downstream windows increased with increasing genomic GC content. As an example, Figure S9 shows the relationship between the mean GC content and the difference in mean 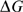 between the first and tenth window (Spearman correlation 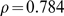, 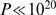, excluding birds and mammals). In summary, the results for 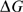 generally mirrored the ones for 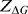.

### Optimal growth temperature affects 5′ 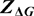 in prokaryotes

**Figure 4:**
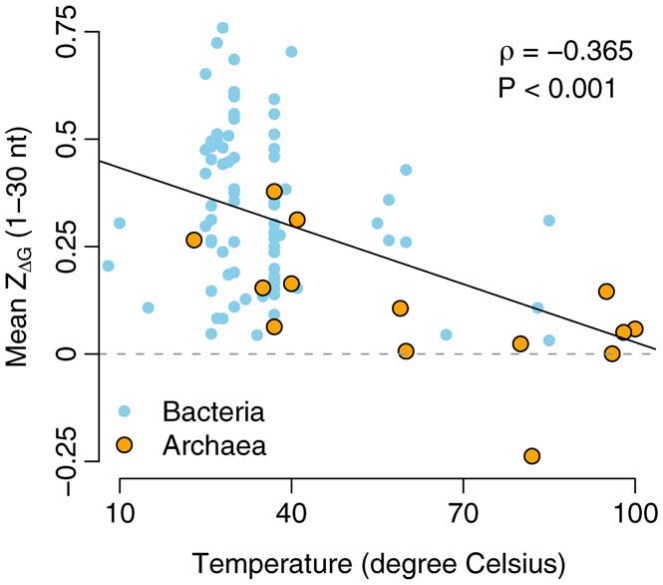
The mean 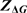 of the first window as a function of the optimal growth temperature in prokaryotes. Each data point represents one organism.

For prokaryotes, we analyzed whether the 5′ 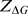 correlated with the optimal growth temperature (Figure 4). We found a significant negative correlation (Spearman's 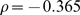, 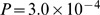). Similarly, the difference in 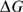 between the first and the tenth window declined significantly with temperature (Spearman's 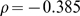, 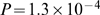). Thus, prokaryotes living in colder environments tended to have comparatively less stable mRNA secondary structure at the translation-initiation region. We found no correlations between temperature and either genomic GC content (Spearman's 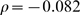, 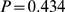) or the 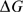 in the first window (Spearman's 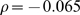, 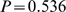). The lack of a correlation between temperature and genomic GC content agrees with the results of Ref. [42].

### Determinants of 5′ 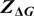 within genomes

In the previous subsections, we considered the mean 5′ 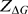 over all genes in a genome. But we expected that there should also be variation in mRNA stability among genes within one genome. Therefore, we next investigated the potential within-genome factors that may affect mRNA stability near the start codon.

**Figure 5:**
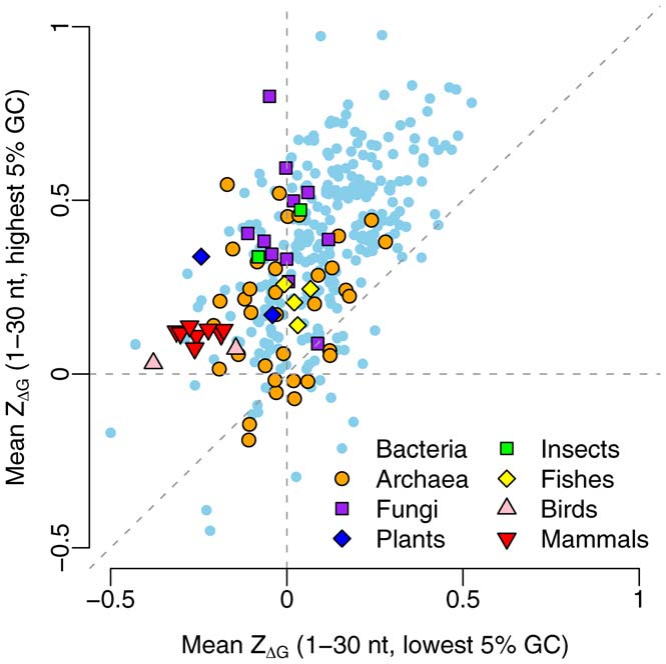
Comparison of the mean 5′ 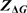 between genes with the highest 5% and the lowest 5% GC content within each genome. Each data point represents one organism.

We first considered gene GC content. We compared the mean 5′ 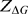 between genes with the highest 5% and the lowest 5% GC content in each species. In almost all genomes, including birds and mammals, the mean 5′ 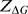 in GC-rich genes was higher than it was in GC-poor genes (Figure 5 and Table S3). The differences became weaker as we considered windows further downstream (Figure S10). Interestingly, even though the whole-genome mean 5′ 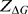 was negative in birds and mammals, GC-rich genes in these animals showed a positive 5′ 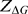 (Figure 5).

Next we considered codon usage bias. We used the effective number of codons (*ENC*) to measure the codon usage bias of each gene [43]. Lower *ENC* values indicate stronger bias. By comparing the bottom 5% of genes with the lowest *ENC* to the top 5% of genes with the highest *ENC*, we found that, in most species, genes with stronger codon bias had higher 5′ 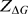 (Figure S11 and Table S3).

**Figure 6:**
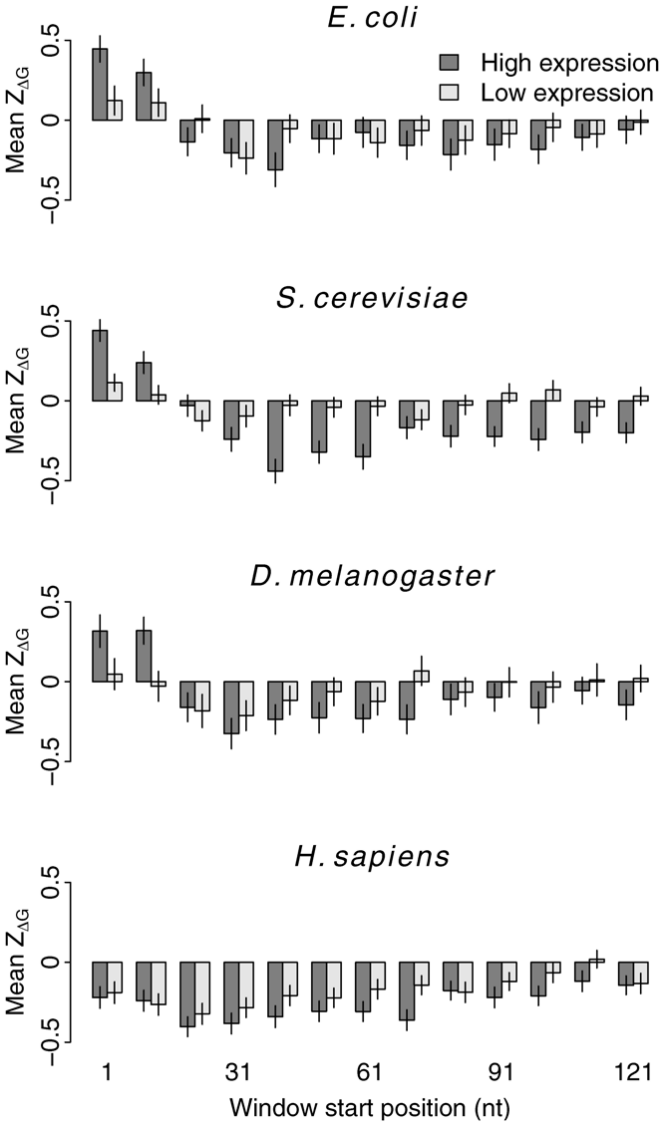
Comparison of the mean 5′ 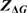 between genes with the highest 5% and the lowest 5% expression level in *E. coli*, *S. cerevisiae*, *D. melanogaster*, and *H. sapiens*.

Finally, we tested whether the reduction in 5′ mRNA stability increased with gene expression level. We compared the mean 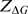 between genes with the highest 5% and the lowest 5% expression level in *E. coli*, *Drosophila melanogaster*, *Saccharomyces cerevisiae*, and *Homo sapiens*. In all species except *H. sapiens*, the mean 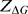 for the highest-expressed genes tended to be higher than that for the genes with the lowest expression level (Figure 6).

Since GC content, codon bias, and expression level all correlated with 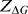, we tried to determine whether these quantities are independent sources of variation. We carried out a principle component regression [44] and found that GC content, *ENC*, and gene expression level contributed nearly equal to variation in 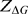 in *E. coli*, *S. cerevisiae*, and *D. melanogaster* (Figure S12). In human, GC content and *ENC*, but not gene expression level, contributed to the variation.

## Discussion

We have completed a broad survey of mRNA stability near the translation-initiation region of protein-coding genes. We have considered the complete genomes of 340 species, including bacteria, archaea, fungi, plants, insects, and vertebrates. We have found a general tendency for reduced mRNA stability in the first 30–40 nt of the coding sequence. In this region, mRNA stability tends to be less than expected given a gene's amino-acid sequence and codon-usage bias. Experimental work had previously suggested that increased local mRNA stability at the translation-initiation region could prevent efficient translation initiation and hence decrease gene expression level [38],[40].

We have found that there is variation in the extent to which mRNA stability is reduced both among and within genomes. Among genomes, GC content of coding sequences is a major predictor of the reduction in mRNA stability. The higher the GC content, the larger the reduction in mRNA stability at the 5′ end of the coding sequence (i.e., the larger 5′ 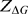). For prokaryotes, the optimal growth temperature also predicts 5′ 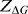. The lower the optimal growth temperature, the larger the reduction in mRNA stability. Within genomes, 5′ 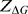 also increases with increasing GC content. In addition, it increases with increasing codon usage bias and gene expression level.

The region with reduced mRNA stability is located right downstream from the start codon and has a length of 30 to 40 nt (the first two windows in our analysis). This region is similar to the one identified by Kudla et al. [40]. Kudla et al. studied primarily a library of sequences encoding green fluorescent protein, but they also carried out a computational analysis of mRNA stability across the *E. coli* genome. They found that across the genome, 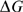 was significantly more positive (indicating reduced stability) in the region from nt 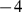 to 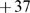 than immediately downstream [40]. Our work shows that Kudla et al.'s observation applies to most organisms with known genomes, including bacteria, archaea, and both single- and multi-celled eukaryotes. Further, by focusing on 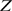 scores relative to the expectation in permuted sequences, our analysis excludes biases such as amino-acid content or preferred-codon usage as the cause of this signal.

Past the first two windows, 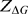 decayed quickly towards a negative asymptotic value. Thus, mRNA stability near the start codon is less than expected, but elsewhere in the gene it is generally higher than expected. The latter result is comparable to observations made by Chamary and Hurst [16] in the mouse genome and by Seffens and Digby [15] in individual genes from several species. Interestingly, for many organisms, 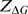 in windows 3 to 5 dips below the negative asymptotic value further downstream (Figure S1 and Table S1). This behavior seems to reflect a selection pressure for particularly stable local mRNA structure right after the translation-initiation region. This increased stability may compensate for the reduced mRNA stability in the translation-initiation region.

Previous works have identified AT-biased translation enhancers in prokaryotes [37], [45]–[47] and preferred nucleotide sequences regulating translation in eukaryotes [48] within the first 30 to 40 nt of the coding sequence. The mechanism by which these sequence motifs work is not currently known. We suggest that the primary mechanism may be destabilization of the mRNA structure near the start codon. By contrast, some motifs work by known mechanisms unrelated to RNA secondary structure. For example, alanine is preferred at the second amino-acid position in highly expressed proteins in several organisms [49] and its codon might bind to a complementary sequence in the 18S ribosomal RNA [50].

We found that the higher the GC content of a genome, the more was mRNA stability reduced at the translation-initiation region. This finding makes thermodynamic sense. GC-rich RNAs tend to fold into more stable structures than AU-rich RNAs, simply because a GC pair has three hydrogen bonds whereas an AU pair has only two. Thus, assuming that selection targets the same low 5′ mRNA stability in all organisms, we would expect that the decrease in stability is larger in GC-rich RNAs, simply because they start from a more-stable baseline. Whether selection actually targets the same low 5′ mRNA stability cannot be determined by our analysis. We found that the mean 5′ 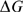 increased with increasing GC content. This increase could imply either that organisms with higher GC content can tolerate a higher 5′ mRNA stability or that the selection pressure to reduce 5′ mRNA stability in those organisms is counterbalanced by other selective forces or mutation pressures that increase GC content.

For prokaryotes, we addressed the question whether the optimal growth temperature affects 5′ 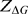. Thermodynamics predict that the lower the temperature at which an organism grows, the stronger should mRNA stability interfere with translation initiation. In agreement with this prediction, we found that the optimal growth temperature correlated negatively with 5′ 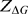. The organisms growing at the lowest temperatures showed the biggest reduction in mRNA stability at the beginning of the coding sequence. This result was independent of the relationship between 5′ 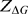 and genomic GC content. In our data set, the optimal growth temperature was not correlated with GC content. Even though some authors have argued that GC content correlates with temperature [51],[52], more recent studies have disputed this finding [42],[53]. Our results agree with these more recent studies.

Within individual genomes, the reduction of mRNA stability at the translation-initiation region was greater in GC-rich genes than in GC-poor ones. Besides GC content, we found that codon usage bias and gene expression level correlated with 5′ 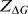. Because codon usage bias is correlated with gene expression level, in particular in fast-growing microbes [2],[3],[8],[11], these two correlations likely reflect the same underlying effect. The correlation with expression level mirrors the general observation that evolutionary constraints tend to increase with gene expression level [8], [11], [13], [54]–[58]. Whether expression level, codon usage bias, and GC content contribute independently to 5′ 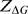 is unclear. These three quantities tend to all be correlated with each other, and we cannot easily disentangle which of these quantities is most important for reduced 5′ 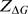. For example, in mammals, high GC content in genes can increase mRNA levels through increased efficiency of transcription or mRNA processing [59]. Using principal component regression, we showed that in *E. coli*, yeast, and fly, the three quantities codon usage bias, GC content, and gene expression level all contribute equally to reduced 5′ 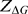, whereas in humans only GC content and codon-usage bias seem to contribute.

We found reduced mRNA stability near the start codon in a wide range of organisms, including both prokaryotes and eukaryotes. Yet warm-blooded animals (birds and mammals) showed no such trend on the whole-genome level, even though their genomic GC content is well within the range in which we found reduced mRNA stability in bacteria, archaea, fungi, insects, and fishes. We believe that our finding for birds and mammals was caused by the isochore structure of their genomes [60]. Gene GC content in these organisms ranges from 20% to 95% and is much more varied than in organisms without isochores. The whole-genome average of 5′ 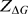 may not be meaningful in organisms with isochores. When we considered only to top 5% most GC-rich genes, we did find a moderate signal of reduced mRNA stability in these organisms as well.

What is the biological mechanism that links mRNA stability near the start codon to efficient protein translation? There are two possibilities. First, strong local mRNA secondary structure could interfere with ribosome binding. Second, it could interfere with start-codon recognition. We believe that the currently available evidence favors the latter explanation. In prokaryotes, ribosome binding occurs at the Shine-Dalgarno sequence, located a few nucleotides upstream from the start codon [28]. Kudla *et al.* [40] showed that synonymous mutations near the start codon can regulate protein expression. They concluded from computational modeling that the primary determinant of protein expression was the stability of local mRNA secondary structure near the start codon, not occlusion of the Shine-Dalgarno sequence by RNA secondary structure [40]. In eukaryotes, translation initiation follows a scanning mechanism. The 40S ribosomal subunit enters at the 5′ end of the mRNA and migrates linearly until it encounters the first AUG codon [30]. If synonymous mutations near the start codon could affect ribosome entry at the 5′ cap, there should be a correlation between 5′ UTR length and mRNA stability near the start codon. The further away the start codon is from the 5′ cap, the less should local mRNA stability near the start codon affect ribosome entry. However, we did not find such a relationship, neither within genomes nor among genomes (data not shown). Therefore, we suggest that both in prokaryotes and in eukaryotes, reduced mRNA stability at the translation-initiation region primarily facilitates efficient start-codon recognition.

## Materials and Methods

### Genomic data

We collected the genomes for 276 bacteria, 35 archaea, 11 fungi, 2 plants, 2 insects, 4 fishes, 2 birds, and 8 mammals. The genomic sequences of the bacteria, archaea, fungi, plants, and insects were downloaded from the NCBI FTP server (ftp://ftp.ncbi.nih.gov/), while the sequences of the vertebrates were obtained from Ensembl (http://www.ensembl.org/). We only considered coding sequences longer than 50 codons.

### Expression data

We collected previously published expression data for four species: for *E. coli*, we obtained gene expression levels measured in mRNAs per cell from Ref. [61]; for *S. cerevisiae*, we used expression data from Ref. [62]; for *D. melanogaster*, we used as expression level the geometric mean of expression data from different tissues obtained in Ref. [63]; and for *H. sapiens*, we also measured expression level as the geometric mean of expression among different tissues [64].

### Optimal growth temperature

We obtained optimal growth temperature data for 80 bacteria and 14 archaea from Ref. [42], which is a collection from multiple sources, including original publications, American Type Culture Collection, German Collection of Microorganisms and Cell Cultures, and Prokaryotic Growth Temperature Database.

### RNA secondary structure folding

We calculated RNA folding energies using the RNAfold program in the Vienna package [65],[66]. We used default settings: folding occurred at 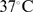; GU pairs were allowed; unpaired bases could participate in at most one dangling end; energy parameters were as reported in Ref. [67]. We evaluated only the minimum-free-energy structure. 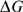 is the change in free energy from the unfolded state to this structure.

### mRNA randomization

If synonymous selection acts on mRNA folding near the start codon, then on average the secondary structure in this region should be less stable for the naturally occurring sequence than for permuted sequences. For each gene, we randomly reshuffled synonymous codons among sites with identical amino acids, to control for amino-acid sequence, codon usage bias, and GC content. We repreated this process 1000 times to obtain 1000 permuted sequences for each gene. For the wild-type sequence and each permuted sequence, we then calculated local mRNA folding energies in a sliding window of 30 nt (20 nt and 40 nt were also used in some species). To determine the deviation of the wild-type sequence from the permuted ones, we calculated the Z-score of the local mRNA stability (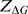) for each sliding window by:
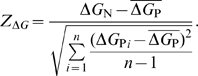
(1)


Here, 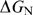 is the folding free energy for the naturally occurring sequence in the window under consideration, 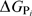 is the folding energy of the corresponding window of the 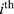 permuted sequence, and 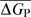 is the mean of 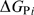 over all permuted sequences. The variable 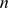 represents the total number of permuted sequences. Here, 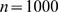.

Similarly, we evaluated the difference between the local mRNA GC composition of the wild-type sequence and the permuted sequences. The Z-score of local mRNA GC content (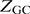) for each window can be expressed as:
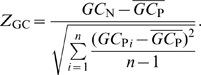
(2)


The definitions for 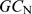, 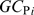, and 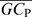 are analogous to 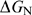, 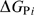, and 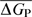 but refer to GC content rather than to free energy of folding.

## Supporting Information

Table S1 Mean and standard error of *Z_ΔG_* for each window.
(0.08 MB PDF)

Table S2 Mean and standard error of *ΔG* for each window.
(0.08 MB PDF)

Table S3 Intra-genome comparison on mean *Z_ΔG_* of the first window.
(0.06 MB PDF)

Figure S1 Distribution of the mean *Z_ΔG_* as a function of window start position in different groups of species.
(0.05 MB EPS)

Figure S2 The mean and standard error of *Z_ΔG_* of each sliding window (20 nt) in four species.
(0.03 MB EPS)

Figure S3 The mean and standard error of *Z_ΔG_* of each sliding window (40 nt) in four species.
(0.03 MB EPS)

Figure S4 Comparison of the mean *Z_ΔG_* obtained by different mRNA randomization procedures. Codon Shuffle algorithm permutes sequences by randomly reshuffling synonymous codons within each gene, which may change dinucleotide composition of the sequence; while DicodonShuffle algorithm shuffle codons preserving the dinucleotide composition.
(0.05 MB EPS)

Figure S5 The mean *Z*_GC_ of the first window as a function of the genomic GC content. Each data point represents one organism.
(0.14 MB EPS)

Figure S6 Spearman correlation coefficient ρ between the mean *Z_ΔG_* and the genomic GC content for each window in bacteria, archaea, and fungi.
(0.02 MB EPS)

Figure S7 The mean *ΔG* of the first window as a function of the genomic GC content. Each data point represents one organism.
(0.14 MB EPS)

Figure S8 The mean *ΔG* of the tenth window vs. the mean *ΔG* of the first window. Each data point represents the entire genome of one organism.
(0.14 MB EPS)

Figure S9 The difference in mean *ΔG* between the first and the tenth window as a function of the genomic GC content. Each data point represents one organism.
(0.14 MB EPS)

Figure S10 Distribution of the difference in mean *Z_ΔG_* between genes with the highest 5% and the lowest 5% GC content for the entire data set of all 340 species.
(0.11 MB EPS)

Figure S11 Comparison of the mean 5′ *Z_ΔG_* between genes with the highest 5% and the lowest 5% *ENC* within each genome. Each data point represents one organism.
(0.14 MB EPS)

Figure S12 Principal component regression of 5′ *Z_ΔG_* against GC content, expression level, and *ENC* value. PC1, PC2, and PC3 denote the first, second, and third principal component.
(0.02 MB EPS)
