# Supplementary material for: A Universal Trend of Reduced mRNA Stability near the Translation-Initiation Site in Prokaryotes and Eukaryotes
Source: PLoS Comput Biol. 2010 Feb 5;6(2):e1000664. doi: 10.1371/journal.pcbi.1000664 (PMC2816680; doi:10.1371/journal.pcbi.1000664)
Supplement: Table S1 — Mean and standard error of ZΔG for each window. (0.08 MB PDF) [file pcbi.1000664.s001.pdf]

Table S1: Mean and standard error of $Z_{\Delta G}$ for each window

Type	Species	Window 1	Window 2	Window 3	Window 4	Window 5	Window 6	Window 7	Window 8	Window 9	Window 10	Window 11	Window 12	Window 13
Bacteria	*Acaryochloris marina*	0.22 ± 0.01	0.20 ± 0.01	0.03 ± 0.01	-0.11 ± 0.01	-0.10 ± 0.01	-0.08 ± 0.01	-0.08 ± 0.01	-0.06 ± 0.01	-0.05 ± 0.01	-0.08 ± 0.01	-0.08 ± 0.01	-0.08 ± 0.01	-0.08 ± 0.01
Bacteria	*Acholeplasma laidlawii*	0.13 ± 0.02	-0.04 ± 0.03	-0.23 ± 0.03	-0.24 ± 0.03	-0.22 ± 0.03	-0.29 ± 0.03	-0.27 ± 0.03	-0.22 ± 0.03	-0.22 ± 0.03	-0.22 ± 0.03	-0.17 ± 0.03	-0.20 ± 0.03	-0.20 ± 0.03
Bacteria	*Acidiphilium cryptum*	0.54 ± 0.02	0.43 ± 0.02	0.18 ± 0.02	0.04 ± 0.02	-0.01 ± 0.02	-0.02 ± 0.02	-0.06 ± 0.02	-0.07 ± 0.02	-0.07 ± 0.02	-0.07 ± 0.02	-0.06 ± 0.02	-0.06 ± 0.02	-0.08 ± 0.02
Bacteria	*Acidithiobacillus ferrooxidans*	0.36 ± 0.02	0.26 ± 0.02	0.08 ± 0.02	-0.07 ± 0.02	-0.08 ± 0.02	-0.11 ± 0.02	-0.08 ± 0.02	-0.08 ± 0.02	-0.12 ± 0.02	-0.10 ± 0.02	-0.08 ± 0.02	-0.08 ± 0.02	-0.06 ± 0.02
Bacteria	*Acidobacteria bacterium*	0.42 ± 0.01	0.39 ± 0.02	0.21 ± 0.02	0.06 ± 0.02	-0.01 ± 0.02	-0.01 ± 0.02	-0.04 ± 0.02	-0.05 ± 0.02	-0.03 ± 0.02	-0.08 ± 0.02	-0.08 ± 0.02	-0.07 ± 0.02	-0.07 ± 0.01
Bacteria	*Acidobacterium capsulatum*	0.50 ± 0.02	0.46 ± 0.02	0.23 ± 0.02	0.04 ± 0.02	0.02 ± 0.02	-0.02 ± 0.02	-0.04 ± 0.02	-0.07 ± 0.02	-0.06 ± 0.02	-0.09 ± 0.02	-0.08 ± 0.02	-0.10 ± 0.02	-0.11 ± 0.02
Bacteria	*Acidothermus cellulolyticus*	0.25 ± 0.02	0.21 ± 0.02	0.02 ± 0.02	-0.04 ± 0.02	-0.04 ± 0.02	-0.05 ± 0.02	-0.08 ± 0.02	-0.07 ± 0.02	-0.08 ± 0.02	-0.05 ± 0.02	-0.07 ± 0.02	-0.11 ± 0.02	-0.11 ± 0.02
Bacteria	*Acidovorax citrulli*	0.78 ± 0.02	0.61 ± 0.02	0.27 ± 0.02	0.13 ± 0.02	0.04 ± 0.02	0.00 ± 0.02	-0.02 ± 0.02	-0.07 ± 0.02	-0.08 ± 0.02	-0.11 ± 0.02	-0.13 ± 0.02	-0.12 ± 0.02	-0.14 ± 0.01
Bacteria	*Acinetobacter baumannii*	0.09 ± 0.02	0.01 ± 0.02	-0.14 ± 0.02	-0.19 ± 0.02	-0.20 ± 0.02	-0.15 ± 0.02	-0.12 ± 0.02	-0.13 ± 0.02	-0.13 ± 0.02	-0.09 ± 0.02	-0.10 ± 0.02	-0.10 ± 0.02	-0.12 ± 0.02
Bacteria	*Actinobacillus pleuropneumoniae*	0.24 ± 0.02	0.11 ± 0.02	-0.10 ± 0.02	-0.11 ± 0.03	-0.11 ± 0.02	-0.12 ± 0.02	-0.10 ± 0.02	-0.13 ± 0.02	-0.14 ± 0.02	-0.10 ± 0.02	-0.11 ± 0.02	-0.14 ± 0.02	-0.14 ± 0.02
Bacteria	*Aeromonas hydrophila*	0.65 ± 0.02	0.47 ± 0.02	0.17 ± 0.02	0.11 ± 0.02	0.04 ± 0.02	0.04 ± 0.02	0.02 ± 0.02	0.01 ± 0.02	-0.03 ± 0.02	-0.05 ± 0.02	-0.07 ± 0.02	-0.08 ± 0.02	-0.08 ± 0.02
Bacteria	*Agrobacterium radiobacter*	0.55 ± 0.01	0.41 ± 0.01	0.13 ± 0.01	0.02 ± 0.01	-0.06 ± 0.01	-0.05 ± 0.01	-0.09 ± 0.01	-0.06 ± 0.01	-0.08 ± 0.01	-0.11 ± 0.01	-0.08 ± 0.01	-0.11 ± 0.01	-0.12 ± 0.01
Bacteria	*Akkermansia muciniphila*	0.50 ± 0.02	0.40 ± 0.02	0.06 ± 0.02	-0.11 ± 0.02	-0.13 ± 0.02	-0.13 ± 0.02	-0.12 ± 0.02	-0.12 ± 0.02	-0.10 ± 0.02	-0.13 ± 0.02	-0.12 ± 0.02	-0.13 ± 0.02	-0.09 ± 0.02
Bacteria	*Alcanivorax borkumensis*	0.34 ± 0.02	0.18 ± 0.02	-0.04 ± 0.02	-0.12 ± 0.02	-0.12 ± 0.02	-0.11 ± 0.02	-0.14 ± 0.02	-0.16 ± 0.02	-0.13 ± 0.02	-0.12 ± 0.02	-0.12 ± 0.02	-0.18 ± 0.02	-0.17 ± 0.02
Bacteria	*Aliivibrio salmonicida*	0.23 ± 0.02	0.06 ± 0.02	-0.16 ± 0.02	-0.14 ± 0.02	-0.30 ± 0.02	-0.29 ± 0.02	-0.21 ± 0.02	-0.09 ± 0.02	-0.15 ± 0.02	-0.19 ± 0.02	-0.15 ± 0.02	-0.18 ± 0.02	-0.17 ± 0.02
Bacteria	*Alkalilimnicola ehrlichii*	0.63 ± 0.02	0.48 ± 0.02	0.18 ± 0.02	-0.03 ± 0.02	-0.10 ± 0.02	-0.11 ± 0.02	-0.10 ± 0.02	-0.15 ± 0.02	-0.08 ± 0.02	-0.10 ± 0.02	-0.12 ± 0.02	-0.15 ± 0.02	-0.11 ± 0.02
Bacteria	*Alkaliphilus metalliredigens*	0.15 ± 0.01	0.11 ± 0.01	0.01 ± 0.02	-0.02 ± 0.02	-0.02 ± 0.02	-0.05 ± 0.02	-0.08 ± 0.02	-0.06 ± 0.02	-0.07 ± 0.02	-0.04 ± 0.02	-0.07 ± 0.02	-0.09 ± 0.02	-0.03 ± 0.02
Bacteria	*Alteromonas macleodii*	0.17 ± 0.02	0.11 ± 0.02	-0.10 ± 0.02	-0.10 ± 0.02	-0.16 ± 0.02	-0.18 ± 0.02	-0.12 ± 0.02	-0.13 ± 0.02	-0.12 ± 0.02	-0.10 ± 0.02	-0.13 ± 0.02	-0.12 ± 0.02	-0.14 ± 0.02
Bacteria	*Anabaena variabilis*	0.13 ± 0.01	0.11 ± 0.01	-0.02 ± 0.02	-0.11 ± 0.02	-0.18 ± 0.02	-0.15 ± 0.02	-0.10 ± 0.01	-0.09 ± 0.01	-0.08 ± 0.02	-0.07 ± 0.01	-0.07 ± 0.01	-0.07 ± 0.01	-0.08 ± 0.01
Bacteria	*Anaerocellum thermophilum*	0.04 ± 0.02	0.00 ± 0.02	-0.04 ± 0.02	-0.08 ± 0.02	-0.03 ± 0.02	-0.03 ± 0.02	-0.05 ± 0.02	-0.04 ± 0.02	-0.03 ± 0.02	-0.04 ± 0.02	-0.04 ± 0.02	-0.04 ± 0.02	-0.08 ± 0.02
Bacteria	*Anaeromyxobacter dehalogenans*	0.72 ± 0.02	0.63 ± 0.02	0.36 ± 0.02	0.14 ± 0.02	0.05 ± 0.02	-0.02 ± 0.02	-0.02 ± 0.02	-0.03 ± 0.02	-0.06 ± 0.02	-0.06 ± 0.02	-0.06 ± 0.02	-0.06 ± 0.02	-0.06 ± 0.02
Bacteria	*Anaplasma marginale*	0.15 ± 0.03	0.17 ± 0.03	0.02 ± 0.03	-0.06 ± 0.03	0.00 ± 0.03	-0.09 ± 0.03	-0.13 ± 0.03	-0.18 ± 0.03	-0.11 ± 0.03	-0.14 ± 0.03	-0.07 ± 0.03	-0.06 ± 0.03	-0.05 ± 0.03
Bacteria	*Anoxybacillus flavithermus*	0.03 ± 0.02	0.05 ± 0.02	-0.01 ± 0.02	0.02 ± 0.02	0.00 ± 0.02	-0.03 ± 0.02	-0.03 ± 0.02	-0.06 ± 0.02	-0.06 ± 0.02	-0.05 ± 0.02	-0.04 ± 0.02	-0.08 ± 0.02	-0.07 ± 0.02
Bacteria	*Aquifex aeolicus*	0.03 ± 0.03	0.01 ± 0.03	0.00 ± 0.03	-0.04 ± 0.03	-0.11 ± 0.03	-0.07 ± 0.03	0.00 ± 0.03	-0.03 ± 0.03	-0.04 ± 0.03	-0.07 ± 0.03	-0.07 ± 0.03	-0.02 ± 0.03	-0.05 ± 0.03
Bacteria	*Archaeoglobus fulgidus*	0.11 ± 0.02	0.11 ± 0.02	0.03 ± 0.02	0.03 ± 0.02	0.01 ± 0.02	0.02 ± 0.02	-0.02 ± 0.02	-0.02 ± 0.02	-0.02 ± 0.02	-0.02 ± 0.02	0.03 ± 0.02	0.00 ± 0.02	0.03 ± 0.02
Bacteria	*Arcobacter butzleri*	0.06 ± 0.02	0.00 ± 0.02	-0.18 ± 0.03	-0.19 ± 0.02	-0.19 ± 0.02	-0.21 ± 0.03	-0.18 ± 0.02	-0.13 ± 0.02	-0.09 ± 0.02	-0.11 ± 0.02	-0.09 ± 0.02	-0.07 ± 0.02	-0.07 ± 0.02
Bacteria	*Aromatoleum aromaticum*	0.51 ± 0.02	0.41 ± 0.02	0.16 ± 0.02	0.06 ± 0.02	0.02 ± 0.02	-0.01 ± 0.02	-0.03 ± 0.02	-0.04 ± 0.02	-0.04 ± 0.02	-0.06 ± 0.02	-0.07 ± 0.02	-0.08 ± 0.02	-0.11 ± 0.02
Bacteria	*Arthrobacter aurescens*	0.35 ± 0.02	0.27 ± 0.02	0.08 ± 0.02	0.01 ± 0.02	0.01 ± 0.02	-0.01 ± 0.02	-0.06 ± 0.02	-0.05 ± 0.02	-0.05 ± 0.02	-0.04 ± 0.02	-0.02 ± 0.02	-0.05 ± 0.02	-0.06 ± 0.02
Bacteria	*Aster yellows*	-0.01 ± 0.04	0.03 ± 0.04	-0.02 ± 0.04	-0.06 ± 0.04	-0.01 ± 0.04	-0.01 ± 0.04	-0.01 ± 0.04	0.03 ± 0.04	-0.05 ± 0.04	-0.03 ± 0.04	-0.04 ± 0.04	0.00 ± 0.04	-0.09 ± 0.04
Bacteria	*Azoarcus sp. BH72*	0.71 ± 0.02	0.56 ± 0.02	0.22 ± 0.02	0.09 ± 0.02	0.02 ± 0.02	0.02 ± 0.02	-0.05 ± 0.02	-0.02 ± 0.02	-0.05 ± 0.02	-0.09 ± 0.02	-0.08 ± 0.02	-0.09 ± 0.02	-0.07 ± 0.02
Bacteria	*Azorhizobium caulinodans*	0.53 ± 0.02	0.43 ± 0.02	0.25 ± 0.02	0.10 ± 0.02	0.06 ± 0.02	0.03 ± 0.02	0.05 ± 0.02	-0.02 ± 0.02	-0.01 ± 0.02	-0.04 ± 0.02	-0.04 ± 0.02	-0.05 ± 0.02	-0.05 ± 0.02
Bacteria	*Azotobacter vinelandii*	0.60 ± 0.02	0.48 ± 0.02	0.23 ± 0.02	0.10 ± 0.02	0.04 ± 0.02	0.01 ± 0.02	-0.03 ± 0.02	-0.06 ± 0.02	-0.07 ± 0.02	-0.08 ± 0.01	-0.07 ± 0.01	-0.07 ± 0.01	-0.07 ± 0.01
Bacteria	*Bacillus amyloliquefaciens*	0.29 ± 0.02	0.21 ± 0.02	0.07 ± 0.02	0.05 ± 0.02	0.00 ± 0.02	-0.01 ± 0.02	-0.06 ± 0.02	-0.07 ± 0.02	-0.06 ± 0.02	-0.07 ± 0.02	-0.07 ± 0.02	-0.08 ± 0.02	-0.09 ± 0.02
Bacteria	*Bacteroides fragilis*	0.37 ± 0.01	0.17 ± 0.02	-0.21 ± 0.02	-0.26 ± 0.02	-0.21 ± 0.02	-0.13 ± 0.02	-0.12 ± 0.02	-0.12 ± 0.02	-0.14 ± 0.02	-0.15 ± 0.02	-0.15 ± 0.02	-0.11 ± 0.02	-0.09 ± 0.02
Bacteria	*Bartonella bacilliformis*	0.04 ± 0.03	-0.01 ± 0.03	-0.15 ± 0.03	-0.13 ± 0.03	-0.16 ± 0.03	-0.22 ± 0.03	-0.17 ± 0.03	-0.13 ± 0.03	-0.10 ± 0.03	-0.11 ± 0.03	-0.09 ± 0.03	-0.15 ± 0.03	-0.13 ± 0.03
Bacteria	*Baumannia cicadellinicola*	-0.02 ± 0.04	-0.23 ± 0.05	-0.31 ± 0.05	-0.25 ± 0.05	-0.25 ± 0.05	-0.15 ± 0.05	-0.20 ± 0.05	-0.11 ± 0.04	-0.13 ± 0.05	-0.16 ± 0.04	-0.18 ± 0.05	-0.10 ± 0.04	-0.10 ± 0.04
Bacteria	*Bdellovibrio bacteriovorus*	0.36 ± 0.02	0.32 ± 0.02	0.27 ± 0.02	0.11 ± 0.02	-0.15 ± 0.02	-0.16 ± 0.02	-0.15 ± 0.02	-0.11 ± 0.02	-0.13 ± 0.02	-0.13 ± 0.02	-0.12 ± 0.02	-0.15 ± 0.02	-0.13 ± 0.02
Bacteria	*Beijerinckia indica*	0.34 ± 0.02	0.24 ± 0.02	0.00 ± 0.02	-0.08 ± 0.02	-0.10 ± 0.02	-0.09 ± 0.02	-0.08 ± 0.02	-0.10 ± 0.02	-0.08 ± 0.02	-0.10 ± 0.02	-0.11 ± 0.02	-0.13 ± 0.02	-0.12 ± 0.02

Continued on next page

Type	Species	Window 1	Window 2	Window 3	Window 4	Window 5	Window 6	Window 7	Window 8	Window 9	Window 10	Window 11	Window 12	Window 13
Bacteria	*Beutenbergia cavernae*	0.53 ± 0.02	0.38 ± 0.02	0.14 ± 0.02	0.09 ± 0.02	0.05 ± 0.02	0.01 ± 0.02	-0.02 ± 0.02	-0.02 ± 0.02	-0.05 ± 0.02	-0.06 ± 0.02	-0.06 ± 0.02	-0.03 ± 0.02	-0.08 ± 0.02
Bacteria	*Bifidobacterium adolescentis*	0.39 ± 0.03	0.30 ± 0.03	0.10 ± 0.03	0.02 ± 0.03	-0.01 ± 0.03	-0.07 ± 0.03	-0.08 ± 0.03	-0.08 ± 0.03	-0.09 ± 0.03	-0.14 ± 0.03	-0.05 ± 0.03	-0.05 ± 0.03	-0.06 ± 0.03
Bacteria	*Bordetella avium*	0.58 ± 0.02	0.35 ± 0.02	0.03 ± 0.02	-0.06 ± 0.02	-0.09 ± 0.02	-0.09 ± 0.02	-0.11 ± 0.02	-0.12 ± 0.02	-0.15 ± 0.02	-0.16 ± 0.02	-0.14 ± 0.02	-0.18 ± 0.02	-0.16 ± 0.02
Bacteria	*Borrelia afzelii*	0.01 ± 0.04	-0.03 ± 0.04	-0.05 ± 0.04	-0.14 ± 0.04	-0.17 ± 0.04	-0.13 ± 0.04	-0.07 ± 0.04	-0.05 ± 0.04	-0.07 ± 0.04	0.00 ± 0.03	-0.04 ± 0.04	-0.04 ± 0.03	-0.07 ± 0.04
Bacteria	*Brachyspira hyodysenteriae*	0.10 ± 0.02	0.08 ± 0.02	-0.04 ± 0.02	-0.06 ± 0.02	-0.10 ± 0.02	-0.08 ± 0.02	-0.06 ± 0.02	-0.05 ± 0.02	0.00 ± 0.02	-0.02 ± 0.02	-0.06 ± 0.02	-0.07 ± 0.02	-0.01 ± 0.02
Bacteria	*Bradyrhizobium japonicum*	0.50 ± 0.01	0.44 ± 0.01	0.27 ± 0.01	0.10 ± 0.01	0.04 ± 0.01	0.00 ± 0.01	-0.03 ± 0.01	-0.05 ± 0.01	-0.04 ± 0.01	-0.08 ± 0.01	-0.08 ± 0.01	-0.08 ± 0.01	-0.09 ± 0.01
Bacteria	*Brevibacillus brevis*	0.21 ± 0.01	0.15 ± 0.01	0.05 ± 0.01	0.01 ± 0.01	0.02 ± 0.01	0.02 ± 0.01	-0.01 ± 0.01	0.01 ± 0.01	-0.01 ± 0.01	0.00 ± 0.01	0.00 ± 0.01	-0.03 ± 0.01	-0.02 ± 0.01
Bacteria	*Brucella abortus*	0.44 ± 0.02	0.30 ± 0.02	0.12 ± 0.02	-0.04 ± 0.02	-0.13 ± 0.02	-0.13 ± 0.02	-0.08 ± 0.02	-0.10 ± 0.02	-0.10 ± 0.02	-0.10 ± 0.02	-0.12 ± 0.02	-0.16 ± 0.02	-0.17 ± 0.02
Bacteria	*Buchnera aphidicola*	0.02 ± 0.04	0.04 ± 0.04	-0.05 ± 0.04	-0.03 ± 0.04	-0.09 ± 0.04	-0.09 ± 0.04	-0.05 ± 0.04	-0.10 ± 0.04	-0.03 ± 0.04	-0.04 ± 0.04	-0.02 ± 0.04	-0.13 ± 0.05	-0.06 ± 0.04
Bacteria	*Burkholderia ambifaria*	0.84 ± 0.01	0.67 ± 0.01	0.29 ± 0.01	0.13 ± 0.01	0.06 ± 0.01	0.02 ± 0.01	-0.04 ± 0.01	-0.06 ± 0.01	-0.10 ± 0.01	-0.10 ± 0.01	-0.15 ± 0.01	-0.14 ± 0.01	-0.16 ± 0.01
Bacteria	*Caldicellulosiruptor saccharolyticus*	0.03 ± 0.02	0.03 ± 0.02	0.01 ± 0.02	-0.02 ± 0.02	-0.05 ± 0.02	-0.02 ± 0.02	-0.06 ± 0.02	-0.04 ± 0.02	-0.08 ± 0.02	-0.08 ± 0.02	-0.03 ± 0.02	-0.09 ± 0.02	-0.06 ± 0.02
Bacteria	*Campylobacter concisus*	0.21 ± 0.02	0.08 ± 0.02	-0.11 ± 0.03	-0.12 ± 0.03	-0.16 ± 0.03	-0.19 ± 0.02	-0.17 ± 0.03	-0.18 ± 0.02	-0.20 ± 0.02	-0.13 ± 0.02	-0.15 ± 0.03	-0.15 ± 0.02	-0.20 ± 0.02
Bacteria	*Candidatus Amoebophilus*	0.17 ± 0.03	0.07 ± 0.03	-0.04 ± 0.03	-0.13 ± 0.03	-0.16 ± 0.03	-0.13 ± 0.03	-0.08 ± 0.03	-0.07 ± 0.03	-0.09 ± 0.03	-0.01 ± 0.03	-0.04 ± 0.03	-0.05 ± 0.03	-0.07 ± 0.03
Bacteria	*Carboxydothermus hydrogenoformans*	0.04 ± 0.02	0.02 ± 0.02	-0.07 ± 0.02	-0.05 ± 0.02	-0.05 ± 0.02	-0.07 ± 0.02	-0.08 ± 0.02	-0.06 ± 0.02	-0.12 ± 0.02	-0.10 ± 0.02	-0.09 ± 0.02	-0.07 ± 0.02	-0.08 ± 0.02
Bacteria	*Caulobacter crescentus*	0.55 ± 0.02	0.45 ± 0.02	0.22 ± 0.02	0.05 ± 0.02	0.01 ± 0.02	0.01 ± 0.02	0.03 ± 0.02	0.00 ± 0.02	-0.05 ± 0.02	-0.05 ± 0.02	-0.07 ± 0.02	-0.09 ± 0.02	-0.07 ± 0.02
Bacteria	*Cellvibrio japonicus*	0.33 ± 0.02	0.27 ± 0.02	0.07 ± 0.02	-0.06 ± 0.02	-0.14 ± 0.02	-0.13 ± 0.02	-0.12 ± 0.02	-0.09 ± 0.02	-0.12 ± 0.02	-0.09 ± 0.02	-0.10 ± 0.02	-0.10 ± 0.02	-0.14 ± 0.02
Bacteria	*Chlamydia muridarum*	0.06 ± 0.03	-0.02 ± 0.03	-0.13 ± 0.04	-0.10 ± 0.03	-0.15 ± 0.04	-0.19 ± 0.04	-0.12 ± 0.04	-0.21 ± 0.04	-0.20 ± 0.04	-0.15 ± 0.04	-0.16 ± 0.04	-0.19 ± 0.04	-0.17 ± 0.04
Bacteria	*Chlamydophila abortus*	0.06 ± 0.03	0.02 ± 0.03	-0.16 ± 0.04	-0.16 ± 0.04	-0.18 ± 0.04	-0.19 ± 0.04	-0.11 ± 0.03	-0.11 ± 0.03	-0.10 ± 0.04	-0.09 ± 0.03	-0.05 ± 0.03	-0.11 ± 0.04	-0.12 ± 0.03
Bacteria	*Chlorobaculum parvum*	0.55 ± 0.02	0.37 ± 0.02	0.10 ± 0.02	-0.02 ± 0.02	-0.02 ± 0.02	-0.03 ± 0.02	-0.04 ± 0.02	-0.07 ± 0.02	-0.05 ± 0.02	-0.09 ± 0.02	-0.08 ± 0.02	-0.06 ± 0.02	-0.09 ± 0.02
Bacteria	*Chlorobium chlorochromatii*	0.24 ± 0.02	0.14 ± 0.02	-0.15 ± 0.03	-0.26 ± 0.03	-0.27 ± 0.03	-0.19 ± 0.02	-0.15 ± 0.02	-0.17 ± 0.02	-0.17 ± 0.02	-0.16 ± 0.02	-0.16 ± 0.02	-0.17 ± 0.02	-0.13 ± 0.02
Bacteria	*Chloroflexus aggregans*	0.32 ± 0.02	0.28 ± 0.02	0.10 ± 0.02	-0.01 ± 0.02	-0.06 ± 0.02	-0.07 ± 0.02	-0.06 ± 0.02	-0.04 ± 0.02	-0.03 ± 0.02	-0.08 ± 0.02	-0.07 ± 0.02	-0.04 ± 0.02	-0.04 ± 0.02
Bacteria	*Chloroherpeton thalassium*	0.30 ± 0.02	0.21 ± 0.02	-0.02 ± 0.02	-0.07 ± 0.02	-0.10 ± 0.02	-0.08 ± 0.02	-0.03 ± 0.02	-0.09 ± 0.02	-0.09 ± 0.02	-0.10 ± 0.02	-0.06 ± 0.02	-0.10 ± 0.02	-0.13 ± 0.02
Bacteria	*Chromobacterium violaceum*	0.61 ± 0.02	0.44 ± 0.02	0.17 ± 0.02	0.05 ± 0.02	0.03 ± 0.02	-0.03 ± 0.02	-0.04 ± 0.02	-0.06 ± 0.02	-0.09 ± 0.02	-0.11 ± 0.02	-0.13 ± 0.02	-0.11 ± 0.02	-0.11 ± 0.02
Bacteria	*Chromohalobacter salexigens*	0.56 ± 0.02	0.39 ± 0.02	0.11 ± 0.02	0.00 ± 0.02	-0.03 ± 0.02	-0.06 ± 0.02	-0.08 ± 0.02	-0.10 ± 0.02	-0.10 ± 0.02	-0.14 ± 0.02	-0.15 ± 0.02	-0.13 ± 0.02	-0.15 ± 0.02
Bacteria	*Citrobacter koseri*	0.34 ± 0.02	0.26 ± 0.02	0.05 ± 0.02	0.02 ± 0.02	-0.02 ± 0.02	0.00 ± 0.02	0.00 ± 0.02	-0.04 ± 0.02	-0.05 ± 0.02	-0.05 ± 0.02	-0.05 ± 0.02	-0.06 ± 0.02	-0.10 ± 0.02
Bacteria	*Clavibacter michiganensis*	0.75 ± 0.02	0.55 ± 0.02	0.26 ± 0.02	0.13 ± 0.02	0.10 ± 0.02	0.07 ± 0.02	0.02 ± 0.02	0.01 ± 0.02	0.00 ± 0.02	-0.01 ± 0.02	-0.02 ± 0.02	-0.02 ± 0.02	-0.03 ± 0.02
Bacteria	*Clostridium acetobutylicum*	0.09 ± 0.02	0.03 ± 0.02	-0.02 ± 0.02	-0.07 ± 0.02	-0.06 ± 0.02	-0.07 ± 0.02	-0.04 ± 0.02	-0.03 ± 0.02	-0.04 ± 0.02	-0.05 ± 0.02	-0.06 ± 0.02	-0.06 ± 0.02	-0.04 ± 0.02
Bacteria	*Colwellia psychrerythraea*	0.21 ± 0.01	0.09 ± 0.02	-0.13 ± 0.02	-0.20 ± 0.02	-0.20 ± 0.02	-0.17 ± 0.02	-0.18 ± 0.02	-0.13 ± 0.02	-0.16 ± 0.02	-0.11 ± 0.02	-0.14 ± 0.02	-0.14 ± 0.02	-0.11 ± 0.02
Bacteria	*Coprothermobacter proteolyticus*	0.05 ± 0.03	0.04 ± 0.03	-0.05 ± 0.03	-0.10 ± 0.03	-0.12 ± 0.03	-0.11 ± 0.03	-0.13 ± 0.03	-0.11 ± 0.03	-0.12 ± 0.03	-0.07 ± 0.03	-0.04 ± 0.03	-0.06 ± 0.03	-0.04 ± 0.03
Bacteria	*Corynebacterium aurimucosum*	0.40 ± 0.02	0.29 ± 0.02	0.05 ± 0.02	-0.01 ± 0.02	-0.10 ± 0.02	-0.06 ± 0.02	-0.06 ± 0.02	-0.08 ± 0.02	-0.08 ± 0.02	-0.10 ± 0.02	-0.08 ± 0.02	-0.09 ± 0.02	-0.12 ± 0.02
Bacteria	*Coxiella burnetii*	0.15 ± 0.02	0.12 ± 0.02	-0.01 ± 0.02	-0.05 ± 0.03	-0.11 ± 0.03	-0.15 ± 0.03	-0.18 ± 0.03	-0.13 ± 0.03	-0.11 ± 0.03	-0.09 ± 0.03	-0.09 ± 0.03	-0.10 ± 0.03	-0.06 ± 0.03
Bacteria	*Cronobacter sakazakii*	0.45 ± 0.02	0.32 ± 0.02	0.07 ± 0.02	0.00 ± 0.02	0.00 ± 0.02	0.01 ± 0.02	-0.02 ± 0.02	-0.03 ± 0.02	-0.07 ± 0.02	-0.09 ± 0.02	-0.10 ± 0.02	-0.10 ± 0.02	-0.10 ± 0.02
Bacteria	*Cupriavidus taiwanensis*	0.82 ± 0.02	0.64 ± 0.02	0.32 ± 0.02	0.15 ± 0.02	0.05 ± 0.02	0.01 ± 0.02	-0.01 ± 0.01	-0.06 ± 0.01	-0.08 ± 0.01	-0.11 ± 0.01	-0.11 ± 0.01	-0.13 ± 0.01	-0.14 ± 0.01
Bacteria	*Cyanothece sp. PCC 7424*	0.17 ± 0.01	0.14 ± 0.01	0.03 ± 0.01	-0.04 ± 0.01	-0.14 ± 0.02	-0.14 ± 0.02	-0.07 ± 0.01	-0.06 ± 0.01	-0.04 ± 0.01	-0.02 ± 0.01	-0.01 ± 0.01	-0.03 ± 0.01	0.01 ± 0.01
Bacteria	*Cytophaga hutchinsonii*	0.26 ± 0.02	0.20 ± 0.02	-0.03 ± 0.02	-0.20 ± 0.02	-0.19 ± 0.02	-0.14 ± 0.02	-0.08 ± 0.02	-0.10 ± 0.02	-0.10 ± 0.02	-0.09 ± 0.02	-0.14 ± 0.02	-0.13 ± 0.02	-0.07 ± 0.02
Bacteria	*Dechloromonas aromatica*	0.54 ± 0.02	0.40 ± 0.02	0.08 ± 0.02	-0.10 ± 0.02	-0.12 ± 0.02	-0.10 ± 0.02	-0.10 ± 0.02	-0.10 ± 0.02	-0.11 ± 0.02	-0.13 ± 0.02	-0.12 ± 0.02	-0.14 ± 0.02	-0.14 ± 0.02
Bacteria	*Dehalococcoides ethenogenes*	0.26 ± 0.02	0.14 ± 0.03	0.04 ± 0.03	-0.12 ± 0.03	-0.22 ± 0.03	-0.22 ± 0.03	-0.15 ± 0.03	-0.14 ± 0.03	-0.18 ± 0.03	-0.13 ± 0.03	-0.18 ± 0.03	-0.17 ± 0.03	-0.19 ± 0.03
Bacteria	*Deinococcus deserti*	0.54 ± 0.02	0.47 ± 0.02	0.18 ± 0.02	0.05 ± 0.02	-0.02 ± 0.02	-0.05 ± 0.02	-0.08 ± 0.02	-0.03 ± 0.02	-0.06 ± 0.02	-0.04 ± 0.02	-0.03 ± 0.02	-0.03 ± 0.02	-0.05 ± 0.02
Bacteria	*Delftia acidovorans*	0.77 ± 0.01	0.58 ± 0.01	0.24 ± 0.01	0.08 ± 0.01	0.03 ± 0.01	-0.01 ± 0.01	-0.04 ± 0.01	-0.08 ± 0.01	-0.11 ± 0.01	-0.12 ± 0.01	-0.13 ± 0.01	-0.13 ± 0.01	-0.16 ± 0.01

Continued on next page

Type	Species	Window 1	Window 2	Window 3	Window 4	Window 5	Window 6	Window 7	Window 8	Window 9	Window 10	Window 11	Window 12	Window 13
Bacteria	*Desulfatibacillum alkenivorans*	0.51 ± 0.01	0.38 ± 0.01	0.16 ± 0.01	0.00 ± 0.01	-0.05 ± 0.01	-0.10 ± 0.01	-0.11 ± 0.01	-0.14 ± 0.01	-0.16 ± 0.01	-0.13 ± 0.01	-0.15 ± 0.01	-0.17 ± 0.01	-0.17 ± 0.01
Bacteria	*Desulfitobacterium hafniense*	0.28 ± 0.01	0.16 ± 0.01	0.07 ± 0.02	0.02 ± 0.02	-0.02 ± 0.02	-0.05 ± 0.02	-0.06 ± 0.01	-0.06 ± 0.01	-0.08 ± 0.01	-0.07 ± 0.01	-0.07 ± 0.01	-0.07 ± 0.01	-0.09 ± 0.01
Bacteria	*Desulfobacterium autotrophicum*	0.33 ± 0.01	0.21 ± 0.01	0.06 ± 0.01	-0.04 ± 0.02	-0.06 ± 0.02	-0.09 ± 0.02	-0.09 ± 0.01	-0.09 ± 0.01	-0.11 ± 0.01	-0.09 ± 0.01	-0.11 ± 0.01	-0.14 ± 0.02	-0.11 ± 0.01
Bacteria	*Desulfococcus oleovorans*	0.51 ± 0.02	0.41 ± 0.02	0.17 ± 0.02	-0.02 ± 0.02	-0.10 ± 0.02	-0.12 ± 0.02	-0.13 ± 0.02	-0.09 ± 0.02	-0.12 ± 0.02	-0.12 ± 0.02	-0.15 ± 0.02	-0.14 ± 0.02	-0.14 ± 0.02
Bacteria	*Desulfotalea psychrophila*	0.30 ± 0.02	0.23 ± 0.02	0.12 ± 0.02	-0.02 ± 0.02	-0.02 ± 0.02	-0.03 ± 0.02	-0.07 ± 0.02	-0.06 ± 0.02	-0.07 ± 0.02	-0.07 ± 0.02	-0.08 ± 0.02	-0.10 ± 0.02	-0.10 ± 0.02
Bacteria	*Desulfotomaculum reducens*	0.11 ± 0.02	0.04 ± 0.02	-0.07 ± 0.02	-0.07 ± 0.02	-0.10 ± 0.02	-0.08 ± 0.02	-0.06 ± 0.02	-0.09 ± 0.02	-0.13 ± 0.02	-0.10 ± 0.02	-0.10 ± 0.02	-0.08 ± 0.02	-0.08 ± 0.02
Bacteria	*Desulfovibrio desulfuricans*	0.38 ± 0.02	0.22 ± 0.02	-0.03 ± 0.02	-0.13 ± 0.02	-0.16 ± 0.02	-0.19 ± 0.02	-0.17 ± 0.02	-0.17 ± 0.02	-0.16 ± 0.02	-0.14 ± 0.02	-0.12 ± 0.02	-0.18 ± 0.02	-0.15 ± 0.02
Bacteria	*Diaphorobacter sp. TPSY*	0.85 ± 0.02	0.68 ± 0.02	0.24 ± 0.02	0.01 ± 0.02	0.01 ± 0.02	-0.06 ± 0.02	-0.07 ± 0.02	-0.09 ± 0.02	-0.13 ± 0.02	-0.15 ± 0.02	-0.17 ± 0.02	-0.18 ± 0.02	-0.16 ± 0.02
Bacteria	*Dichelobacter nodosus*	0.17 ± 0.03	0.05 ± 0.03	-0.12 ± 0.03	-0.10 ± 0.03	-0.14 ± 0.03	-0.17 ± 0.03	-0.13 ± 0.03	-0.11 ± 0.03	-0.12 ± 0.03	-0.12 ± 0.03	-0.12 ± 0.03	-0.13 ± 0.03	-0.12 ± 0.03
Bacteria	*Dickeya dadantii*	0.51 ± 0.02	0.37 ± 0.02	0.05 ± 0.02	0.00 ± 0.02	-0.02 ± 0.02	-0.02 ± 0.02	-0.08 ± 0.02	-0.04 ± 0.02	-0.04 ± 0.02	-0.09 ± 0.02	-0.10 ± 0.02	-0.11 ± 0.02	-0.08 ± 0.02
Bacteria	*Dictyoglomus thermophilum*	-0.01 ± 0.02	0.07 ± 0.02	0.03 ± 0.02	-0.03 ± 0.02	-0.08 ± 0.02	-0.11 ± 0.03	-0.12 ± 0.03	-0.14 ± 0.03	-0.08 ± 0.02	-0.09 ± 0.02	-0.08 ± 0.02	-0.05 ± 0.02	-0.05 ± 0.02
Bacteria	*Dinoroseobacter shibae*	0.67 ± 0.02	0.49 ± 0.02	0.17 ± 0.02	0.03 ± 0.02	0.01 ± 0.02	-0.01 ± 0.02	-0.03 ± 0.02	-0.04 ± 0.02	-0.05 ± 0.02	-0.10 ± 0.02	-0.08 ± 0.02	-0.06 ± 0.02	-0.06 ± 0.02
Bacteria	*Edwardsiella ictaluri*	0.46 ± 0.02	0.34 ± 0.02	0.09 ± 0.02	-0.01 ± 0.02	-0.04 ± 0.02	-0.04 ± 0.02	-0.02 ± 0.02	-0.03 ± 0.02	-0.06 ± 0.02	-0.06 ± 0.02	-0.11 ± 0.02	-0.11 ± 0.02	-0.12 ± 0.02
Bacteria	*Ehrlichia canis*	0.01 ± 0.03	-0.02 ± 0.03	-0.15 ± 0.04	-0.14 ± 0.04	-0.13 ± 0.04	-0.14 ± 0.04	-0.09 ± 0.03	-0.03 ± 0.03	-0.06 ± 0.03	-0.05 ± 0.03	-0.04 ± 0.03	-0.07 ± 0.03	-0.07 ± 0.03
Bacteria	*Elusimicrobium minutum*	0.25 ± 0.02	0.14 ± 0.02	-0.03 ± 0.03	-0.16 ± 0.03	-0.29 ± 0.03	-0.30 ± 0.03	-0.24 ± 0.03	-0.21 ± 0.03	-0.20 ± 0.03	-0.19 ± 0.03	-0.19 ± 0.03	-0.21 ± 0.03	-0.17 ± 0.03
Bacteria	*Enterobacter sp. 638*	0.43 ± 0.02	0.31 ± 0.02	0.01 ± 0.02	-0.06 ± 0.02	-0.06 ± 0.02	-0.05 ± 0.02	-0.04 ± 0.02	-0.03 ± 0.02	-0.04 ± 0.02	-0.10 ± 0.02	-0.09 ± 0.02	-0.10 ± 0.02	-0.12 ± 0.02
Bacteria	*Enterococcus faecalis*	0.15 ± 0.02	0.05 ± 0.02	-0.04 ± 0.02	-0.07 ± 0.02	-0.11 ± 0.02	-0.12 ± 0.02	-0.10 ± 0.02	-0.07 ± 0.02	-0.10 ± 0.02	-0.09 ± 0.02	-0.03 ± 0.02	-0.05 ± 0.02	-0.09 ± 0.02
Bacteria	*Erwinia tasmaniensis*	0.47 ± 0.02	0.33 ± 0.02	0.07 ± 0.02	0.00 ± 0.02	-0.04 ± 0.02	-0.06 ± 0.02	-0.06 ± 0.02	-0.03 ± 0.02	-0.05 ± 0.02	-0.05 ± 0.02	-0.10 ± 0.02	-0.09 ± 0.02	-0.07 ± 0.02
Bacteria	*Erythrobacter litoralis*	0.51 ± 0.02	0.40 ± 0.02	0.10 ± 0.02	-0.09 ± 0.02	-0.11 ± 0.02	-0.13 ± 0.02	-0.09 ± 0.02	-0.12 ± 0.02	-0.11 ± 0.02	-0.08 ± 0.02	-0.09 ± 0.02	-0.12 ± 0.02	-0.13 ± 0.02
Bacteria	*Escherichia coli*	0.35 ± 0.01	0.20 ± 0.02	-0.06 ± 0.02	-0.11 ± 0.02	-0.05 ± 0.02	-0.06 ± 0.02	-0.08 ± 0.02	-0.08 ± 0.02	-0.08 ± 0.02	-0.09 ± 0.02	-0.09 ± 0.02	-0.10 ± 0.02	-0.07 ± 0.02
Bacteria	*Eubacterium eligens*	0.21 ± 0.02	0.06 ± 0.02	-0.14 ± 0.03	-0.16 ± 0.03	-0.15 ± 0.03	-0.16 ± 0.02	-0.14 ± 0.02	-0.17 ± 0.03	-0.16 ± 0.02	-0.18 ± 0.02	-0.16 ± 0.02	-0.15 ± 0.03	-0.15 ± 0.02
Bacteria	*Exiguobacterium sibiricum*	0.41 ± 0.02	0.26 ± 0.02	0.05 ± 0.02	0.00 ± 0.02	-0.03 ± 0.02	-0.03 ± 0.02	-0.03 ± 0.02	-0.06 ± 0.02	-0.03 ± 0.02	-0.06 ± 0.02	-0.08 ± 0.02	-0.09 ± 0.02	-0.12 ± 0.02
Bacteria	*Fervidobacterium nodosum*	0.02 ± 0.02	0.03 ± 0.02	-0.05 ± 0.03	-0.06 ± 0.03	-0.01 ± 0.02	-0.02 ± 0.02	0.01 ± 0.02	-0.03 ± 0.03	-0.08 ± 0.03	-0.07 ± 0.02	-0.06 ± 0.03	-0.03 ± 0.03	-0.03 ± 0.02
Bacteria	*Finegoldia magna*	0.15 ± 0.03	0.04 ± 0.03	-0.10 ± 0.03	-0.14 ± 0.03	-0.13 ± 0.03	-0.12 ± 0.03	-0.13 ± 0.03	-0.15 ± 0.03	-0.13 ± 0.03	-0.05 ± 0.03	-0.13 ± 0.03	-0.13 ± 0.03	-0.07 ± 0.03
Bacteria	*Flavobacterium johnsoniae*	0.26 ± 0.01	0.10 ± 0.01	-0.15 ± 0.02	-0.25 ± 0.02	-0.25 ± 0.02	-0.17 ± 0.02	-0.17 ± 0.02	-0.18 ± 0.02	-0.15 ± 0.02	-0.14 ± 0.02	-0.14 ± 0.02	-0.14 ± 0.02	-0.13 ± 0.02
Bacteria	*Francisella novicida*	0.17 ± 0.02	0.05 ± 0.02	-0.13 ± 0.03	-0.15 ± 0.03	-0.16 ± 0.03	-0.14 ± 0.03	-0.16 ± 0.03	-0.12 ± 0.03	-0.12 ± 0.02	-0.11 ± 0.03	-0.08 ± 0.03	-0.05 ± 0.02	-0.06 ± 0.02
Bacteria	*Frankia alni*	0.27 ± 0.01	0.22 ± 0.01	0.11 ± 0.01	0.04 ± 0.01	0.02 ± 0.01	-0.02 ± 0.01	-0.03 ± 0.01	-0.03 ± 0.01	-0.05 ± 0.01	-0.06 ± 0.01	-0.05 ± 0.01	-0.05 ± 0.01	-0.05 ± 0.01
Bacteria	*Fusobacterium nucleatum*	0.05 ± 0.02	-0.01 ± 0.02	-0.07 ± 0.02	-0.03 ± 0.02	-0.04 ± 0.02	-0.06 ± 0.02	-0.08 ± 0.02	-0.10 ± 0.02	-0.10 ± 0.02	-0.05 ± 0.02	-0.08 ± 0.02	-0.08 ± 0.02	-0.02 ± 0.02
Bacteria	*Gemmatimonas aurantiaca*	0.40 ± 0.02	0.29 ± 0.02	0.04 ± 0.02	-0.05 ± 0.02	-0.07 ± 0.02	-0.07 ± 0.02	-0.07 ± 0.02	-0.07 ± 0.02	-0.09 ± 0.02	-0.12 ± 0.02	-0.10 ± 0.02	-0.12 ± 0.02	-0.14 ± 0.02
Bacteria	*Geobacillus kaustophilus*	0.26 ± 0.02	0.18 ± 0.02	0.01 ± 0.02	0.00 ± 0.02	-0.03 ± 0.02	-0.06 ± 0.02	-0.03 ± 0.02	-0.06 ± 0.02	-0.06 ± 0.02	-0.07 ± 0.02	-0.06 ± 0.02	-0.10 ± 0.02	-0.09 ± 0.02
Bacteria	*Geobacter bemidjiensis*	0.63 ± 0.02	0.51 ± 0.02	0.27 ± 0.02	0.10 ± 0.02	0.01 ± 0.02	0.01 ± 0.02	-0.01 ± 0.02	-0.03 ± 0.02	-0.04 ± 0.02	-0.05 ± 0.02	-0.03 ± 0.02	-0.04 ± 0.02	-0.05 ± 0.02
Bacteria	*Gloeobacter violaceus*	0.48 ± 0.01	0.43 ± 0.02	0.22 ± 0.02	0.09 ± 0.02	0.01 ± 0.02	0.02 ± 0.02	0.01 ± 0.02	-0.01 ± 0.02	-0.02 ± 0.02	-0.04 ± 0.02	-0.03 ± 0.02	-0.05 ± 0.02	-0.06 ± 0.02
Bacteria	*Gluconacetobacter diazotrophicus*	0.64 ± 0.02	0.47 ± 0.02	0.21 ± 0.02	0.11 ± 0.02	0.05 ± 0.02	0.00 ± 0.02	-0.02 ± 0.02	-0.03 ± 0.02	-0.06 ± 0.02	-0.08 ± 0.02	-0.07 ± 0.02	-0.10 ± 0.02	-0.09 ± 0.02
Bacteria	*Gluconobacter oxydans*	0.48 ± 0.02	0.35 ± 0.02	0.09 ± 0.02	0.04 ± 0.02	-0.01 ± 0.02	0.01 ± 0.02	-0.04 ± 0.02	-0.05 ± 0.02	-0.08 ± 0.02	-0.09 ± 0.02	-0.09 ± 0.02	-0.06 ± 0.02	-0.11 ± 0.02
Bacteria	*Gramella forsetii*	0.23 ± 0.02	0.09 ± 0.02	-0.13 ± 0.02	-0.25 ± 0.02	-0.29 ± 0.02	-0.22 ± 0.02	-0.18 ± 0.02	-0.16 ± 0.02	-0.17 ± 0.02	-0.20 ± 0.02	-0.15 ± 0.02	-0.16 ± 0.02	-0.12 ± 0.02
Bacteria	*Granulibacter bethesdensis*	0.30 ± 0.02	0.24 ± 0.02	0.12 ± 0.02	0.00 ± 0.02	-0.02 ± 0.02	-0.02 ± 0.02	-0.04 ± 0.02	-0.04 ± 0.02	-0.07 ± 0.02	-0.07 ± 0.02	-0.11 ± 0.02	-0.07 ± 0.02	-0.07 ± 0.02
Bacteria	*Haemophilus ducreyi*	0.13 ± 0.03	-0.02 ± 0.03	-0.14 ± 0.03	-0.16 ± 0.03	-0.14 ± 0.03	-0.16 ± 0.03	-0.14 ± 0.03	-0.14 ± 0.03	-0.08 ± 0.03	-0.06 ± 0.03	-0.10 ± 0.03	-0.09 ± 0.03	-0.10 ± 0.03
Bacteria	*Hahella chejuensis*	0.38 ± 0.01	0.30 ± 0.01	0.08 ± 0.01	0.01 ± 0.01	-0.02 ± 0.01	-0.06 ± 0.01	-0.07 ± 0.01	-0.08 ± 0.01	-0.09 ± 0.01	-0.10 ± 0.01	-0.10 ± 0.01	-0.11 ± 0.01	-0.08 ± 0.01
Bacteria	*Halorhodospira halophila*	0.58 ± 0.02	0.46 ± 0.02	0.13 ± 0.02	-0.04 ± 0.02	-0.13 ± 0.02	-0.14 ± 0.02	-0.12 ± 0.02	-0.10 ± 0.02	-0.11 ± 0.02	-0.11 ± 0.02	-0.15 ± 0.02	-0.14 ± 0.02	-0.12 ± 0.02

Continued on next page

Type	Species	Window 1	Window 2	Window 3	Window 4	Window 5	Window 6	Window 7	Window 8	Window 9	Window 10	Window 11	Window 12	Window 13
Bacteria	*Halothermothrix orenii*	0.13 ± 0.02	0.08 ± 0.02	-0.02 ± 0.02	0.00 ± 0.02	-0.06 ± 0.02	-0.07 ± 0.02	-0.03 ± 0.02	-0.09 ± 0.02	-0.08 ± 0.02	-0.10 ± 0.02	-0.16 ± 0.02	-0.13 ± 0.02	-0.11 ± 0.02
Bacteria	*Helicobacter acinonychis*	0.19 ± 0.02	0.16 ± 0.02	0.02 ± 0.03	-0.04 ± 0.03	-0.05 ± 0.03	-0.08 ± 0.03	-0.01 ± 0.03	-0.02 ± 0.03	-0.08 ± 0.03	-0.06 ± 0.03	-0.05 ± 0.03	-0.05 ± 0.03	-0.01 ± 0.03
Bacteria	*Heliobacterium modesticaldum*	0.28 ± 0.02	0.15 ± 0.02	0.05 ± 0.02	0.03 ± 0.02	-0.01 ± 0.02	-0.01 ± 0.02	-0.01 ± 0.02	-0.04 ± 0.02	-0.05 ± 0.02	-0.04 ± 0.02	-0.04 ± 0.02	-0.04 ± 0.02	-0.02 ± 0.02
Bacteria	*Herminiimonas arsenicoxydans*	0.51 ± 0.02	0.37 ± 0.02	0.16 ± 0.02	-0.04 ± 0.02	-0.06 ± 0.02	-0.10 ± 0.02	-0.13 ± 0.02	-0.12 ± 0.02	-0.15 ± 0.02	-0.15 ± 0.02	-0.16 ± 0.02	-0.16 ± 0.02	-0.14 ± 0.02
Bacteria	*Herpetosiphon aurantiacus*	0.30 ± 0.01	0.19 ± 0.01	0.02 ± 0.02	-0.03 ± 0.02	-0.05 ± 0.02	-0.07 ± 0.02	-0.09 ± 0.02	-0.08 ± 0.02	-0.07 ± 0.02	-0.07 ± 0.01	-0.08 ± 0.01	-0.08 ± 0.01	-0.09 ± 0.01
Bacteria	*Hydrogenobaculum sp. Y04AAS1*	-0.03 ± 0.03	-0.03 ± 0.03	-0.12 ± 0.03	-0.13 ± 0.03	-0.20 ± 0.03	-0.19 ± 0.03	-0.19 ± 0.03	-0.12 ± 0.03	-0.13 ± 0.03	-0.09 ± 0.03	-0.08 ± 0.03	-0.08 ± 0.03	-0.06 ± 0.02
Bacteria	*Hyphomonas neptunium*	0.57 ± 0.02	0.43 ± 0.02	0.10 ± 0.02	-0.09 ± 0.02	-0.11 ± 0.02	-0.10 ± 0.02	-0.09 ± 0.02	-0.08 ± 0.02	-0.12 ± 0.02	-0.11 ± 0.02	-0.10 ± 0.02	-0.11 ± 0.02	-0.12 ± 0.02
Bacteria	*Idiomarina loihiensis*	0.26 ± 0.02	0.14 ± 0.02	-0.11 ± 0.02	-0.25 ± 0.02	-0.22 ± 0.02	-0.16 ± 0.02	-0.15 ± 0.02	-0.13 ± 0.02	-0.15 ± 0.02	-0.19 ± 0.02	-0.18 ± 0.02	-0.15 ± 0.02	-0.15 ± 0.02
Bacteria	*Jannaschia sp. CCS1*	0.61 ± 0.01	0.46 ± 0.02	0.15 ± 0.02	0.00 ± 0.02	-0.03 ± 0.02	-0.05 ± 0.02	-0.02 ± 0.02	-0.02 ± 0.02	-0.04 ± 0.02	-0.04 ± 0.02	-0.05 ± 0.02	-0.03 ± 0.02	-0.04 ± 0.02
Bacteria	*Janthinobacterium sp. Marseille*	0.54 ± 0.02	0.39 ± 0.02	0.12 ± 0.02	-0.08 ± 0.02	-0.08 ± 0.02	-0.09 ± 0.02	-0.08 ± 0.02	-0.12 ± 0.02	-0.12 ± 0.02	-0.12 ± 0.02	-0.13 ± 0.02	-0.16 ± 0.02	-0.14 ± 0.02
Bacteria	*Kineococcus radiotolerans*	0.44 ± 0.02	0.37 ± 0.02	0.16 ± 0.02	0.09 ± 0.02	0.04 ± 0.02	0.02 ± 0.02	-0.01 ± 0.02	-0.02 ± 0.02	-0.02 ± 0.02	-0.04 ± 0.02	-0.06 ± 0.02	-0.04 ± 0.02	-0.05 ± 0.02
Bacteria	*Klebsiella pneumoniae*	0.60 ± 0.01	0.42 ± 0.01	0.13 ± 0.02	0.04 ± 0.02	0.01 ± 0.02	-0.01 ± 0.02	-0.03 ± 0.02	-0.05 ± 0.01	-0.05 ± 0.01	-0.09 ± 0.01	-0.08 ± 0.01	-0.09 ± 0.01	-0.08 ± 0.01
Bacteria	*Kocuria rhizophila*	0.60 ± 0.02	0.50 ± 0.02	0.19 ± 0.02	0.06 ± 0.02	-0.03 ± 0.02	-0.06 ± 0.02	-0.04 ± 0.02	-0.06 ± 0.02	-0.06 ± 0.02	-0.10 ± 0.02	-0.13 ± 0.02	-0.13 ± 0.02	-0.14 ± 0.02
Bacteria	*Kosmotoga olearia*	0.04 ± 0.02	0.05 ± 0.02	0.02 ± 0.02	-0.01 ± 0.02	-0.04 ± 0.02	-0.06 ± 0.02	-0.11 ± 0.02	-0.10 ± 0.02	-0.10 ± 0.02	-0.11 ± 0.02	-0.06 ± 0.02	-0.09 ± 0.02	-0.07 ± 0.02
Bacteria	*Lactobacillus acidophilus*	0.15 ± 0.02	0.04 ± 0.03	-0.14 ± 0.03	-0.11 ± 0.03	-0.16 ± 0.03	-0.14 ± 0.03	-0.11 ± 0.02	-0.10 ± 0.02	-0.09 ± 0.02	-0.09 ± 0.02	-0.12 ± 0.03	-0.09 ± 0.02	-0.09 ± 0.02
Bacteria	*Lactococcus lactis*	0.19 ± 0.02	0.03 ± 0.02	-0.19 ± 0.02	-0.19 ± 0.02	-0.13 ± 0.02	-0.15 ± 0.02	-0.12 ± 0.02	-0.13 ± 0.02	-0.14 ± 0.02	-0.08 ± 0.02	-0.10 ± 0.02	-0.13 ± 0.02	-0.14 ± 0.02
Bacteria	*Laribacter hongkongensis*	0.58 ± 0.02	0.42 ± 0.02	0.06 ± 0.02	-0.04 ± 0.02	-0.08 ± 0.02	-0.09 ± 0.02	-0.14 ± 0.02	-0.12 ± 0.02	-0.12 ± 0.02	-0.10 ± 0.02	-0.10 ± 0.02	-0.15 ± 0.02	-0.14 ± 0.02
Bacteria	*Lawsonia intracellularis*	-0.07 ± 0.03	-0.04 ± 0.03	-0.12 ± 0.03	-0.18 ± 0.03	-0.18 ± 0.03	-0.13 ± 0.03	-0.10 ± 0.03	-0.06 ± 0.03	-0.07 ± 0.03	-0.06 ± 0.03	-0.02 ± 0.03	-0.08 ± 0.03	-0.09 ± 0.03
Bacteria	*Legionella pneumophila*	0.14 ± 0.02	0.09 ± 0.02	-0.02 ± 0.02	-0.08 ± 0.02	-0.12 ± 0.02	-0.11 ± 0.02	-0.06 ± 0.02	-0.08 ± 0.02	-0.09 ± 0.02	-0.08 ± 0.02	-0.06 ± 0.02	-0.05 ± 0.02	-0.07 ± 0.02
Bacteria	*Leifsonia xyli*	0.45 ± 0.02	0.31 ± 0.02	0.10 ± 0.03	0.07 ± 0.02	0.02 ± 0.02	0.03 ± 0.02	-0.01 ± 0.02	0.01 ± 0.02	0.03 ± 0.02	-0.02 ± 0.02	-0.06 ± 0.02	-0.08 ± 0.02	-0.02 ± 0.02
Bacteria	*Leptospira biflexa*	0.14 ± 0.02	0.10 ± 0.02	-0.05 ± 0.02	-0.23 ± 0.02	-0.30 ± 0.02	-0.25 ± 0.02	-0.19 ± 0.02	-0.18 ± 0.02	-0.15 ± 0.02	-0.16 ± 0.02	-0.18 ± 0.02	-0.18 ± 0.02	-0.19 ± 0.02
Bacteria	*Leptothrix cholodnii*	0.87 ± 0.02	0.68 ± 0.02	0.24 ± 0.02	0.06 ± 0.02	0.02 ± 0.02	-0.03 ± 0.02	-0.08 ± 0.02	-0.11 ± 0.02	-0.14 ± 0.02	-0.15 ± 0.02	-0.13 ± 0.02	-0.14 ± 0.02	-0.15 ± 0.02
Bacteria	*Leuconostoc citreum*	0.23 ± 0.02	0.12 ± 0.02	-0.11 ± 0.03	-0.12 ± 0.03	-0.12 ± 0.03	-0.13 ± 0.03	-0.13 ± 0.03	-0.11 ± 0.03	-0.11 ± 0.03	-0.13 ± 0.02	-0.11 ± 0.03	-0.16 ± 0.03	-0.10 ± 0.03
Bacteria	*Listeria innocua*	0.17 ± 0.02	0.15 ± 0.02	0.03 ± 0.02	-0.02 ± 0.02	-0.02 ± 0.02	-0.02 ± 0.02	-0.01 ± 0.02	-0.01 ± 0.02	-0.02 ± 0.02	0.01 ± 0.02	0.01 ± 0.02	0.02 ± 0.02	-0.01 ± 0.02
Bacteria	*Lysinibacillus sphaericus*	0.09 ± 0.01	0.09 ± 0.01	0.02 ± 0.02	0.00 ± 0.02	-0.05 ± 0.02	-0.07 ± 0.02	-0.05 ± 0.02	-0.03 ± 0.02	-0.06 ± 0.02	-0.05 ± 0.02	-0.05 ± 0.02	-0.05 ± 0.02	-0.06 ± 0.02
Bacteria	*Macrococcus caseolyticus*	0.23 ± 0.02	0.17 ± 0.02	0.06 ± 0.02	0.00 ± 0.02	-0.02 ± 0.02	-0.01 ± 0.02	-0.07 ± 0.02	-0.03 ± 0.02	-0.07 ± 0.02	-0.03 ± 0.02	-0.05 ± 0.02	-0.09 ± 0.02	-0.10 ± 0.02
Bacteria	*Magnetococcus sp. MC-1*	0.31 ± 0.02	0.21 ± 0.02	0.01 ± 0.02	-0.12 ± 0.02	-0.14 ± 0.02	-0.12 ± 0.02	-0.07 ± 0.02	-0.10 ± 0.02	-0.11 ± 0.02	-0.13 ± 0.02	-0.08 ± 0.02	-0.14 ± 0.02	-0.15 ± 0.02
Bacteria	*Magnetospirillum magneticum*	0.46 ± 0.02	0.40 ± 0.02	0.17 ± 0.02	0.00 ± 0.02	-0.04 ± 0.02	-0.04 ± 0.02	-0.06 ± 0.02	-0.06 ± 0.02	-0.09 ± 0.02	-0.11 ± 0.02	-0.12 ± 0.02	-0.13 ± 0.02	-0.12 ± 0.02
Bacteria	*Mannheimia succiniciproducens*	0.25 ± 0.02	0.14 ± 0.02	-0.05 ± 0.02	-0.06 ± 0.02	-0.10 ± 0.02	-0.12 ± 0.02	-0.14 ± 0.02	-0.12 ± 0.02	-0.12 ± 0.02	-0.10 ± 0.02	-0.14 ± 0.02	-0.14 ± 0.02	-0.13 ± 0.02
Bacteria	*Maricaulis maris*	0.55 ± 0.02	0.43 ± 0.02	0.11 ± 0.02	-0.01 ± 0.02	-0.08 ± 0.02	-0.09 ± 0.02	-0.08 ± 0.02	-0.11 ± 0.02	-0.14 ± 0.02	-0.13 ± 0.02	-0.17 ± 0.02	-0.12 ± 0.02	-0.12 ± 0.02
Bacteria	*Marinobacter aquaeolei*	0.45 ± 0.02	0.33 ± 0.02	0.06 ± 0.02	-0.06 ± 0.02	-0.08 ± 0.02	-0.10 ± 0.02	-0.11 ± 0.02	-0.11 ± 0.02	-0.07 ± 0.02	-0.11 ± 0.02	-0.15 ± 0.02	-0.10 ± 0.02	-0.11 ± 0.02
Bacteria	*Marinomonas sp. MWYL1*	0.21 ± 0.01	0.09 ± 0.02	-0.11 ± 0.02	-0.21 ± 0.02	-0.17 ± 0.02	-0.19 ± 0.02	-0.13 ± 0.02	-0.13 ± 0.02	-0.12 ± 0.02	-0.10 ± 0.02	-0.10 ± 0.02	-0.11 ± 0.02	-0.12 ± 0.02
Bacteria	*Mesoplasma florum*	0.11 ± 0.08	-0.07 ± 0.10	-0.39 ± 0.11	-0.31 ± 0.11	-0.25 ± 0.16	-0.17 ± 0.09	-0.33 ± 0.16	-0.48 ± 0.26	-0.20 ± 0.11	-0.21 ± 0.11	-0.15 ± 0.10	-0.12 ± 0.09	-0.17 ± 0.10
Bacteria	*Mesorhizobium loti*	0.45 ± 0.01	0.38 ± 0.01	0.15 ± 0.01	0.00 ± 0.01	-0.04 ± 0.01	-0.08 ± 0.01	-0.09 ± 0.01	-0.09 ± 0.01	-0.09 ± 0.01	-0.10 ± 0.01	-0.10 ± 0.01	-0.08 ± 0.01	-0.11 ± 0.01
Bacteria	*Methylacidiphilum infernorum*	0.09 ± 0.02	0.11 ± 0.02	0.06 ± 0.02	0.01 ± 0.02	-0.06 ± 0.02	-0.06 ± 0.02	-0.04 ± 0.02	-0.07 ± 0.02	-0.04 ± 0.02	-0.06 ± 0.02	-0.07 ± 0.02	-0.07 ± 0.02	-0.06 ± 0.02
Bacteria	*Methylibium petroleiphilum*	0.74 ± 0.02	0.61 ± 0.02	0.21 ± 0.02	0.04 ± 0.02	-0.04 ± 0.02	-0.05 ± 0.02	-0.10 ± 0.02	-0.11 ± 0.02	-0.14 ± 0.02	-0.14 ± 0.02	-0.09 ± 0.02	-0.12 ± 0.02	-0.11 ± 0.02
Bacteria	*Methylobacillus flagellatus*	0.46 ± 0.02	0.31 ± 0.02	0.06 ± 0.02	-0.06 ± 0.02	-0.11 ± 0.02	-0.11 ± 0.02	-0.08 ± 0.02	-0.15 ± 0.02	-0.15 ± 0.02	-0.13 ± 0.02	-0.15 ± 0.02	-0.10 ± 0.02	-0.09 ± 0.02
Bacteria	*Methylobacterium chloromethanicum*	0.58 ± 0.01	0.45 ± 0.01	0.18 ± 0.02	0.01 ± 0.02	-0.03 ± 0.01	-0.05 ± 0.01	-0.05 ± 0.01	-0.06 ± 0.01	-0.09 ± 0.01	-0.09 ± 0.01	-0.06 ± 0.01	-0.09 ± 0.01	-0.08 ± 0.01
Bacteria	*Methylocella silvestris*	0.45 ± 0.02	0.36 ± 0.02	0.11 ± 0.02	0.02 ± 0.02	-0.04 ± 0.02	-0.07 ± 0.02	-0.11 ± 0.02	-0.08 ± 0.02	-0.07 ± 0.02	-0.06 ± 0.02	-0.09 ± 0.02	-0.11 ± 0.02	-0.11 ± 0.02

Continued on next page

Type	Species	Window 1	Window 2	Window 3	Window 4	Window 5	Window 6	Window 7	Window 8	Window 9	Window 10	Window 11	Window 12	Window 13
Bacteria	*Methylococcus capsulatus*	0.48 ± 0.02	0.36 ± 0.02	0.10 ± 0.02	0.01 ± 0.02	0.00 ± 0.02	-0.04 ± 0.02	-0.05 ± 0.02	-0.03 ± 0.02	-0.05 ± 0.02	-0.08 ± 0.02	-0.06 ± 0.02	-0.06 ± 0.02	-0.08 ± 0.02
Bacteria	*Micrococcus luteus*	0.67 ± 0.02	0.50 ± 0.02	0.23 ± 0.02	0.09 ± 0.02	0.01 ± 0.02	0.00 ± 0.02	-0.02 ± 0.02	-0.03 ± 0.02	-0.06 ± 0.02	-0.06 ± 0.02	-0.07 ± 0.02	-0.05 ± 0.02	-0.04 ± 0.02
Bacteria	*Microcystis aeruginosa*	0.17 ± 0.01	0.15 ± 0.01	0.04 ± 0.01	-0.05 ± 0.01	-0.16 ± 0.01	-0.14 ± 0.01	-0.09 ± 0.01	-0.08 ± 0.01	-0.05 ± 0.01	-0.03 ± 0.01	-0.01 ± 0.01	-0.02 ± 0.01	-0.04 ± 0.01
Bacteria	*Moorella thermoacetica*	0.26 ± 0.02	0.15 ± 0.02	-0.04 ± 0.02	-0.10 ± 0.02	-0.14 ± 0.02	-0.15 ± 0.02	-0.14 ± 0.02	-0.12 ± 0.02	-0.16 ± 0.02	-0.15 ± 0.02	-0.16 ± 0.02	-0.12 ± 0.02	-0.14 ± 0.02
Bacteria	*Mycobacterium avium*	0.38 ± 0.02	0.26 ± 0.02	0.07 ± 0.02	0.01 ± 0.01	-0.01 ± 0.02	-0.03 ± 0.01	-0.03 ± 0.01	-0.03 ± 0.01	-0.07 ± 0.01	-0.09 ± 0.01	-0.08 ± 0.01	-0.09 ± 0.01	-0.07 ± 0.01
Bacteria	*Mycoplasma agalactiae*	0.10 ± 0.07	0.15 ± 0.08	-0.17 ± 0.09	-0.26 ± 0.10	-0.01 ± 0.08	-0.07 ± 0.08	-0.16 ± 0.09	-0.22 ± 0.10	-0.19 ± 0.08	-0.20 ± 0.09	-0.23 ± 0.10	-0.16 ± 0.09	-0.08 ± 0.09
Bacteria	*Myxococcus xanthus*	0.55 ± 0.01	0.54 ± 0.01	0.36 ± 0.01	0.20 ± 0.01	0.11 ± 0.01	0.10 ± 0.01	0.09 ± 0.01	0.06 ± 0.01	0.03 ± 0.01	-0.02 ± 0.01	0.00 ± 0.01	-0.01 ± 0.01	-0.03 ± 0.01
Bacteria	*Natranaerobius thermophilus*	-0.02 ± 0.02	0.02 ± 0.02	-0.04 ± 0.02	-0.04 ± 0.02	-0.06 ± 0.02	-0.11 ± 0.02	-0.13 ± 0.02	-0.05 ± 0.02	-0.04 ± 0.02	-0.08 ± 0.02	-0.07 ± 0.02	-0.09 ± 0.02	-0.05 ± 0.02
Bacteria	*Nautilia profundicola*	0.08 ± 0.02	0.02 ± 0.03	-0.13 ± 0.03	-0.17 ± 0.03	-0.13 ± 0.03	-0.12 ± 0.03	-0.12 ± 0.03	-0.11 ± 0.03	-0.07 ± 0.03	-0.11 ± 0.03	-0.05 ± 0.03	-0.08 ± 0.03	-0.09 ± 0.03
Bacteria	*Neisseria gonorrhoeae*	0.36 ± 0.02	0.25 ± 0.02	0.08 ± 0.02	-0.04 ± 0.02	-0.06 ± 0.03	-0.08 ± 0.03	-0.04 ± 0.02	-0.08 ± 0.02	-0.10 ± 0.02	-0.09 ± 0.02	-0.09 ± 0.02	-0.07 ± 0.02	-0.11 ± 0.02
Bacteria	*Neorickettsia sennetsu*	0.08 ± 0.03	0.03 ± 0.03	-0.09 ± 0.04	-0.13 ± 0.04	-0.20 ± 0.04	-0.14 ± 0.04	-0.15 ± 0.04	-0.11 ± 0.04	-0.10 ± 0.04	-0.06 ± 0.04	-0.11 ± 0.04	-0.06 ± 0.04	-0.13 ± 0.04
Bacteria	*Nitratiruptor sp. SB155-2*	0.11 ± 0.02	0.08 ± 0.02	-0.08 ± 0.03	-0.07 ± 0.03	-0.10 ± 0.03	-0.11 ± 0.02	-0.09 ± 0.02	-0.08 ± 0.02	-0.09 ± 0.02	-0.07 ± 0.02	-0.10 ± 0.02	-0.11 ± 0.02	-0.07 ± 0.02
Bacteria	*Nitrobacter hamburgensis*	0.48 ± 0.02	0.41 ± 0.02	0.16 ± 0.02	0.00 ± 0.02	-0.05 ± 0.02	-0.08 ± 0.02	-0.09 ± 0.02	-0.07 ± 0.02	-0.09 ± 0.02	-0.11 ± 0.02	-0.08 ± 0.02	-0.11 ± 0.02	-0.12 ± 0.02
Bacteria	*Nitrosococcus oceani*	0.26 ± 0.02	0.16 ± 0.02	-0.02 ± 0.02	-0.08 ± 0.02	-0.10 ± 0.02	-0.12 ± 0.02	-0.08 ± 0.02	-0.11 ± 0.02	-0.08 ± 0.02	-0.09 ± 0.02	-0.09 ± 0.02	-0.06 ± 0.02	-0.08 ± 0.02
Bacteria	*Nitrosomonas europaea*	0.27 ± 0.02	0.21 ± 0.02	-0.01 ± 0.02	-0.10 ± 0.02	-0.14 ± 0.02	-0.09 ± 0.02	-0.10 ± 0.02	-0.09 ± 0.02	-0.12 ± 0.02	-0.11 ± 0.02	-0.19 ± 0.02	-0.13 ± 0.02	-0.11 ± 0.02
Bacteria	*Nitrosospira multiformis*	0.35 ± 0.02	0.25 ± 0.02	0.12 ± 0.02	0.02 ± 0.02	-0.03 ± 0.02	-0.01 ± 0.02	-0.04 ± 0.02	-0.03 ± 0.02	-0.04 ± 0.02	-0.07 ± 0.02	-0.10 ± 0.02	-0.08 ± 0.02	-0.05 ± 0.02
Bacteria	*Nocardia farcinica*	0.51 ± 0.01	0.36 ± 0.01	0.15 ± 0.01	0.06 ± 0.01	0.01 ± 0.01	-0.01 ± 0.01	-0.03 ± 0.01	-0.03 ± 0.01	-0.07 ± 0.01	-0.07 ± 0.01	-0.08 ± 0.01	-0.07 ± 0.01	-0.09 ± 0.01
Bacteria	*Nocardioides sp. JS614*	0.49 ± 0.02	0.27 ± 0.02	0.08 ± 0.02	-0.01 ± 0.02	-0.05 ± 0.02	-0.06 ± 0.02	-0.05 ± 0.02	-0.06 ± 0.02	-0.06 ± 0.02	-0.13 ± 0.01	-0.11 ± 0.02	-0.10 ± 0.01	-0.12 ± 0.01
Bacteria	*Nostoc punctiforme*	0.16 ± 0.01	0.12 ± 0.01	0.01 ± 0.01	-0.11 ± 0.01	-0.14 ± 0.01	-0.16 ± 0.01	-0.13 ± 0.01	-0.10 ± 0.01	-0.09 ± 0.01	-0.12 ± 0.01	-0.07 ± 0.01	-0.07 ± 0.01	-0.07 ± 0.01
Bacteria	*Novosphingobium aromaticivorans*	0.56 ± 0.02	0.42 ± 0.02	0.12 ± 0.02	0.00 ± 0.02	-0.03 ± 0.02	-0.06 ± 0.02	-0.05 ± 0.02	-0.05 ± 0.02	-0.01 ± 0.02	-0.06 ± 0.02	-0.09 ± 0.02	-0.11 ± 0.02	-0.08 ± 0.02
Bacteria	*Oceanobacillus iheyensis*	0.08 ± 0.02	0.09 ± 0.02	-0.01 ± 0.02	-0.02 ± 0.02	-0.03 ± 0.02	-0.04 ± 0.02	-0.03 ± 0.02	-0.03 ± 0.02	-0.05 ± 0.02	-0.05 ± 0.02	-0.04 ± 0.02	-0.04 ± 0.02	-0.05 ± 0.02
Bacteria	*Ochrobactrum anthropi*	0.43 ± 0.01	0.31 ± 0.02	0.07 ± 0.02	-0.07 ± 0.02	-0.11 ± 0.02	-0.13 ± 0.02	-0.11 ± 0.02	-0.16 ± 0.02	-0.13 ± 0.02	-0.13 ± 0.02	-0.11 ± 0.02	-0.16 ± 0.02	-0.19 ± 0.02
Bacteria	*Oenococcus oeni*	0.24 ± 0.02	0.15 ± 0.02	0.02 ± 0.03	-0.09 ± 0.03	-0.13 ± 0.03	-0.11 ± 0.03	-0.09 ± 0.03	-0.13 ± 0.03	-0.12 ± 0.03	-0.10 ± 0.03	-0.09 ± 0.03	-0.10 ± 0.03	-0.11 ± 0.03
Bacteria	*Oligotropha carboxidovorans*	0.52 ± 0.02	0.41 ± 0.02	0.18 ± 0.02	-0.03 ± 0.02	-0.08 ± 0.02	-0.08 ± 0.02	-0.10 ± 0.02	-0.09 ± 0.02	-0.11 ± 0.02	-0.15 ± 0.02	-0.10 ± 0.02	-0.16 ± 0.02	-0.15 ± 0.02
Bacteria	*Onion yellows*	0.04 ± 0.03	-0.07 ± 0.04	-0.09 ± 0.04	-0.10 ± 0.04	-0.06 ± 0.04	-0.01 ± 0.04	-0.03 ± 0.04	-0.03 ± 0.04	0.01 ± 0.03	-0.07 ± 0.04	-0.10 ± 0.04	-0.03 ± 0.04	0.00 ± 0.03
Bacteria	*Opitutus terrae*	0.78 ± 0.01	0.66 ± 0.02	0.31 ± 0.02	0.04 ± 0.02	-0.03 ± 0.02	-0.03 ± 0.02	-0.03 ± 0.02	-0.05 ± 0.02	-0.05 ± 0.02	-0.08 ± 0.02	-0.10 ± 0.02	-0.11 ± 0.02	-0.08 ± 0.02
Bacteria	*Orientia tsutsugamushi*	-0.15 ± 0.03	-0.19 ± 0.04	-0.14 ± 0.03	-0.11 ± 0.03	-0.14 ± 0.03	-0.10 ± 0.03	-0.06 ± 0.03	-0.11 ± 0.03	-0.08 ± 0.03	-0.14 ± 0.03	-0.12 ± 0.03	-0.09 ± 0.03	-0.14 ± 0.03
Bacteria	*Parabacteroides distasonis*	0.52 ± 0.01	0.26 ± 0.02	-0.11 ± 0.02	-0.27 ± 0.02	-0.16 ± 0.02	-0.12 ± 0.02	-0.11 ± 0.02	-0.11 ± 0.02	-0.13 ± 0.02	-0.08 ± 0.02	-0.11 ± 0.02	-0.13 ± 0.02	-0.10 ± 0.02
Bacteria	*Paracoccus denitrificans*	0.64 ± 0.02	0.46 ± 0.02	0.22 ± 0.02	0.03 ± 0.02	0.01 ± 0.02	-0.03 ± 0.02	-0.08 ± 0.02	-0.06 ± 0.02	-0.09 ± 0.02	-0.10 ± 0.02	-0.09 ± 0.02	-0.09 ± 0.02	-0.11 ± 0.02
Bacteria	*Parvibaculum lavamentivorans*	0.54 ± 0.02	0.40 ± 0.02	0.12 ± 0.02	-0.08 ± 0.02	-0.14 ± 0.02	-0.11 ± 0.02	-0.08 ± 0.02	-0.10 ± 0.02	-0.14 ± 0.02	-0.12 ± 0.02	-0.12 ± 0.02	-0.11 ± 0.02	-0.10 ± 0.02
Bacteria	*Pasteurella multocida*	0.24 ± 0.02	0.10 ± 0.02	-0.10 ± 0.02	-0.17 ± 0.03	-0.19 ± 0.03	-0.11 ± 0.02	-0.10 ± 0.02	-0.08 ± 0.02	-0.08 ± 0.02	-0.08 ± 0.02	-0.02 ± 0.02	-0.08 ± 0.02	-0.05 ± 0.02
Bacteria	*Pectobacterium atrosepticum*	0.39 ± 0.01	0.28 ± 0.02	0.05 ± 0.02	-0.01 ± 0.02	-0.05 ± 0.02	-0.03 ± 0.02	-0.05 ± 0.02	-0.06 ± 0.02	-0.07 ± 0.02	-0.09 ± 0.02	-0.09 ± 0.02	-0.08 ± 0.02	-0.09 ± 0.02
Bacteria	*Pediococcus pentosaceus*	0.19 ± 0.02	0.11 ± 0.02	-0.10 ± 0.03	-0.09 ± 0.03	-0.11 ± 0.03	-0.15 ± 0.03	-0.15 ± 0.03	-0.11 ± 0.03	-0.08 ± 0.03	-0.11 ± 0.02	-0.11 ± 0.03	-0.12 ± 0.03	-0.12 ± 0.03
Bacteria	*Pelobacter carbinolicus*	0.38 ± 0.02	0.27 ± 0.02	0.10 ± 0.02	-0.02 ± 0.02	-0.09 ± 0.02	-0.13 ± 0.02	-0.10 ± 0.02	-0.10 ± 0.02	-0.11 ± 0.02	-0.11 ± 0.02	-0.09 ± 0.02	-0.12 ± 0.02	-0.12 ± 0.02
Bacteria	*Pelodictyon luteolum*	0.48 ± 0.02	0.32 ± 0.02	0.03 ± 0.02	-0.11 ± 0.02	-0.06 ± 0.02	-0.03 ± 0.02	-0.08 ± 0.02	-0.05 ± 0.02	-0.03 ± 0.02	-0.09 ± 0.02	-0.08 ± 0.02	-0.11 ± 0.02	-0.10 ± 0.02
Bacteria	*Pelotomaculum thermopropionicum*	0.26 ± 0.02	0.20 ± 0.02	0.04 ± 0.02	0.00 ± 0.02	-0.02 ± 0.02	-0.12 ± 0.02	-0.08 ± 0.02	-0.06 ± 0.02	-0.09 ± 0.02	-0.09 ± 0.02	-0.06 ± 0.02	-0.08 ± 0.02	-0.07 ± 0.02
Bacteria	*Persephonella marina*	0.09 ± 0.02	0.01 ± 0.02	-0.07 ± 0.02	-0.11 ± 0.02	-0.11 ± 0.02	-0.13 ± 0.02	-0.09 ± 0.02	-0.13 ± 0.02	-0.13 ± 0.02	-0.10 ± 0.02	-0.09 ± 0.02	-0.09 ± 0.02	-0.09 ± 0.02
Bacteria	*Petrotoga mobilis*	0.01 ± 0.02	0.05 ± 0.02	-0.02 ± 0.02	-0.02 ± 0.03	-0.01 ± 0.02	-0.02 ± 0.02	-0.03 ± 0.02	-0.09 ± 0.03	-0.09 ± 0.02	-0.09 ± 0.02	-0.06 ± 0.02	0.02 ± 0.02	-0.03 ± 0.02
Bacteria	*Phenylobacterium zucineum*	0.66 ± 0.02	0.52 ± 0.02	0.21 ± 0.02	0.00 ± 0.02	-0.01 ± 0.02	0.00 ± 0.02	-0.03 ± 0.02	-0.07 ± 0.02	-0.05 ± 0.02	-0.08 ± 0.02	-0.10 ± 0.02	-0.07 ± 0.02	-0.06 ± 0.02

Continued on next page

Type	Species	Window 1	Window 2	Window 3	Window 4	Window 5	Window 6	Window 7	Window 8	Window 9	Window 10	Window 11	Window 12	Window 13
Bacteria	*Photobacterium profundum*	0.11 ± 0.01	0.04 ± 0.01	−0.10 ± 0.02	−0.11 ± 0.01	−0.14 ± 0.01	−0.11 ± 0.02	−0.06 ± 0.01	−0.09 ± 0.01	−0.11 ± 0.01	−0.12 ± 0.01	−0.12 ± 0.01	−0.10 ± 0.01	−0.15 ± 0.01
Bacteria	*Photorhabdus luminescens*	0.18 ± 0.01	0.08 ± 0.02	−0.07 ± 0.02	−0.10 ± 0.02	−0.10 ± 0.02	−0.12 ± 0.02	−0.10 ± 0.02	−0.11 ± 0.02	−0.10 ± 0.02	−0.10 ± 0.02	−0.08 ± 0.02	−0.08 ± 0.02	−0.08 ± 0.02
Bacteria	*Polaromonas naphthalenivorans*	0.78 ± 0.02	0.61 ± 0.02	0.25 ± 0.02	0.10 ± 0.02	0.02 ± 0.02	−0.03 ± 0.02	−0.04 ± 0.02	−0.07 ± 0.02	−0.13 ± 0.02	−0.14 ± 0.02	−0.13 ± 0.02	−0.13 ± 0.02	−0.15 ± 0.02
Bacteria	*Polynucleobacter necessarius*	0.27 ± 0.02	0.16 ± 0.02	−0.11 ± 0.03	−0.37 ± 0.03	−0.38 ± 0.03	−0.28 ± 0.02	−0.22 ± 0.02	−0.20 ± 0.02	−0.22 ± 0.02	−0.14 ± 0.02	−0.15 ± 0.02	−0.17 ± 0.02	−0.16 ± 0.02
Bacteria	*Porphyromonas gingivalis*	0.35 ± 0.02	0.15 ± 0.02	−0.17 ± 0.03	−0.20 ± 0.03	−0.18 ± 0.02	−0.18 ± 0.02	−0.12 ± 0.02	−0.09 ± 0.02	−0.17 ± 0.02	−0.09 ± 0.02	−0.14 ± 0.02	−0.17 ± 0.03	−0.23 ± 0.03
Bacteria	*Prochlorococcus marinus*	0.03 ± 0.02	0.04 ± 0.02	−0.10 ± 0.02	−0.16 ± 0.03	−0.10 ± 0.03	−0.10 ± 0.02	−0.08 ± 0.02	−0.03 ± 0.02	−0.03 ± 0.02	−0.05 ± 0.02	−0.04 ± 0.02	−0.06 ± 0.02	−0.04 ± 0.02
Bacteria	*Propionibacterium acnes*	0.17 ± 0.02	0.06 ± 0.02	−0.08 ± 0.02	−0.09 ± 0.02	−0.09 ± 0.02	−0.06 ± 0.02	−0.08 ± 0.02	−0.06 ± 0.02	−0.08 ± 0.02	−0.08 ± 0.02	−0.08 ± 0.02	−0.08 ± 0.02	−0.10 ± 0.02
Bacteria	*Prosthecochloris aestuarii*	0.34 ± 0.02	0.20 ± 0.02	−0.01 ± 0.02	−0.11 ± 0.02	−0.18 ± 0.02	−0.06 ± 0.02	−0.10 ± 0.02	−0.09 ± 0.02	−0.13 ± 0.02	−0.10 ± 0.02	−0.11 ± 0.02	−0.11 ± 0.02	−0.08 ± 0.02
Bacteria	*Proteus mirabilis*	0.10 ± 0.02	−0.06 ± 0.02	−0.21 ± 0.02	−0.22 ± 0.02	−0.19 ± 0.02	−0.20 ± 0.02	−0.15 ± 0.02	−0.14 ± 0.02	−0.15 ± 0.02	−0.16 ± 0.02	−0.12 ± 0.02	−0.09 ± 0.02	−0.10 ± 0.02
Bacteria	*Pseudoalteromonas atlantica*	0.24 ± 0.01	0.16 ± 0.02	−0.05 ± 0.02	−0.11 ± 0.02	−0.12 ± 0.02	−0.12 ± 0.02	−0.07 ± 0.02	−0.09 ± 0.02	−0.11 ± 0.02	−0.12 ± 0.02	−0.14 ± 0.02	−0.12 ± 0.02	−0.11 ± 0.02
Bacteria	*Pseudomonas aeruginosa*	0.70 ± 0.01	0.53 ± 0.01	0.22 ± 0.01	0.10 ± 0.01	0.05 ± 0.01	0.01 ± 0.01	−0.02 ± 0.01	−0.07 ± 0.01	−0.10 ± 0.01	−0.10 ± 0.01	−0.12 ± 0.01	−0.09 ± 0.01	−0.12 ± 0.01
Bacteria	*Psychrobacter arcticus*	0.36 ± 0.02	0.22 ± 0.02	0.05 ± 0.02	−0.04 ± 0.02	−0.12 ± 0.02	−0.15 ± 0.02	−0.13 ± 0.02	−0.17 ± 0.02	−0.15 ± 0.02	−0.12 ± 0.02	−0.17 ± 0.02	−0.16 ± 0.02	−0.16 ± 0.02
Bacteria	*Psychromonas ingrahamii*	0.29 ± 0.01	0.18 ± 0.02	−0.01 ± 0.02	−0.10 ± 0.02	−0.10 ± 0.02	−0.07 ± 0.02	−0.06 ± 0.02	−0.10 ± 0.02	−0.07 ± 0.02	−0.06 ± 0.02	−0.10 ± 0.02	−0.06 ± 0.02	−0.07 ± 0.02
Bacteria	*Ralstonia solanacearum*	0.76 ± 0.02	0.58 ± 0.02	0.30 ± 0.02	0.07 ± 0.02	0.03 ± 0.02	−0.04 ± 0.02	−0.04 ± 0.02	−0.03 ± 0.02	−0.05 ± 0.02	−0.09 ± 0.02	−0.07 ± 0.02	−0.09 ± 0.02	−0.08 ± 0.02
Bacteria	*Renibacterium salmoninarum*	0.12 ± 0.02	0.09 ± 0.02	−0.03 ± 0.02	−0.16 ± 0.02	−0.12 ± 0.02	−0.11 ± 0.02	−0.13 ± 0.02	−0.12 ± 0.02	−0.14 ± 0.02	−0.13 ± 0.02	−0.16 ± 0.02	−0.17 ± 0.02	−0.13 ± 0.02
Bacteria	*Rhizobium etli*	0.50 ± 0.02	0.36 ± 0.02	0.15 ± 0.02	0.04 ± 0.02	−0.01 ± 0.02	−0.05 ± 0.02	−0.04 ± 0.02	−0.05 ± 0.02	−0.10 ± 0.02	−0.10 ± 0.02	−0.09 ± 0.02	−0.09 ± 0.02	−0.10 ± 0.02
Bacteria	*Rhodobacter sphaeroides*	0.69 ± 0.02	0.47 ± 0.02	0.18 ± 0.02	0.08 ± 0.02	0.02 ± 0.02	0.00 ± 0.02	−0.05 ± 0.02	−0.08 ± 0.02	−0.10 ± 0.02	−0.09 ± 0.02	−0.10 ± 0.02	−0.07 ± 0.02	−0.10 ± 0.02
Bacteria	*Rhodococcus erythropolis*	0.35 ± 0.01	0.28 ± 0.01	0.06 ± 0.01	−0.04 ± 0.01	−0.06 ± 0.01	−0.04 ± 0.01	−0.07 ± 0.01	−0.08 ± 0.01	−0.08 ± 0.01	−0.09 ± 0.01	−0.12 ± 0.01	−0.11 ± 0.01	−0.13 ± 0.01
Bacteria	*Rhodoferax ferrireducens*	0.65 ± 0.01	0.50 ± 0.02	0.13 ± 0.02	−0.02 ± 0.02	−0.05 ± 0.02	−0.06 ± 0.02	−0.11 ± 0.02	−0.10 ± 0.02	−0.11 ± 0.02	−0.13 ± 0.02	−0.16 ± 0.02	−0.16 ± 0.02	−0.10 ± 0.02
Bacteria	*Rhodopirellula baltica*	0.14 ± 0.01	0.16 ± 0.01	0.06 ± 0.01	−0.02 ± 0.01	−0.05 ± 0.01	−0.06 ± 0.01	−0.07 ± 0.01	−0.10 ± 0.01	−0.08 ± 0.01	−0.06 ± 0.01	−0.11 ± 0.01	−0.12 ± 0.01	−0.16 ± 0.01
Bacteria	*Rhodopseudomonas palustris*	0.61 ± 0.02	0.54 ± 0.02	0.24 ± 0.02	0.02 ± 0.02	−0.04 ± 0.02	−0.10 ± 0.02	−0.12 ± 0.02	−0.13 ± 0.02	−0.13 ± 0.01	−0.12 ± 0.02	−0.13 ± 0.01	−0.16 ± 0.02	−0.16 ± 0.02
Bacteria	*Rhodospirillum centenum*	0.64 ± 0.02	0.53 ± 0.02	0.26 ± 0.02	0.09 ± 0.02	−0.02 ± 0.02	−0.03 ± 0.02	−0.04 ± 0.02	−0.08 ± 0.02	−0.07 ± 0.02	−0.07 ± 0.02	−0.08 ± 0.02	−0.07 ± 0.02	−0.08 ± 0.02
Bacteria	*Rickettsia akari*	0.10 ± 0.03	0.06 ± 0.03	−0.10 ± 0.03	−0.10 ± 0.03	−0.09 ± 0.03	−0.18 ± 0.03	−0.09 ± 0.03	−0.09 ± 0.03	−0.04 ± 0.03	−0.03 ± 0.03	−0.09 ± 0.03	−0.07 ± 0.03	−0.11 ± 0.03
Bacteria	*Roseiflexus castenholzii*	0.43 ± 0.01	0.35 ± 0.02	0.12 ± 0.02	−0.02 ± 0.02	−0.04 ± 0.02	−0.05 ± 0.02	−0.04 ± 0.02	−0.06 ± 0.02	−0.07 ± 0.02	−0.05 ± 0.02	−0.03 ± 0.02	−0.05 ± 0.02	−0.04 ± 0.02
Bacteria	*Roseobacter denitrificans*	0.53 ± 0.02	0.37 ± 0.02	0.11 ± 0.02	−0.05 ± 0.02	−0.02 ± 0.02	−0.05 ± 0.02	−0.08 ± 0.02	−0.07 ± 0.02	−0.06 ± 0.02	−0.09 ± 0.02	−0.06 ± 0.02	−0.04 ± 0.02	−0.04 ± 0.02
Bacteria	*Rubrobacter xylanophilus*	0.50 ± 0.02	0.39 ± 0.02	0.18 ± 0.02	−0.01 ± 0.02	−0.09 ± 0.02	−0.06 ± 0.02	−0.08 ± 0.02	−0.05 ± 0.02	−0.05 ± 0.02	−0.08 ± 0.02	−0.06 ± 0.02	−0.05 ± 0.02	−0.06 ± 0.02
Bacteria	*Ruegeria pomeroyi*	0.65 ± 0.02	0.51 ± 0.02	0.19 ± 0.02	0.04 ± 0.02	−0.01 ± 0.02	−0.02 ± 0.02	−0.02 ± 0.02	−0.07 ± 0.02	−0.07 ± 0.02	−0.09 ± 0.02	−0.11 ± 0.02	−0.10 ± 0.02	−0.12 ± 0.02
Bacteria	*Saccharophagus degradans*	0.24 ± 0.02	0.14 ± 0.02	−0.06 ± 0.02	−0.17 ± 0.02	−0.22 ± 0.02	−0.18 ± 0.02	−0.17 ± 0.02	−0.17 ± 0.02	−0.16 ± 0.02	−0.18 ± 0.02	−0.16 ± 0.02	−0.17 ± 0.02	−0.18 ± 0.02
Bacteria	*Saccharopolyspora erythraea*	0.40 ± 0.01	0.25 ± 0.01	0.07 ± 0.01	0.00 ± 0.01	−0.05 ± 0.01	−0.04 ± 0.01	−0.07 ± 0.01	−0.08 ± 0.01	−0.10 ± 0.01	−0.08 ± 0.01	−0.09 ± 0.01	−0.10 ± 0.01	−0.12 ± 0.01
Bacteria	*Salinibacter ruber*	0.59 ± 0.02	0.57 ± 0.02	0.38 ± 0.02	0.23 ± 0.02	0.17 ± 0.02	0.17 ± 0.02	0.15 ± 0.02	0.11 ± 0.02	0.10 ± 0.02	0.06 ± 0.02	0.06 ± 0.02	0.06 ± 0.02	0.04 ± 0.02
Bacteria	*Salinispora arenicola*	0.35 ± 0.01	0.24 ± 0.02	0.05 ± 0.02	−0.01 ± 0.02	−0.02 ± 0.02	−0.02 ± 0.01	−0.01 ± 0.01	−0.02 ± 0.01	−0.08 ± 0.01	−0.05 ± 0.01	−0.06 ± 0.01	−0.06 ± 0.01	−0.10 ± 0.01
Bacteria	*Salmonella enterica*	0.30 ± 0.02	0.21 ± 0.02	0.01 ± 0.02	−0.05 ± 0.02	−0.03 ± 0.02	−0.06 ± 0.02	−0.02 ± 0.02	−0.04 ± 0.02	−0.06 ± 0.02	−0.05 ± 0.02	−0.05 ± 0.02	−0.10 ± 0.02	−0.09 ± 0.02
Bacteria	*Serratia proteamaculans*	0.51 ± 0.01	0.37 ± 0.02	0.10 ± 0.02	0.02 ± 0.02	−0.04 ± 0.02	−0.04 ± 0.02	−0.06 ± 0.02	−0.04 ± 0.02	−0.08 ± 0.02	−0.10 ± 0.02	−0.09 ± 0.02	−0.13 ± 0.02	−0.12 ± 0.02
Bacteria	*Shewanella amazonensis*	0.40 ± 0.02	0.19 ± 0.02	−0.04 ± 0.02	−0.09 ± 0.02	−0.13 ± 0.02	−0.11 ± 0.02	−0.10 ± 0.02	−0.12 ± 0.02	−0.13 ± 0.02	−0.14 ± 0.02	−0.14 ± 0.02	−0.14 ± 0.02	−0.14 ± 0.02
Bacteria	*Shigella boydii*	0.27 ± 0.02	0.25 ± 0.02	−0.02 ± 0.02	−0.12 ± 0.02	−0.07 ± 0.02	0.00 ± 0.02	−0.09 ± 0.02	−0.10 ± 0.02	−0.06 ± 0.02	−0.10 ± 0.02	−0.02 ± 0.02	−0.08 ± 0.02	−0.07 ± 0.02
Bacteria	*Sinorhizobium medicae*	0.52 ± 0.02	0.36 ± 0.02	0.07 ± 0.02	−0.04 ± 0.02	−0.07 ± 0.02	−0.07 ± 0.02	−0.08 ± 0.02	−0.10 ± 0.02	−0.10 ± 0.02	−0.08 ± 0.02	−0.06 ± 0.02	−0.09 ± 0.02	−0.09 ± 0.02
Bacteria	*Sodalis glossinidius*	0.44 ± 0.02	0.29 ± 0.02	0.02 ± 0.02	−0.03 ± 0.02	−0.08 ± 0.02	−0.03 ± 0.02	−0.05 ± 0.02	−0.03 ± 0.02	−0.01 ± 0.02	−0.04 ± 0.02	−0.08 ± 0.02	−0.09 ± 0.02	−0.05 ± 0.02
Bacteria	*Solibacter usitatus*	0.43 ± 0.01	0.36 ± 0.01	0.15 ± 0.01	0.00 ± 0.01	−0.01 ± 0.01	−0.01 ± 0.01	−0.02 ± 0.01	−0.05 ± 0.01	−0.04 ± 0.01	−0.05 ± 0.01	−0.06 ± 0.01	−0.06 ± 0.01	−0.05 ± 0.01
Bacteria	*Sorangium cellulosum*	0.62 ± 0.01	0.59 ± 0.01	0.36 ± 0.01	0.19 ± 0.01	0.12 ± 0.01	0.05 ± 0.01	0.01 ± 0.01	0.00 ± 0.01	−0.01 ± 0.01	−0.04 ± 0.01	−0.03 ± 0.01	−0.04 ± 0.01	−0.05 ± 0.01

Continued on next page

Type	Species	Window 1	Window 2	Window 3	Window 4	Window 5	Window 6	Window 7	Window 8	Window 9	Window 10	Window 11	Window 12	Window 13
Bacteria	*Sphingomonas wittichii*	0.68 ± 0.02	0.50 ± 0.02	0.20 ± 0.02	0.04 ± 0.02	0.02 ± 0.02	−0.03 ± 0.02	−0.04 ± 0.02	−0.05 ± 0.02	−0.06 ± 0.02	−0.09 ± 0.01	−0.08 ± 0.02	−0.09 ± 0.02	−0.09 ± 0.02
Bacteria	*Sphingopyxis alaskensis*	0.59 ± 0.02	0.43 ± 0.02	0.16 ± 0.02	0.05 ± 0.02	−0.02 ± 0.02	−0.01 ± 0.02	−0.04 ± 0.02	−0.01 ± 0.02	−0.06 ± 0.02	−0.09 ± 0.02	−0.08 ± 0.02	−0.10 ± 0.02	−0.09 ± 0.02
Bacteria	*Staphylococcus aureus*	0.04 ± 0.02	0.01 ± 0.02	−0.09 ± 0.02	−0.07 ± 0.02	−0.08 ± 0.02	−0.10 ± 0.02	−0.07 ± 0.02	−0.07 ± 0.02	−0.09 ± 0.02	−0.06 ± 0.02	−0.05 ± 0.02	−0.08 ± 0.02	−0.09 ± 0.02
Bacteria	*Stenotrophomonas maltophilia*	0.72 ± 0.02	0.55 ± 0.02	0.21 ± 0.02	0.05 ± 0.02	0.00 ± 0.02	−0.06 ± 0.02	−0.06 ± 0.02	−0.06 ± 0.02	−0.07 ± 0.02	−0.10 ± 0.02	−0.10 ± 0.02	−0.12 ± 0.02	−0.10 ± 0.02
Bacteria	*Streptococcus agalactiae*	0.20 ± 0.02	−0.01 ± 0.02	−0.14 ± 0.02	−0.15 ± 0.02	−0.14 ± 0.02	−0.12 ± 0.02	−0.14 ± 0.02	−0.06 ± 0.02	−0.09 ± 0.02	−0.13 ± 0.02	−0.19 ± 0.02	−0.09 ± 0.02	−0.07 ± 0.02
Bacteria	*Streptomyces avermitilis*	0.51 ± 0.01	0.43 ± 0.01	0.25 ± 0.01	0.14 ± 0.01	0.11 ± 0.01	0.06 ± 0.01	0.02 ± 0.01	0.05 ± 0.01	0.00 ± 0.01	−0.01 ± 0.01	−0.03 ± 0.01	−0.04 ± 0.01	−0.04 ± 0.01
Bacteria	*Sulfurihydrogenibium azorense*	−0.01 ± 0.03	−0.03 ± 0.03	−0.02 ± 0.03	−0.02 ± 0.03	−0.08 ± 0.03	−0.10 ± 0.03	−0.11 ± 0.03	−0.05 ± 0.03	−0.07 ± 0.03	−0.05 ± 0.03	−0.02 ± 0.02	−0.05 ± 0.03	−0.05 ± 0.03
Bacteria	*Sulfurimonas denitrificans*	0.19 ± 0.02	0.07 ± 0.02	−0.08 ± 0.02	−0.13 ± 0.03	−0.18 ± 0.03	−0.17 ± 0.02	−0.14 ± 0.02	−0.08 ± 0.02	−0.10 ± 0.02	−0.18 ± 0.02	−0.15 ± 0.02	−0.13 ± 0.02	−0.11 ± 0.02
Bacteria	*Sulfurovum sp. NBC37-1*	0.40 ± 0.02	0.25 ± 0.02	−0.02 ± 0.02	−0.07 ± 0.02	−0.09 ± 0.02	−0.10 ± 0.02	−0.10 ± 0.02	−0.06 ± 0.02	−0.10 ± 0.02	−0.11 ± 0.02	−0.14 ± 0.02	−0.12 ± 0.02	−0.13 ± 0.02
Bacteria	*Symbiobacterium thermophilum*	0.43 ± 0.02	0.33 ± 0.02	0.17 ± 0.02	0.10 ± 0.02	0.05 ± 0.02	0.05 ± 0.02	0.02 ± 0.02	−0.02 ± 0.02	−0.03 ± 0.02	0.00 ± 0.02	0.00 ± 0.02	−0.03 ± 0.02	−0.04 ± 0.02
Bacteria	*Synechococcus elongatus*	0.31 ± 0.02	0.25 ± 0.02	0.00 ± 0.02	−0.08 ± 0.02	−0.12 ± 0.02	−0.08 ± 0.02	−0.08 ± 0.02	−0.10 ± 0.02	−0.10 ± 0.02	−0.12 ± 0.02	−0.10 ± 0.02	−0.13 ± 0.02	−0.11 ± 0.02
Bacteria	*Synechocystis sp. PCC 6803*	0.36 ± 0.02	0.29 ± 0.02	0.12 ± 0.02	−0.02 ± 0.02	−0.09 ± 0.02	−0.12 ± 0.02	−0.12 ± 0.02	−0.14 ± 0.02	−0.10 ± 0.02	−0.07 ± 0.02	−0.05 ± 0.02	−0.08 ± 0.02	−0.07 ± 0.02
Bacteria	*Syntrophobacter fumaroxidans*	0.39 ± 0.02	0.36 ± 0.02	0.17 ± 0.02	0.02 ± 0.02	−0.03 ± 0.02	−0.02 ± 0.02	−0.03 ± 0.02	−0.03 ± 0.02	−0.05 ± 0.02	−0.06 ± 0.02	−0.07 ± 0.02	−0.05 ± 0.02	−0.07 ± 0.02
Bacteria	*Syntrophomonas wolfei*	0.18 ± 0.02	0.12 ± 0.02	0.00 ± 0.02	−0.02 ± 0.02	−0.09 ± 0.02	−0.12 ± 0.02	−0.12 ± 0.02	−0.08 ± 0.02	−0.13 ± 0.02	−0.13 ± 0.02	−0.12 ± 0.02	−0.12 ± 0.02	−0.09 ± 0.02
Bacteria	*Syntrophus aciditrophicus*	0.29 ± 0.02	0.26 ± 0.02	0.16 ± 0.02	0.07 ± 0.02	−0.01 ± 0.02	−0.02 ± 0.02	−0.03 ± 0.02	−0.01 ± 0.02	−0.07 ± 0.02	−0.02 ± 0.02	−0.07 ± 0.02	−0.07 ± 0.02	−0.05 ± 0.02
Bacteria	*Thauera sp. MZ1T*	0.76 ± 0.02	0.57 ± 0.02	0.20 ± 0.02	0.06 ± 0.02	0.03 ± 0.02	−0.02 ± 0.02	−0.04 ± 0.02	−0.06 ± 0.02	−0.04 ± 0.02	−0.10 ± 0.02	−0.11 ± 0.02	−0.13 ± 0.02	−0.11 ± 0.02
Bacteria	*Thermoanaerobacter pseudethanolicus*	−0.01 ± 0.02	−0.02 ± 0.02	−0.04 ± 0.02	−0.05 ± 0.02	−0.10 ± 0.02	−0.09 ± 0.02	−0.05 ± 0.02	−0.06 ± 0.02	−0.07 ± 0.02	−0.06 ± 0.02	0.00 ± 0.02	−0.01 ± 0.02	0.01 ± 0.02
Bacteria	*Thermobifida fusca*	0.36 ± 0.02	0.28 ± 0.02	0.15 ± 0.02	0.10 ± 0.02	0.07 ± 0.02	0.07 ± 0.02	0.01 ± 0.02	0.01 ± 0.02	0.01 ± 0.02	−0.04 ± 0.02	−0.01 ± 0.02	0.00 ± 0.02	−0.02 ± 0.02
Bacteria	*Thermodesulfovibrio yellowstonii*	0.03 ± 0.02	0.02 ± 0.02	−0.04 ± 0.02	−0.09 ± 0.02	−0.14 ± 0.02	−0.14 ± 0.02	−0.12 ± 0.02	−0.06 ± 0.02	−0.07 ± 0.02	−0.06 ± 0.02	−0.05 ± 0.02	−0.09 ± 0.02	−0.05 ± 0.02
Bacteria	*Thermomicrobium roseum*	0.15 ± 0.02	0.16 ± 0.02	0.06 ± 0.02	0.02 ± 0.02	−0.05 ± 0.02	−0.08 ± 0.02	−0.08 ± 0.02	−0.08 ± 0.02	−0.05 ± 0.02	−0.08 ± 0.02	−0.10 ± 0.02	−0.05 ± 0.02	−0.08 ± 0.02
Bacteria	*Thermosipho africanus*	−0.09 ± 0.02	0.02 ± 0.02	−0.05 ± 0.03	−0.07 ± 0.03	−0.05 ± 0.02	−0.03 ± 0.02	−0.08 ± 0.02	−0.08 ± 0.02	−0.08 ± 0.02	−0.05 ± 0.02	−0.06 ± 0.02	−0.10 ± 0.02	−0.08 ± 0.02
Bacteria	*Thermosynechococcus elongatus*	0.30 ± 0.02	0.27 ± 0.02	0.15 ± 0.02	0.00 ± 0.02	−0.07 ± 0.02	−0.07 ± 0.02	0.01 ± 0.02	−0.09 ± 0.02	−0.03 ± 0.02	−0.09 ± 0.02	−0.04 ± 0.02	−0.11 ± 0.02	−0.04 ± 0.02
Bacteria	*Thermotoga lettingae*	−0.05 ± 0.02	−0.05 ± 0.02	−0.02 ± 0.02	−0.04 ± 0.02	−0.02 ± 0.02	−0.06 ± 0.02	−0.05 ± 0.02	−0.08 ± 0.02	−0.08 ± 0.02	−0.10 ± 0.02	−0.03 ± 0.02	−0.02 ± 0.02	−0.06 ± 0.02
Bacteria	*Thermus thermophilus*	0.31 ± 0.02	0.30 ± 0.02	0.10 ± 0.03	−0.10 ± 0.02	−0.16 ± 0.02	−0.18 ± 0.02	−0.11 ± 0.02	−0.15 ± 0.02	−0.19 ± 0.02	−0.17 ± 0.02	−0.17 ± 0.02	−0.19 ± 0.02	−0.15 ± 0.02
Bacteria	*Thioalkalivibrio sp. HL-EbGR7*	0.65 ± 0.02	0.48 ± 0.02	0.17 ± 0.02	−0.08 ± 0.02	−0.13 ± 0.02	−0.13 ± 0.02	−0.12 ± 0.02	−0.11 ± 0.02	−0.12 ± 0.02	−0.13 ± 0.02	−0.13 ± 0.02	−0.12 ± 0.02	−0.14 ± 0.02
Bacteria	*Thiobacillus denitrificans*	0.60 ± 0.02	0.46 ± 0.02	0.20 ± 0.02	0.00 ± 0.02	−0.01 ± 0.02	−0.03 ± 0.02	−0.04 ± 0.02	−0.09 ± 0.02	−0.08 ± 0.02	−0.10 ± 0.02	−0.08 ± 0.02	−0.11 ± 0.02	−0.09 ± 0.02
Bacteria	*Thiomicrospira crunogena*	0.30 ± 0.02	0.20 ± 0.02	0.02 ± 0.02	−0.15 ± 0.02	−0.16 ± 0.02	−0.18 ± 0.02	−0.10 ± 0.02	−0.12 ± 0.02	−0.14 ± 0.02	−0.15 ± 0.02	−0.13 ± 0.02	−0.14 ± 0.02	−0.12 ± 0.02
Bacteria	*Tolumonas auensis*	0.38 ± 0.02	0.21 ± 0.02	−0.03 ± 0.02	−0.08 ± 0.02	−0.12 ± 0.02	−0.12 ± 0.02	−0.12 ± 0.02	−0.09 ± 0.02	−0.09 ± 0.02	−0.11 ± 0.02	−0.13 ± 0.02	−0.12 ± 0.02	−0.09 ± 0.02
Bacteria	*Treponema denticola*	0.18 ± 0.02	0.05 ± 0.02	−0.12 ± 0.02	−0.14 ± 0.02	−0.13 ± 0.02	−0.18 ± 0.02	−0.15 ± 0.02	−0.18 ± 0.02	−0.15 ± 0.02	−0.17 ± 0.02	−0.16 ± 0.02	−0.16 ± 0.02	−0.12 ± 0.02
Bacteria	*Trichodesmium erythraeum*	0.14 ± 0.01	0.10 ± 0.02	−0.03 ± 0.02	−0.10 ± 0.02	−0.10 ± 0.02	−0.11 ± 0.02	−0.06 ± 0.02	−0.04 ± 0.02	−0.05 ± 0.02	−0.05 ± 0.02	−0.05 ± 0.02	−0.02 ± 0.02	−0.02 ± 0.02
Bacteria	*Tropheryma whipplei*	−0.01 ± 0.04	−0.02 ± 0.04	−0.10 ± 0.04	−0.12 ± 0.04	−0.12 ± 0.04	−0.15 ± 0.04	−0.18 ± 0.04	−0.11 ± 0.03	−0.11 ± 0.04	−0.10 ± 0.03	−0.10 ± 0.04	−0.10 ± 0.04	−0.15 ± 0.04
Bacteria	*Ureaplasma parvum*	−0.10 ± 0.08	−0.08 ± 0.08	−0.15 ± 0.10	−0.21 ± 0.10	−0.17 ± 0.10	−0.06 ± 0.09	−0.21 ± 0.11	−0.05 ± 0.08	−0.12 ± 0.08	−0.04 ± 0.09	−0.03 ± 0.09	−0.09 ± 0.09	0.06 ± 0.07
Bacteria	*Variovorax paradoxus*	0.86 ± 0.01	0.67 ± 0.01	0.25 ± 0.01	0.04 ± 0.01	0.02 ± 0.01	−0.03 ± 0.01	−0.08 ± 0.01	−0.09 ± 0.01	−0.13 ± 0.01	−0.16 ± 0.01	−0.14 ± 0.01	−0.16 ± 0.01	−0.17 ± 0.01
Bacteria	*Verminephrobacter eiseniae*	0.63 ± 0.01	0.49 ± 0.02	0.15 ± 0.02	0.03 ± 0.02	−0.01 ± 0.02	−0.02 ± 0.01	−0.03 ± 0.01	−0.10 ± 0.02	−0.13 ± 0.01	−0.11 ± 0.01	−0.13 ± 0.01	−0.15 ± 0.01	−0.15 ± 0.01
Bacteria	*Vibrio cholerae*	0.25 ± 0.02	0.17 ± 0.02	−0.02 ± 0.02	−0.12 ± 0.02	−0.12 ± 0.02	−0.12 ± 0.02	−0.04 ± 0.02	−0.06 ± 0.02	−0.13 ± 0.02	−0.09 ± 0.02	−0.08 ± 0.02	−0.10 ± 0.02	−0.11 ± 0.02
Bacteria	*Wigglesworthia glossinidia*	−0.11 ± 0.05	−0.06 ± 0.05	−0.13 ± 0.04	−0.14 ± 0.04	−0.20 ± 0.05	−0.12 ± 0.04	−0.13 ± 0.04	−0.12 ± 0.04	−0.15 ± 0.04	−0.12 ± 0.04	−0.05 ± 0.04	−0.07 ± 0.04	−0.02 ± 0.04
Bacteria	*Wolbachia endosymbiont*	0.09 ± 0.03	0.12 ± 0.03	−0.03 ± 0.03	−0.20 ± 0.03	−0.24 ± 0.03	−0.16 ± 0.03	−0.08 ± 0.03	−0.06 ± 0.03	0.05 ± 0.03	0.00 ± 0.03	−0.11 ± 0.03	−0.05 ± 0.03	−0.04 ± 0.03
Bacteria	*Wolinella succinogenes*	0.28 ± 0.02	0.14 ± 0.02	−0.07 ± 0.03	−0.09 ± 0.02	−0.11 ± 0.02	−0.09 ± 0.02	−0.07 ± 0.02	−0.13 ± 0.02	−0.12 ± 0.02	−0.11 ± 0.02	−0.10 ± 0.02	−0.10 ± 0.02	−0.12 ± 0.02
Bacteria	*Xanthobacter autotrophicus*	0.55 ± 0.02	0.44 ± 0.02	0.21 ± 0.02	0.08 ± 0.02	0.02 ± 0.02	0.01 ± 0.02	−0.04 ± 0.02	−0.04 ± 0.02	−0.07 ± 0.02	−0.09 ± 0.02	−0.09 ± 0.02	−0.10 ± 0.02	−0.12 ± 0.02

Continued on next page

Type	Species	Window 1	Window 2	Window 3	Window 4	Window 5	Window 6	Window 7	Window 8	Window 9	Window 10	Window 11	Window 12	Window 13
Bacteria	*Xanthomonas axonopodis*	0.57 ± 0.02	0.50 ± 0.02	0.20 ± 0.02	0.03 ± 0.02	0.01 ± 0.02	-0.01 ± 0.02	-0.04 ± 0.02	-0.09 ± 0.02	-0.12 ± 0.02	-0.12 ± 0.02	-0.14 ± 0.02	-0.12 ± 0.02	-0.13 ± 0.02
Bacteria	*Xylella fastidiosa*	0.15 ± 0.02	0.15 ± 0.02	0.03 ± 0.02	-0.06 ± 0.02	-0.08 ± 0.02	-0.06 ± 0.02	-0.08 ± 0.02	-0.07 ± 0.02	-0.05 ± 0.02	-0.07 ± 0.02	-0.05 ± 0.02	-0.06 ± 0.02	-0.04 ± 0.02
Bacteria	*Yersinia enterocolitica*	0.24 ± 0.01	0.12 ± 0.02	-0.10 ± 0.02	-0.14 ± 0.02	-0.12 ± 0.02	-0.14 ± 0.02	-0.14 ± 0.02	-0.11 ± 0.02	-0.11 ± 0.02	-0.10 ± 0.02	-0.13 ± 0.02	-0.10 ± 0.02	-0.09 ± 0.02
Bacteria	*Zymomonas mobilis*	0.08 ± 0.02	0.04 ± 0.02	-0.12 ± 0.03	-0.17 ± 0.03	-0.17 ± 0.03	-0.21 ± 0.03	-0.12 ± 0.02	-0.16 ± 0.03	-0.19 ± 0.03	-0.21 ± 0.03	-0.18 ± 0.03	-0.18 ± 0.02	-0.15 ± 0.02
Archaea	*Aeropyrum pernix*	0.15 ± 0.02	0.08 ± 0.02	0.01 ± 0.03	0.00 ± 0.03	-0.01 ± 0.03	-0.03 ± 0.02	-0.02 ± 0.02	0.01 ± 0.02	-0.04 ± 0.02	-0.03 ± 0.02	0.02 ± 0.02	0.02 ± 0.02	0.00 ± 0.02
Archaea	*Caldivirga maquilingensis*	-0.05 ± 0.02	0.00 ± 0.02	-0.03 ± 0.02	-0.04 ± 0.02	-0.02 ± 0.02	-0.10 ± 0.02	-0.07 ± 0.02	-0.03 ± 0.02	-0.06 ± 0.02	-0.07 ± 0.02	-0.05 ± 0.02	-0.07 ± 0.02	-0.09 ± 0.02
Archaea	*Desulfurococcus kamchatkensis*	0.03 ± 0.03	0.05 ± 0.03	0.00 ± 0.03	-0.03 ± 0.03	-0.04 ± 0.03	-0.05 ± 0.03	-0.04 ± 0.03	-0.05 ± 0.03	-0.06 ± 0.03	0.02 ± 0.03	-0.02 ± 0.03	0.01 ± 0.03	-0.01 ± 0.03
Archaea	*Haloarcula marismortui*	0.38 ± 0.02	0.22 ± 0.02	0.11 ± 0.02	0.04 ± 0.02	0.02 ± 0.02	-0.02 ± 0.02	-0.02 ± 0.02	-0.05 ± 0.02	-0.03 ± 0.02	-0.04 ± 0.02	-0.04 ± 0.02	-0.06 ± 0.02	-0.05 ± 0.02
Archaea	*Halobacterium salinarum*	0.46 ± 0.02	0.28 ± 0.02	0.11 ± 0.02	0.08 ± 0.02	0.02 ± 0.02	0.00 ± 0.02	0.00 ± 0.02	-0.07 ± 0.02	-0.03 ± 0.02	-0.05 ± 0.02	-0.03 ± 0.02	-0.06 ± 0.02	-0.08 ± 0.02
Archaea	*Haloquadratum walsbyi*	-0.01 ± 0.02	-0.08 ± 0.02	-0.14 ± 0.02	-0.10 ± 0.02	-0.15 ± 0.02	-0.12 ± 0.02	-0.15 ± 0.02	-0.13 ± 0.02	-0.11 ± 0.02	-0.08 ± 0.02	-0.10 ± 0.02	-0.09 ± 0.02	-0.06 ± 0.02
Archaea	*Halorubrum lacusprofundi*	0.42 ± 0.02	0.22 ± 0.02	0.10 ± 0.02	0.03 ± 0.02	-0.01 ± 0.02	-0.09 ± 0.02	-0.06 ± 0.02	-0.09 ± 0.02	-0.09 ± 0.02	-0.07 ± 0.02	-0.09 ± 0.02	-0.10 ± 0.02	-0.10 ± 0.02
Archaea	*Hyperthermus butylicus*	-0.06 ± 0.03	-0.03 ± 0.03	-0.03 ± 0.03	-0.07 ± 0.03	-0.02 ± 0.03	-0.05 ± 0.02	-0.08 ± 0.03	-0.09 ± 0.03	-0.03 ± 0.02	-0.04 ± 0.02	-0.03 ± 0.03	-0.03 ± 0.02	-0.04 ± 0.03
Archaea	*Ignicoccus hospitalis*	0.05 ± 0.03	0.03 ± 0.03	0.03 ± 0.03	-0.06 ± 0.03	-0.05 ± 0.03	-0.03 ± 0.03	-0.02 ± 0.03	-0.03 ± 0.03	-0.03 ± 0.03	-0.05 ± 0.03	-0.06 ± 0.03	-0.01 ± 0.03	-0.02 ± 0.03
Archaea	*Metallosphaera sedula*	0.13 ± 0.02	0.16 ± 0.02	0.05 ± 0.02	0.01 ± 0.02	-0.02 ± 0.02	-0.04 ± 0.02	-0.08 ± 0.02	-0.05 ± 0.02	-0.04 ± 0.02	-0.04 ± 0.02	-0.02 ± 0.02	-0.03 ± 0.02	-0.09 ± 0.02
Archaea	*Methanobrevibacter smithii*	0.15 ± 0.02	0.03 ± 0.02	-0.12 ± 0.03	-0.15 ± 0.03	-0.12 ± 0.03	-0.13 ± 0.03	-0.09 ± 0.02	-0.11 ± 0.02	-0.12 ± 0.03	-0.11 ± 0.03	-0.13 ± 0.03	-0.09 ± 0.03	-0.10 ± 0.02
Archaea	*Methanocaldococcus jannaschii*	-0.05 ± 0.03	-0.01 ± 0.03	0.01 ± 0.02	-0.02 ± 0.02	0.00 ± 0.02	0.01 ± 0.02	0.01 ± 0.02	0.03 ± 0.02	0.01 ± 0.03	0.02 ± 0.02	0.01 ± 0.02	0.01 ± 0.02	0.01 ± 0.02
Archaea	*Methanococcoides burtonii*	0.27 ± 0.02	0.16 ± 0.02	-0.05 ± 0.02	-0.14 ± 0.02	-0.16 ± 0.02	-0.14 ± 0.02	-0.14 ± 0.02	-0.17 ± 0.02	-0.10 ± 0.02	-0.12 ± 0.02	-0.09 ± 0.02	-0.08 ± 0.02	-0.09 ± 0.02
Archaea	*Methanococcus aeolicus*	0.08 ± 0.03	0.06 ± 0.03	-0.08 ± 0.03	-0.14 ± 0.03	-0.08 ± 0.03	-0.06 ± 0.03	-0.07 ± 0.03	-0.11 ± 0.03	-0.13 ± 0.03	-0.10 ± 0.03	-0.12 ± 0.03	-0.13 ± 0.03	-0.07 ± 0.03
Archaea	*Methanocorpusculum labreanum*	0.40 ± 0.02	0.20 ± 0.02	-0.05 ± 0.03	-0.13 ± 0.03	-0.05 ± 0.03	-0.08 ± 0.03	-0.10 ± 0.03	-0.13 ± 0.03	-0.10 ± 0.03	-0.11 ± 0.03	-0.11 ± 0.03	-0.10 ± 0.03	-0.10 ± 0.03
Archaea	*Methanoculleus marisnigri*	0.49 ± 0.02	0.35 ± 0.02	0.14 ± 0.02	0.02 ± 0.02	0.02 ± 0.02	0.01 ± 0.02	0.00 ± 0.02	-0.05 ± 0.02	-0.04 ± 0.02	-0.04 ± 0.02	-0.09 ± 0.02	-0.06 ± 0.02	-0.05 ± 0.02
Archaea	*Methanopyrus kandleri*	0.05 ± 0.02	0.05 ± 0.03	0.06 ± 0.03	0.01 ± 0.02	0.01 ± 0.02	0.01 ± 0.03	-0.01 ± 0.02	-0.02 ± 0.03	0.02 ± 0.02	-0.02 ± 0.02	0.00 ± 0.02	0.03 ± 0.02	-0.02 ± 0.02
Archaea	*Methanosaeta thermophila*	0.21 ± 0.02	0.15 ± 0.02	0.05 ± 0.02	-0.06 ± 0.03	-0.09 ± 0.02	-0.11 ± 0.03	-0.12 ± 0.03	-0.09 ± 0.02	-0.07 ± 0.02	-0.07 ± 0.02	-0.09 ± 0.03	-0.07 ± 0.03	-0.09 ± 0.03
Archaea	*Methanosarcina acetivorans*	0.16 ± 0.01	0.14 ± 0.01	0.04 ± 0.02	-0.01 ± 0.02	-0.02 ± 0.02	-0.07 ± 0.02	-0.09 ± 0.02	-0.07 ± 0.02	-0.08 ± 0.02	-0.04 ± 0.02	-0.06 ± 0.01	-0.08 ± 0.02	-0.05 ± 0.02
Archaea	*Methanosphaera stadtmanae*	0.06 ± 0.03	-0.01 ± 0.03	-0.12 ± 0.03	-0.14 ± 0.03	-0.10 ± 0.03	-0.10 ± 0.03	-0.08 ± 0.03	-0.10 ± 0.03	-0.14 ± 0.03	-0.09 ± 0.03	-0.15 ± 0.03	-0.13 ± 0.03	-0.06 ± 0.02
Archaea	*Methanosphaerula palustris*	0.45 ± 0.02	0.31 ± 0.02	0.07 ± 0.02	0.02 ± 0.02	0.01 ± 0.02	0.03 ± 0.02	-0.05 ± 0.02	-0.01 ± 0.02	-0.02 ± 0.02	-0.02 ± 0.02	-0.07 ± 0.02	-0.10 ± 0.02	-0.07 ± 0.02
Archaea	*Methanospirillum hungatei*	0.15 ± 0.02	0.04 ± 0.02	-0.05 ± 0.02	-0.10 ± 0.02	-0.08 ± 0.02	-0.08 ± 0.02	-0.06 ± 0.02	-0.05 ± 0.02	-0.05 ± 0.02	-0.03 ± 0.02	-0.04 ± 0.02	-0.05 ± 0.02	-0.07 ± 0.02
Archaea	*Methanothermobacter thermautotrophicus*	0.28 ± 0.02	0.20 ± 0.02	0.10 ± 0.02	-0.01 ± 0.02	-0.08 ± 0.03	-0.12 ± 0.02	-0.11 ± 0.02	-0.05 ± 0.02	-0.09 ± 0.02	-0.13 ± 0.02	-0.17 ± 0.02	-0.10 ± 0.02	-0.11 ± 0.02
Archaea	*Nanoarchaeum equitans*	-0.24 ± 0.05	-0.08 ± 0.05	-0.07 ± 0.05	-0.03 ± 0.05	-0.08 ± 0.04	-0.03 ± 0.04	-0.03 ± 0.04	-0.06 ± 0.05	-0.06 ± 0.04	-0.06 ± 0.04	-0.08 ± 0.05	-0.10 ± 0.04	-0.04 ± 0.04
Archaea	*Natronomonas pharaonis*	0.31 ± 0.02	0.11 ± 0.02	-0.03 ± 0.02	-0.10 ± 0.02	-0.12 ± 0.02	-0.13 ± 0.02	-0.13 ± 0.02	-0.13 ± 0.02	-0.10 ± 0.02	-0.08 ± 0.02	-0.12 ± 0.02	-0.11 ± 0.02	-0.10 ± 0.02
Archaea	*Nitrosopumilus maritimus*	-0.04 ± 0.02	-0.08 ± 0.03	-0.23 ± 0.03	-0.24 ± 0.03	-0.16 ± 0.03	-0.16 ± 0.03	-0.10 ± 0.02	-0.12 ± 0.02	-0.08 ± 0.03	-0.12 ± 0.02	-0.09 ± 0.02	-0.06 ± 0.02	-0.08 ± 0.02
Archaea	*Picrophilus torridus*	0.01 ± 0.03	-0.03 ± 0.03	-0.08 ± 0.03	-0.13 ± 0.03	-0.13 ± 0.03	-0.16 ± 0.03	-0.13 ± 0.03	-0.12 ± 0.03	-0.14 ± 0.03	-0.10 ± 0.03	-0.12 ± 0.03	-0.11 ± 0.03	-0.12 ± 0.03
Archaea	*Pyrobaculum aerophilum*	0.06 ± 0.02	0.06 ± 0.02	-0.02 ± 0.02	-0.03 ± 0.02	-0.01 ± 0.02	-0.01 ± 0.02	0.01 ± 0.02	-0.04 ± 0.02	-0.04 ± 0.02	-0.05 ± 0.02	-0.03 ± 0.02	-0.04 ± 0.02	-0.05 ± 0.02
Archaea	*Pyrococcus abyssi*	0.00 ± 0.02	0.00 ± 0.02	-0.04 ± 0.02	-0.04 ± 0.02	-0.02 ± 0.02	0.00 ± 0.02	-0.03 ± 0.02	-0.07 ± 0.02	-0.02 ± 0.02	-0.05 ± 0.02	-0.01 ± 0.02	-0.02 ± 0.02	-0.02 ± 0.02
Archaea	*Staphylothermus marinus*	-0.08 ± 0.03	-0.01 ± 0.03	-0.02 ± 0.03	-0.06 ± 0.03	-0.06 ± 0.03	-0.03 ± 0.03	-0.03 ± 0.02	-0.01 ± 0.03	-0.05 ± 0.03	0.03 ± 0.02	0.01 ± 0.03	-0.06 ± 0.03	-0.02 ± 0.03
Archaea	*Sulfolobus acidocaldarius*	0.02 ± 0.02	0.04 ± 0.02	0.06 ± 0.02	0.00 ± 0.02	0.04 ± 0.02	-0.02 ± 0.02	0.02 ± 0.02	0.02 ± 0.02	0.00 ± 0.02	-0.01 ± 0.02	0.00 ± 0.02	-0.02 ± 0.02	-0.02 ± 0.02
Archaea	*Thermococcus gammatolerans*	0.14 ± 0.02	0.09 ± 0.02	0.03 ± 0.02	-0.03 ± 0.02	-0.07 ± 0.02	-0.07 ± 0.02	-0.05 ± 0.02	-0.01 ± 0.02	-0.01 ± 0.02	-0.02 ± 0.02	-0.04 ± 0.02	0.00 ± 0.02	0.01 ± 0.02
Archaea	*Thermofilum pendens*	0.18 ± 0.02	0.18 ± 0.02	0.06 ± 0.02	0.03 ± 0.02	-0.01 ± 0.02	0.04 ± 0.02	0.01 ± 0.02	-0.02 ± 0.02	0.00 ± 0.02	0.02 ± 0.02	-0.01 ± 0.02	-0.01 ± 0.02	0.00 ± 0.02
Archaea	*Thermoplasma acidophilum*	0.11 ± 0.03	0.06 ± 0.03	-0.05 ± 0.03	-0.03 ± 0.03	-0.05 ± 0.03	-0.02 ± 0.03	-0.03 ± 0.03	-0.04 ± 0.03	-0.10 ± 0.03	-0.05 ± 0.03	-0.06 ± 0.03	-0.05 ± 0.03	-0.07 ± 0.03
Archaea	*Thermoproteus neutrophilus*	0.08 ± 0.02	0.09 ± 0.02	0.05 ± 0.02	0.04 ± 0.02	0.02 ± 0.02	0.02 ± 0.02	0.02 ± 0.02	-0.03 ± 0.02	-0.05 ± 0.02	0.00 ± 0.02	-0.01 ± 0.02	0.00 ± 0.02	0.01 ± 0.02

Continued on next page

Type	Species	Window 1	Window 2	Window 3	Window 4	Window 5	Window 6	Window 7	Window 8	Window 9	Window 10	Window 11	Window 12	Window 13
Fungi	*Aspergillus fumigatus*	0.20 ± 0.01	0.06 ± 0.01	-0.11 ± 0.01	-0.07 ± 0.01	0.03 ± 0.01	0.10 ± 0.01	0.13 ± 0.01	0.15 ± 0.01	0.12 ± 0.01	0.10 ± 0.01	0.13 ± 0.01	0.14 ± 0.01	0.12 ± 0.01
Fungi	*Candida dubliniensis*	0.17 ± 0.01	0.01 ± 0.01	-0.21 ± 0.02	-0.17 ± 0.01	-0.12 ± 0.01	-0.10 ± 0.01	-0.08 ± 0.01	-0.08 ± 0.01	-0.04 ± 0.01	-0.02 ± 0.01	-0.01 ± 0.01	-0.02 ± 0.01	-0.02 ± 0.01
Fungi	*Cryptococcus neoformans*	0.21 ± 0.01	0.11 ± 0.01	-0.03 ± 0.01	-0.04 ± 0.01	0.01 ± 0.01	0.06 ± 0.01	0.06 ± 0.01	0.07 ± 0.01	0.09 ± 0.01	0.06 ± 0.01	0.08 ± 0.01	0.07 ± 0.01	0.07 ± 0.01
Fungi	*Debaryomyces hansenii*	0.13 ± 0.01	-0.04 ± 0.01	-0.25 ± 0.01	-0.23 ± 0.01	-0.19 ± 0.01	-0.13 ± 0.01	-0.10 ± 0.01	-0.08 ± 0.01	-0.05 ± 0.01	-0.02 ± 0.01	-0.04 ± 0.01	-0.04 ± 0.01	-0.01 ± 0.01
Fungi	*Encephalitozoon cuniculi*	0.15 ± 0.02	0.13 ± 0.02	0.08 ± 0.02	0.00 ± 0.02	0.00 ± 0.02	-0.08 ± 0.02	-0.07 ± 0.02	-0.05 ± 0.02	-0.07 ± 0.02	-0.03 ± 0.02	-0.05 ± 0.02	-0.02 ± 0.02	-0.01 ± 0.02
Fungi	*Eremothecium gossypii*	0.40 ± 0.01	0.24 ± 0.02	0.04 ± 0.02	-0.09 ± 0.02	-0.10 ± 0.02	-0.09 ± 0.02	-0.05 ± 0.02	-0.07 ± 0.02	-0.08 ± 0.02	-0.08 ± 0.02	-0.09 ± 0.02	-0.09 ± 0.02	-0.10 ± 0.02
Fungi	*Kluyveromyces lactis*	0.11 ± 0.01	-0.14 ± 0.02	-0.33 ± 0.02	-0.33 ± 0.02	-0.25 ± 0.02	-0.19 ± 0.02	-0.13 ± 0.01	-0.11 ± 0.01	-0.08 ± 0.01	-0.10 ± 0.01	-0.08 ± 0.01	-0.09 ± 0.01	-0.11 ± 0.01
Fungi	*Pichia stipitis*	0.07 ± 0.01	-0.18 ± 0.02	-0.45 ± 0.02	-0.41 ± 0.02	-0.34 ± 0.02	-0.28 ± 0.02	-0.21 ± 0.02	-0.13 ± 0.01	-0.10 ± 0.01	-0.09 ± 0.01	-0.05 ± 0.01	-0.06 ± 0.01	-0.07 ± 0.01
Fungi	*Saccharomyces cerevisiae*	0.14 ± 0.01	0.00 ± 0.01	-0.17 ± 0.01	-0.18 ± 0.01	-0.18 ± 0.01	-0.12 ± 0.01	-0.09 ± 0.01	-0.09 ± 0.01	-0.08 ± 0.01	-0.06 ± 0.01	-0.06 ± 0.01	-0.07 ± 0.01	-0.09 ± 0.01
Fungi	*Schizosaccharomyces pombe*	0.00 ± 0.01	-0.06 ± 0.02	-0.19 ± 0.02	-0.21 ± 0.02	-0.18 ± 0.02	-0.15 ± 0.02	-0.13 ± 0.02	-0.08 ± 0.01	-0.08 ± 0.01	-0.08 ± 0.01	-0.06 ± 0.01	-0.06 ± 0.01	-0.02 ± 0.01
Fungi	*Yarrowia lipolytica*	0.29 ± 0.01	0.06 ± 0.01	-0.26 ± 0.01	-0.35 ± 0.01	-0.28 ± 0.01	-0.22 ± 0.01	-0.20 ± 0.01	-0.13 ± 0.01	-0.06 ± 0.01	-0.09 ± 0.01	-0.10 ± 0.01	-0.08 ± 0.01	-0.07 ± 0.01
Plant	*Arabidopsis thaliana*	0.03 ± 0.01	0.01 ± 0.01	-0.05 ± 0.01	-0.05 ± 0.01	-0.02 ± 0.01	-0.01 ± 0.01	0.01 ± 0.01	0.01 ± 0.01	0.00 ± 0.01	0.01 ± 0.01	0.01 ± 0.01	0.00 ± 0.01	0.00 ± 0.01
Plant	*Oryza sativa*	-0.09 ± 0.01	-0.15 ± 0.01	-0.23 ± 0.01	-0.25 ± 0.01	-0.24 ± 0.01	-0.24 ± 0.01	-0.22 ± 0.01	-0.21 ± 0.01	-0.21 ± 0.01	-0.19 ± 0.01	-0.20 ± 0.01	-0.19 ± 0.01	-0.19 ± 0.01
Insect	*Anopheles gambiae*	0.35 ± 0.01	0.23 ± 0.01	0.05 ± 0.01	0.00 ± 0.01	0.03 ± 0.01	0.08 ± 0.01	0.09 ± 0.01	0.07 ± 0.01	0.06 ± 0.01	0.05 ± 0.01	0.05 ± 0.01	0.04 ± 0.01	0.02 ± 0.01
Insect	*Drosophila melanogaster*	0.19 ± 0.01	0.07 ± 0.01	-0.13 ± 0.01	-0.16 ± 0.01	-0.10 ± 0.01	-0.06 ± 0.01	-0.08 ± 0.01	-0.08 ± 0.01	-0.07 ± 0.01	-0.07 ± 0.01	-0.07 ± 0.01	-0.06 ± 0.01	-0.08 ± 0.01
Fish	*Danio rerio*	0.06 ± 0.01	0.02 ± 0.01	-0.11 ± 0.01	-0.11 ± 0.01	-0.05 ± 0.01	-0.02 ± 0.01	-0.02 ± 0.01	-0.03 ± 0.01	0.00 ± 0.01	-0.03 ± 0.01	-0.02 ± 0.01	-0.01 ± 0.01	-0.02 ± 0.01
Fish	*Gasterosteus aculeatus*	0.13 ± 0.01	0.06 ± 0.01	-0.03 ± 0.01	-0.06 ± 0.01	-0.03 ± 0.01	-0.02 ± 0.01	-0.02 ± 0.01	-0.01 ± 0.01	-0.01 ± 0.01	-0.02 ± 0.01	-0.03 ± 0.01	-0.05 ± 0.01	-0.05 ± 0.01
Fish	*Oryzias latipes*	0.06 ± 0.01	0.01 ± 0.01	-0.10 ± 0.01	-0.10 ± 0.01	-0.06 ± 0.01	-0.03 ± 0.01	-0.03 ± 0.01	-0.06 ± 0.01	-0.07 ± 0.01	-0.07 ± 0.01	-0.06 ± 0.01	-0.07 ± 0.01	-0.06 ± 0.01
Fish	*Takifugu rubripes*	0.13 ± 0.01	0.07 ± 0.01	-0.07 ± 0.01	-0.07 ± 0.01	-0.01 ± 0.01	0.02 ± 0.01	0.01 ± 0.01	0.00 ± 0.01	-0.01 ± 0.01	0.00 ± 0.01	0.00 ± 0.01	-0.02 ± 0.01	-0.03 ± 0.01
Bird	*Gallus gallus*	-0.28 ± 0.01	-0.34 ± 0.01	-0.39 ± 0.01	-0.36 ± 0.01	-0.33 ± 0.01	-0.32 ± 0.01	-0.27 ± 0.01	-0.25 ± 0.01	-0.22 ± 0.01	-0.21 ± 0.01	-0.23 ± 0.01	-0.20 ± 0.01	-0.17 ± 0.01
Bird	*Taeniopygia guttata*	-0.23 ± 0.02	-0.28 ± 0.02	-0.35 ± 0.02	-0.33 ± 0.02	-0.29 ± 0.02	-0.25 ± 0.02	-0.24 ± 0.01	-0.19 ± 0.01	-0.18 ± 0.01	-0.15 ± 0.01	-0.15 ± 0.01	-0.17 ± 0.01	-0.14 ± 0.01
Mammal	*Bos taurus*	-0.11 ± 0.01	-0.19 ± 0.01	-0.26 ± 0.01	-0.25 ± 0.01	-0.22 ± 0.01	-0.17 ± 0.01	-0.13 ± 0.01	-0.12 ± 0.01	-0.14 ± 0.01	-0.12 ± 0.01	-0.12 ± 0.01	-0.10 ± 0.01	-0.09 ± 0.01
Mammal	*Canis familiaris*	-0.17 ± 0.01	-0.24 ± 0.01	-0.31 ± 0.01	-0.30 ± 0.01	-0.26 ± 0.01	-0.20 ± 0.01	-0.17 ± 0.01	-0.18 ± 0.01	-0.18 ± 0.01	-0.14 ± 0.01	-0.13 ± 0.01	-0.12 ± 0.01	-0.12 ± 0.01
Mammal	*Equus caballus*	-0.11 ± 0.01	-0.16 ± 0.01	-0.24 ± 0.01	-0.20 ± 0.01	-0.17 ± 0.01	-0.14 ± 0.01	-0.11 ± 0.01	-0.10 ± 0.01	-0.11 ± 0.01	-0.11 ± 0.01	-0.11 ± 0.01	-0.12 ± 0.01	-0.10 ± 0.01
Mammal	*Homo sapiens*	-0.17 ± 0.01	-0.22 ± 0.01	-0.26 ± 0.01	-0.25 ± 0.01	-0.23 ± 0.01	-0.20 ± 0.01	-0.16 ± 0.01	-0.15 ± 0.01	-0.15 ± 0.01	-0.14 ± 0.01	-0.14 ± 0.01	-0.12 ± 0.01	-0.12 ± 0.01
Mammal	*Loxodonta africana*	-0.08 ± 0.02	-0.18 ± 0.02	-0.24 ± 0.02	-0.22 ± 0.02	-0.16 ± 0.02	-0.17 ± 0.02	-0.15 ± 0.02	-0.15 ± 0.02	-0.13 ± 0.02	-0.11 ± 0.02	-0.10 ± 0.02	-0.12 ± 0.02	-0.08 ± 0.02
Mammal	*Mus musculus*	-0.16 ± 0.01	-0.19 ± 0.01	-0.25 ± 0.01	-0.24 ± 0.01	-0.20 ± 0.01	-0.18 ± 0.01	-0.15 ± 0.01	-0.12 ± 0.01	-0.13 ± 0.01	-0.12 ± 0.01	-0.11 ± 0.01	-0.10 ± 0.01	-0.10 ± 0.01
Mammal	*Pteropus vampyrus*	-0.14 ± 0.01	-0.21 ± 0.01	-0.26 ± 0.01	-0.26 ± 0.01	-0.21 ± 0.01	-0.19 ± 0.01	-0.17 ± 0.01	-0.16 ± 0.01	-0.14 ± 0.01	-0.14 ± 0.01	-0.11 ± 0.01	-0.12 ± 0.01	-0.11 ± 0.01
Mammal	*Tursiops truncatus*	-0.12 ± 0.01	-0.19 ± 0.01	-0.25 ± 0.01	-0.26 ± 0.01	-0.23 ± 0.01	-0.18 ± 0.01	-0.15 ± 0.01	-0.15 ± 0.01	-0.13 ± 0.01	-0.11 ± 0.01	-0.11 ± 0.01	-0.10 ± 0.01	-0.11 ± 0.01
