# Supplementary material for: A Universal Trend of Reduced mRNA Stability near the Translation-Initiation Site in Prokaryotes and Eukaryotes
Source: PLoS Comput Biol. 2010 Feb 5;6(2):e1000664. doi: 10.1371/journal.pcbi.1000664 (PMC2816680; doi:10.1371/journal.pcbi.1000664)
Supplement: Table S2 — Mean and standard error of ΔG for each window. (0.08 MB PDF) [file pcbi.1000664.s002.pdf]

Table S2: Mean and standard error of ΔG for each window

Type	Species	Window 1	Window 2	Window 3	Window 4	Window 5	Window 6	Window 7	Window 8	Window 9	Window 10	Window 11	Window 12	Window 13
Bacteria	*Acaryochloris marina*	−2.58 ± 0.03	−2.83 ± 0.03	−3.47 ± 0.04	−3.96 ± 0.04	−3.97 ± 0.04	−3.99 ± 0.04	−4.00 ± 0.04	−3.92 ± 0.04	−3.93 ± 0.04	−3.94 ± 0.04	−3.93 ± 0.04	−3.92 ± 0.03	−3.93 ± 0.04
Bacteria	*Acholeplasma laidlawii*	−1.29 ± 0.04	−1.64 ± 0.05	−2.13 ± 0.06	−2.29 ± 0.06	−2.17 ± 0.06	−2.41 ± 0.06	−2.34 ± 0.06	−2.25 ± 0.06	−2.36 ± 0.06	−2.32 ± 0.06	−2.26 ± 0.06	−2.35 ± 0.06	−2.32 ± 0.06
Bacteria	*Acidiphilium cryptum*	−5.03 ± 0.06	−6.14 ± 0.07	−7.43 ± 0.07	−7.89 ± 0.07	−8.12 ± 0.07	−8.22 ± 0.07	−8.10 ± 0.07	−8.15 ± 0.07	−8.03 ± 0.07	−7.96 ± 0.07	−7.88 ± 0.07	−7.92 ± 0.07	−7.75 ± 0.07
Bacteria	*Acidithiobacillus ferrooxidans*	−3.76 ± 0.05	−4.53 ± 0.06	−5.44 ± 0.06	−6.01 ± 0.06	−6.08 ± 0.06	−6.21 ± 0.06	−6.07 ± 0.06	−5.99 ± 0.06	−6.04 ± 0.06	−5.91 ± 0.06	−5.94 ± 0.06	−6.01 ± 0.06	−5.89 ± 0.06
Bacteria	*Acidobacteria bacterium*	−3.78 ± 0.04	−4.12 ± 0.04	−4.86 ± 0.04	−5.44 ± 0.05	−5.69 ± 0.05	−5.71 ± 0.05	−5.67 ± 0.05	−5.66 ± 0.05	−5.59 ± 0.05	−5.62 ± 0.04	−5.67 ± 0.04	−5.70 ± 0.04	−5.68 ± 0.04
Bacteria	*Acidobacterium capsulatum*	−3.96 ± 0.05	−4.49 ± 0.06	−5.42 ± 0.06	−6.01 ± 0.06	−6.17 ± 0.06	−6.35 ± 0.06	−6.34 ± 0.06	−6.35 ± 0.06	−6.40 ± 0.06	−6.31 ± 0.06	−6.27 ± 0.06	−6.30 ± 0.06	−6.28 ± 0.06
Bacteria	*Acidothermus cellulolyticus*	−6.19 ± 0.06	−6.87 ± 0.07	−7.75 ± 0.08	−7.81 ± 0.08	−7.80 ± 0.07	−7.81 ± 0.08	−7.75 ± 0.08	−7.68 ± 0.07	−7.73 ± 0.08	−7.59 ± 0.07	−7.59 ± 0.07	−7.67 ± 0.07	−7.54 ± 0.07
Bacteria	*Acidovorax citrulli*	−4.02 ± 0.05	−5.31 ± 0.05	−6.94 ± 0.06	−7.63 ± 0.06	−7.89 ± 0.06	−8.02 ± 0.06	−8.01 ± 0.06	−8.06 ± 0.06	−8.07 ± 0.05	−8.04 ± 0.05	−8.08 ± 0.05	−8.10 ± 0.05	−7.99 ± 0.05
Bacteria	*Acinetobacter baumannii*	−2.02 ± 0.03	−2.37 ± 0.04	−2.97 ± 0.04	−3.18 ± 0.04	−3.24 ± 0.04	−3.18 ± 0.04	−3.10 ± 0.04	−3.02 ± 0.04	−3.05 ± 0.04	−3.01 ± 0.04	−2.96 ± 0.04	−3.03 ± 0.04	−3.10 ± 0.04
Bacteria	*Actinobacillus pleuropneumoniae*	−1.71 ± 0.04	−2.32 ± 0.05	−3.23 ± 0.06	−3.42 ± 0.06	−3.35 ± 0.06	−3.39 ± 0.06	−3.31 ± 0.06	−3.29 ± 0.06	−3.40 ± 0.06	−3.41 ± 0.06	−3.44 ± 0.06	−3.44 ± 0.06	−3.50 ± 0.06
Bacteria	*Aeromonas hydrophila*	−3.44 ± 0.04	−4.56 ± 0.05	−5.86 ± 0.06	−6.14 ± 0.06	−6.37 ± 0.06	−6.33 ± 0.06	−6.20 ± 0.05	−6.19 ± 0.05	−6.35 ± 0.05	−6.39 ± 0.05	−6.50 ± 0.06	−6.60 ± 0.05	−6.47 ± 0.05
Bacteria	*Agrobacterium radiobacter*	−3.58 ± 0.03	−4.41 ± 0.04	−5.66 ± 0.04	−6.16 ± 0.04	−6.36 ± 0.04	−6.35 ± 0.04	−6.29 ± 0.04	−6.19 ± 0.04	−6.29 ± 0.04	−6.28 ± 0.04	−6.24 ± 0.04	−6.33 ± 0.04	−6.25 ± 0.04
Bacteria	*Akkermansia muciniphila*	−2.40 ± 0.05	−3.30 ± 0.06	−4.87 ± 0.07	−5.68 ± 0.08	−5.76 ± 0.08	−5.73 ± 0.07	−5.54 ± 0.07	−5.48 ± 0.07	−5.40 ± 0.07	−5.42 ± 0.07	−5.37 ± 0.07	−5.44 ± 0.07	−5.40 ± 0.07
Bacteria	*Alcanivorax borkumensis*	−3.20 ± 0.05	−3.97 ± 0.06	−5.01 ± 0.06	−5.41 ± 0.06	−5.48 ± 0.06	−5.51 ± 0.06	−5.51 ± 0.06	−5.45 ± 0.06	−5.35 ± 0.06	−5.24 ± 0.06	−5.22 ± 0.06	−5.41 ± 0.06	−5.33 ± 0.06
Bacteria	*Aliivibrio salmonicida*	−1.54 ± 0.03	−2.05 ± 0.03	−2.77 ± 0.04	−2.91 ± 0.04	−3.22 ± 0.04	−3.30 ± 0.05	−3.13 ± 0.04	−2.82 ± 0.04	−2.91 ± 0.04	−2.96 ± 0.04	−3.00 ± 0.04	−3.18 ± 0.04	−3.16 ± 0.04
Bacteria	*Alkalilimnicola ehrlichii*	−4.47 ± 0.05	−5.46 ± 0.06	−6.91 ± 0.07	−7.63 ± 0.07	−7.99 ± 0.07	−8.07 ± 0.07	−7.99 ± 0.07	−8.02 ± 0.07	−7.96 ± 0.07	−7.80 ± 0.07	−7.85 ± 0.07	−7.89 ± 0.07	−7.71 ± 0.07
Bacteria	*Alkaliphilus metalliredigens*	−1.53 ± 0.02	−1.80 ± 0.03	−2.13 ± 0.03	−2.31 ± 0.03	−2.37 ± 0.03	−2.44 ± 0.03	−2.55 ± 0.03	−2.49 ± 0.03	−2.53 ± 0.03	−2.51 ± 0.03	−2.58 ± 0.03	−2.57 ± 0.03	−2.48 ± 0.03
Bacteria	*Alteromonas macleodii*	−2.48 ± 0.04	−2.80 ± 0.04	−3.53 ± 0.05	−3.68 ± 0.04	−3.78 ± 0.04	−3.88 ± 0.04	−3.74 ± 0.04	−3.75 ± 0.04	−3.72 ± 0.04	−3.67 ± 0.04	−3.75 ± 0.04	−3.70 ± 0.04	−3.69 ± 0.04
Bacteria	*Anabaena variabilis*	−2.03 ± 0.03	−2.22 ± 0.03	−2.78 ± 0.03	−3.13 ± 0.04	−3.32 ± 0.04	−3.33 ± 0.04	−3.24 ± 0.04	−3.16 ± 0.03	−3.19 ± 0.03	−3.21 ± 0.03	−3.16 ± 0.03	−3.16 ± 0.03	−3.19 ± 0.03
Bacteria	*Anaerocellum thermophilum*	−1.78 ± 0.03	−1.97 ± 0.04	−2.18 ± 0.04	−2.29 ± 0.04	−2.22 ± 0.04	−2.20 ± 0.04	−2.28 ± 0.04	−2.26 ± 0.04	−2.28 ± 0.04	−2.33 ± 0.04	−2.35 ± 0.04	−2.33 ± 0.04	−2.36 ± 0.04
Bacteria	*Anaeromyxobacter dehalogenans*	−5.99 ± 0.05	−6.98 ± 0.05	−8.35 ± 0.06	−9.11 ± 0.06	−9.38 ± 0.06	−9.71 ± 0.06	−9.49 ± 0.06	−9.53 ± 0.06	−9.65 ± 0.06	−9.53 ± 0.06	−9.48 ± 0.06	−9.51 ± 0.06	−9.30 ± 0.06
Bacteria	*Anaplasma marginale*	−3.71 ± 0.08	−3.77 ± 0.09	−4.23 ± 0.09	−4.44 ± 0.09	−4.42 ± 0.09	−4.69 ± 0.09	−4.82 ± 0.09	−4.89 ± 0.10	−4.54 ± 0.09	−4.53 ± 0.09	−4.32 ± 0.09	−4.42 ± 0.09	−4.40 ± 0.09
Bacteria	*Anoxybacillus flavithermus*	−2.42 ± 0.04	−2.47 ± 0.04	−2.72 ± 0.05	−2.80 ± 0.05	−2.88 ± 0.05	−2.95 ± 0.05	−2.99 ± 0.05	−2.97 ± 0.05	−3.03 ± 0.05	−3.01 ± 0.05	−2.99 ± 0.05	−3.08 ± 0.05	−3.09 ± 0.05
Bacteria	*Aquifex aeolicus*	−2.58 ± 0.05	−2.75 ± 0.06	−2.94 ± 0.07	−3.07 ± 0.07	−3.21 ± 0.07	−3.14 ± 0.07	−2.95 ± 0.06	−2.94 ± 0.06	−2.99 ± 0.06	−3.11 ± 0.06	−3.12 ± 0.06	−3.10 ± 0.07	−3.14 ± 0.06
Bacteria	*Archaeoglobus fulgidus*	−3.26 ± 0.05	−3.47 ± 0.05	−3.71 ± 0.05	−3.72 ± 0.05	−3.85 ± 0.06	−3.85 ± 0.06	−3.90 ± 0.06	−3.84 ± 0.05	−3.87 ± 0.05	−3.88 ± 0.05	−3.79 ± 0.06	−3.83 ± 0.05	−3.75 ± 0.05
Bacteria	*Arcobacter butzleri*	−1.07 ± 0.03	−1.26 ± 0.03	−1.76 ± 0.04	−1.93 ± 0.05	−1.95 ± 0.05	−1.90 ± 0.04	−1.82 ± 0.04	−1.73 ± 0.04	−1.71 ± 0.04	−1.76 ± 0.04	−1.67 ± 0.04	−1.70 ± 0.04	−1.75 ± 0.04
Bacteria	*Aromatoleum aromaticum*	−4.44 ± 0.05	−5.28 ± 0.05	−6.39 ± 0.05	−6.88 ± 0.06	−7.09 ± 0.06	−7.16 ± 0.05	−7.12 ± 0.05	−7.14 ± 0.05	−7.11 ± 0.05	−7.08 ± 0.05	−7.11 ± 0.05	−7.09 ± 0.05	−7.06 ± 0.05
Bacteria	*Arthrobacter aurescens*	−4.28 ± 0.05	−5.05 ± 0.05	−5.91 ± 0.05	−6.24 ± 0.05	−6.23 ± 0.05	−6.41 ± 0.05	−6.50 ± 0.05	−6.45 ± 0.05	−6.46 ± 0.05	−6.34 ± 0.05	−6.25 ± 0.05	−6.35 ± 0.05	−6.29 ± 0.05
Bacteria	*Aster yellows*	−1.04 ± 0.05	−1.00 ± 0.06	−1.23 ± 0.07	−1.34 ± 0.08	−1.28 ± 0.07	−1.32 ± 0.07	−1.26 ± 0.06	−1.26 ± 0.07	−1.38 ± 0.07	−1.41 ± 0.07	−1.42 ± 0.07	−1.36 ± 0.07	−1.49 ± 0.07
Bacteria	*Azoarcus sp. BH72*	−4.13 ± 0.05	−5.44 ± 0.05	−7.08 ± 0.06	−7.54 ± 0.06	−7.80 ± 0.06	−7.84 ± 0.06	−7.84 ± 0.06	−7.74 ± 0.06	−7.86 ± 0.06	−7.81 ± 0.06	−7.77 ± 0.06	−7.77 ± 0.06	−7.61 ± 0.06
Bacteria	*Azorhizobium caulinodans*	−5.13 ± 0.05	−5.93 ± 0.05	−6.94 ± 0.05	−7.41 ± 0.06	−7.57 ± 0.06	−7.70 ± 0.05	−7.51 ± 0.05	−7.67 ± 0.05	−7.64 ± 0.05	−7.59 ± 0.05	−7.59 ± 0.05	−7.66 ± 0.05	−7.52 ± 0.05
Bacteria	*Azotobacter vinelandii*	−4.08 ± 0.04	−5.17 ± 0.05	−6.40 ± 0.05	−6.91 ± 0.05	−7.12 ± 0.05	−7.32 ± 0.05	−7.29 ± 0.05	−7.35 ± 0.05	−7.36 ± 0.05	−7.23 ± 0.05	−7.23 ± 0.05	−7.29 ± 0.05	−7.25 ± 0.05
Bacteria	*Bacillus amyloliquefaciens*	−2.21 ± 0.03	−2.74 ± 0.04	−3.37 ± 0.05	−3.48 ± 0.05	−3.63 ± 0.05	−3.67 ± 0.05	−3.76 ± 0.05	−3.73 ± 0.05	−3.74 ± 0.05	−3.80 ± 0.05	−3.80 ± 0.05	−3.82 ± 0.05	−3.77 ± 0.05
Bacteria	*Bacteroides fragilis*	−1.51 ± 0.03	−2.22 ± 0.04	−3.58 ± 0.05	−3.94 ± 0.05	−3.86 ± 0.05	−3.59 ± 0.04	−3.46 ± 0.04	−3.45 ± 0.04	−3.57 ± 0.04	−3.58 ± 0.04	−3.55 ± 0.04	−3.56 ± 0.04	−3.45 ± 0.04
Bacteria	*Bartonella bacilliformis*	−2.01 ± 0.05	−2.12 ± 0.06	−2.72 ± 0.07	−2.85 ± 0.07	−3.03 ± 0.08	−3.17 ± 0.07	−3.04 ± 0.07	−2.89 ± 0.07	−2.87 ± 0.07	−2.97 ± 0.07	−2.86 ± 0.07	−2.98 ± 0.07	−2.96 ± 0.07
Bacteria	*Baumannia cicadellinicola*	−1.70 ± 0.07	−2.16 ± 0.09	−2.60 ± 0.10	−2.65 ± 0.10	−2.62 ± 0.09	−2.56 ± 0.10	−2.64 ± 0.10	−2.32 ± 0.09	−2.52 ± 0.09	−2.65 ± 0.09	−2.46 ± 0.09	−2.40 ± 0.09	−2.47 ± 0.09
Bacteria	*Bdellovibrio bacteriovorus*	−2.55 ± 0.04	−2.93 ± 0.04	−3.38 ± 0.05	−3.94 ± 0.05	−4.71 ± 0.06	−4.76 ± 0.05	−4.65 ± 0.05	−4.46 ± 0.05	−4.55 ± 0.05	−4.46 ± 0.05	−4.45 ± 0.05	−4.50 ± 0.05	−4.40 ± 0.05
Bacteria	*Beijerinckia indica*	−3.65 ± 0.04	−4.31 ± 0.05	−5.34 ± 0.05	−5.77 ± 0.06	−5.90 ± 0.06	−5.96 ± 0.06	−5.94 ± 0.05	−5.90 ± 0.05	−5.85 ± 0.05	−5.83 ± 0.05	−5.79 ± 0.05	−5.80 ± 0.05	−5.81 ± 0.05

Continued on next page

Type	Species	Window 1	Window 2	Window 3	Window 4	Window 5	Window 6	Window 7	Window 8	Window 9	Window 10	Window 11	Window 12	Window 13
Bacteria	*Beutenbergia cavernae*	−6.37 ± 0.05	−7.64 ± 0.06	−8.81 ± 0.06	−8.94 ± 0.06	−9.09 ± 0.06	−9.21 ± 0.06	−9.15 ± 0.06	−9.09 ± 0.05	−9.13 ± 0.05	−9.01 ± 0.05	−9.00 ± 0.05	−8.94 ± 0.05	−8.88 ± 0.05
Bacteria	*Bifidobacterium adolescentis*	−4.01 ± 0.07	−4.52 ± 0.08	−5.39 ± 0.08	−5.69 ± 0.08	−5.73 ± 0.08	−5.88 ± 0.08	−5.80 ± 0.08	−5.82 ± 0.08	−5.90 ± 0.08	−6.00 ± 0.08	−5.78 ± 0.08	−5.76 ± 0.08	−5.71 ± 0.08
Bacteria	*Bordetella avium*	−3.44 ± 0.05	−4.71 ± 0.06	−6.15 ± 0.06	−6.69 ± 0.06	−6.78 ± 0.06	−6.83 ± 0.06	−6.78 ± 0.06	−6.70 ± 0.06	−6.86 ± 0.06	−6.74 ± 0.06	−6.70 ± 0.06	−6.79 ± 0.06	−6.74 ± 0.06
Bacteria	*Borrelia afzelii*	−1.31 ± 0.05	−1.33 ± 0.06	−1.54 ± 0.07	−1.72 ± 0.07	−1.76 ± 0.07	−1.77 ± 0.07	−1.69 ± 0.06	−1.70 ± 0.07	−1.71 ± 0.06	−1.70 ± 0.06	−1.73 ± 0.07	−1.77 ± 0.07	−1.80 ± 0.06
Bacteria	*Brachyspira hyodysenteriae*	−1.14 ± 0.03	−1.16 ± 0.03	−1.57 ± 0.04	−1.71 ± 0.04	−1.85 ± 0.04	−1.79 ± 0.04	−1.78 ± 0.04	−1.73 ± 0.04	−1.67 ± 0.04	−1.73 ± 0.04	−1.71 ± 0.04	−1.74 ± 0.04	−1.72 ± 0.04
Bacteria	*Bradyrhizobium japonicum*	−4.78 ± 0.03	−5.32 ± 0.03	−6.15 ± 0.04	−6.70 ± 0.04	−6.95 ± 0.04	−7.07 ± 0.04	−7.04 ± 0.04	−6.98 ± 0.04	−7.01 ± 0.04	−6.96 ± 0.04	−7.00 ± 0.04	−6.98 ± 0.04	−6.92 ± 0.04
Bacteria	*Brevibacillus brevis*	−2.68 ± 0.03	−3.05 ± 0.03	−3.53 ± 0.03	−3.71 ± 0.04	−3.71 ± 0.04	−3.78 ± 0.04	−3.79 ± 0.03	−3.71 ± 0.03	−3.77 ± 0.03	−3.75 ± 0.03	−3.71 ± 0.03	−3.83 ± 0.03	−3.77 ± 0.03
Bacteria	*Brucella abortus*	−3.46 ± 0.05	−4.16 ± 0.05	−5.23 ± 0.06	−5.85 ± 0.06	−6.07 ± 0.06	−6.12 ± 0.06	−5.92 ± 0.06	−5.90 ± 0.06	−5.89 ± 0.06	−5.74 ± 0.06	−5.83 ± 0.06	−5.95 ± 0.06	−5.98 ± 0.06
Bacteria	*Buchnera aphidicola*	−1.17 ± 0.06	−1.24 ± 0.07	−1.68 ± 0.08	−1.70 ± 0.08	−1.78 ± 0.09	−1.69 ± 0.08	−1.69 ± 0.08	−1.71 ± 0.08	−1.61 ± 0.08	−1.74 ± 0.08	−1.61 ± 0.07	−1.67 ± 0.08	−1.63 ± 0.07
Bacteria	*Burkholderia ambifaria*	−4.02 ± 0.04	−5.44 ± 0.04	−7.05 ± 0.05	−7.58 ± 0.05	−7.72 ± 0.05	−7.91 ± 0.05	−7.87 ± 0.05	−7.79 ± 0.05	−7.86 ± 0.04	−7.82 ± 0.05	−7.87 ± 0.05	−7.95 ± 0.04	−7.88 ± 0.04
Bacteria	*Caldicellulosiruptor saccharolyticus*	−1.82 ± 0.03	−1.93 ± 0.04	−2.14 ± 0.04	−2.23 ± 0.04	−2.26 ± 0.04	−2.20 ± 0.04	−2.33 ± 0.04	−2.30 ± 0.04	−2.27 ± 0.04	−2.36 ± 0.04	−2.31 ± 0.04	−2.44 ± 0.04	−2.36 ± 0.04
Bacteria	*Campylobacter concisus*	−1.81 ± 0.04	−2.27 ± 0.05	−3.05 ± 0.06	−3.22 ± 0.06	−3.23 ± 0.06	−3.17 ± 0.06	−3.10 ± 0.06	−3.07 ± 0.06	−3.11 ± 0.06	−3.11 ± 0.06	−3.17 ± 0.06	−3.02 ± 0.06	−3.14 ± 0.06
Bacteria	*Candidatus Amoebophilus*	−1.40 ± 0.04	−1.59 ± 0.05	−2.03 ± 0.06	−2.41 ± 0.07	−2.52 ± 0.07	−2.56 ± 0.06	−2.47 ± 0.06	−2.42 ± 0.06	−2.42 ± 0.06	−2.29 ± 0.06	−2.26 ± 0.06	−2.25 ± 0.06	−2.34 ± 0.06
Bacteria	*Carboxydothermus hydrogenoformans*	−2.38 ± 0.04	−2.64 ± 0.05	−3.09 ± 0.06	−3.13 ± 0.06	−3.12 ± 0.05	−3.21 ± 0.05	−3.25 ± 0.05	−3.17 ± 0.05	−3.35 ± 0.06	−3.32 ± 0.06	−3.31 ± 0.05	−3.26 ± 0.06	−3.25 ± 0.06
Bacteria	*Caulobacter crescentus*	−5.17 ± 0.05	−5.97 ± 0.06	−7.13 ± 0.06	−7.69 ± 0.06	−7.84 ± 0.06	−7.84 ± 0.06	−7.59 ± 0.06	−7.52 ± 0.06	−7.66 ± 0.06	−7.60 ± 0.06	−7.58 ± 0.06	−7.66 ± 0.06	−7.56 ± 0.06
Bacteria	*Cellvibrio japonicus*	−2.77 ± 0.04	−3.31 ± 0.05	−4.20 ± 0.05	−4.72 ± 0.06	−5.00 ± 0.05	−5.09 ± 0.05	−5.00 ± 0.05	−4.82 ± 0.05	−4.85 ± 0.05	−4.78 ± 0.05	−4.76 ± 0.05	−4.78 ± 0.05	−4.90 ± 0.05
Bacteria	*Chlamydia muridarum*	−2.07 ± 0.06	−2.31 ± 0.07	−2.80 ± 0.08	−2.82 ± 0.08	−3.03 ± 0.09	−3.21 ± 0.09	−3.12 ± 0.08	−3.19 ± 0.09	−3.17 ± 0.09	−3.13 ± 0.08	−3.12 ± 0.08	−3.14 ± 0.08	−3.03 ± 0.08
Bacteria	*Chlamydophila abortus*	−1.98 ± 0.06	−2.10 ± 0.07	−2.74 ± 0.08	−2.85 ± 0.08	−2.94 ± 0.08	−3.09 ± 0.08	−2.99 ± 0.08	−2.94 ± 0.08	−2.96 ± 0.08	−2.95 ± 0.08	−2.87 ± 0.08	−2.92 ± 0.08	−2.99 ± 0.08
Bacteria	*Chlorobaculum parvum*	−2.69 ± 0.05	−3.59 ± 0.06	−4.73 ± 0.07	−5.28 ± 0.07	−5.34 ± 0.07	−5.39 ± 0.07	−5.31 ± 0.07	−5.38 ± 0.07	−5.39 ± 0.07	−5.37 ± 0.07	−5.37 ± 0.07	−5.31 ± 0.07	−5.35 ± 0.07
Bacteria	*Chlorobium chlorochromatii*	−2.06 ± 0.04	−2.48 ± 0.05	−3.40 ± 0.06	−3.99 ± 0.07	−3.95 ± 0.06	−3.78 ± 0.06	−3.67 ± 0.06	−3.70 ± 0.06	−3.75 ± 0.06	−3.76 ± 0.06	−3.70 ± 0.06	−3.78 ± 0.06	−3.67 ± 0.06
Bacteria	*Chloroflexus aggregans*	−3.47 ± 0.04	−3.90 ± 0.05	−4.77 ± 0.05	−5.23 ± 0.05	−5.47 ± 0.05	−5.54 ± 0.05	−5.54 ± 0.05	−5.39 ± 0.05	−5.41 ± 0.05	−5.45 ± 0.05	−5.41 ± 0.05	−5.39 ± 0.05	−5.36 ± 0.05
Bacteria	*Chloroherpeton thalassium*	−2.06 ± 0.04	−2.47 ± 0.04	−3.34 ± 0.05	−3.68 ± 0.05	−3.75 ± 0.05	−3.74 ± 0.05	−3.57 ± 0.05	−3.70 ± 0.05	−3.80 ± 0.05	−3.80 ± 0.05	−3.66 ± 0.05	−3.80 ± 0.05	−3.82 ± 0.05
Bacteria	*Chromobacterium violaceum*	−4.17 ± 0.05	−5.36 ± 0.05	−6.77 ± 0.06	−7.19 ± 0.06	−7.25 ± 0.06	−7.46 ± 0.06	−7.33 ± 0.06	−7.33 ± 0.06	−7.35 ± 0.06	−7.29 ± 0.06	−7.34 ± 0.06	−7.33 ± 0.06	−7.29 ± 0.06
Bacteria	*Chromohalobacter salexigens*	−3.95 ± 0.05	−5.11 ± 0.05	−6.41 ± 0.06	−6.79 ± 0.06	−6.86 ± 0.06	−7.01 ± 0.06	−6.94 ± 0.06	−6.95 ± 0.06	−7.04 ± 0.06	−7.00 ± 0.06	−6.97 ± 0.06	−7.02 ± 0.06	−6.98 ± 0.06
Bacteria	*Citrobacter koseri*	−3.17 ± 0.04	−3.82 ± 0.04	−4.76 ± 0.05	−4.91 ± 0.05	−5.03 ± 0.05	−4.99 ± 0.05	−4.88 ± 0.05	−4.94 ± 0.05	−5.05 ± 0.05	−5.03 ± 0.05	−5.01 ± 0.05	−5.08 ± 0.05	−5.11 ± 0.05
Bacteria	*Clavibacter michiganensis*	−5.16 ± 0.06	−6.66 ± 0.07	−8.05 ± 0.07	−8.39 ± 0.07	−8.53 ± 0.07	−8.68 ± 0.07	−8.61 ± 0.06	−8.57 ± 0.06	−8.70 ± 0.06	−8.56 ± 0.06	−8.53 ± 0.06	−8.50 ± 0.06	−8.37 ± 0.06
Bacteria	*Clostridium acetobutylicum*	−1.25 ± 0.03	−1.52 ± 0.03	−1.73 ± 0.03	−1.93 ± 0.03	−1.86 ± 0.03	−1.91 ± 0.03	−1.91 ± 0.03	−1.86 ± 0.03	−1.90 ± 0.03	−1.97 ± 0.03	−1.94 ± 0.03	−1.93 ± 0.03	−1.95 ± 0.03
Bacteria	*Colwellia psychrerythraea*	−1.51 ± 0.03	−1.88 ± 0.03	−2.57 ± 0.04	−2.94 ± 0.04	−3.01 ± 0.04	−3.02 ± 0.04	−3.02 ± 0.04	−2.83 ± 0.04	−2.90 ± 0.04	−2.87 ± 0.04	−2.89 ± 0.03	−2.92 ± 0.03	−2.89 ± 0.04
Bacteria	*Coprothermobacter proteolyticus*	−2.75 ± 0.06	−2.98 ± 0.06	−3.40 ± 0.07	−3.56 ± 0.07	−3.55 ± 0.07	−3.57 ± 0.07	−3.66 ± 0.07	−3.57 ± 0.07	−3.63 ± 0.07	−3.54 ± 0.07	−3.58 ± 0.07	−3.53 ± 0.07	−3.48 ± 0.06
Bacteria	*Corynebacterium aurimucosum*	−3.59 ± 0.05	−4.35 ± 0.06	−5.60 ± 0.07	−5.78 ± 0.07	−6.12 ± 0.07	−6.07 ± 0.07	−6.04 ± 0.07	−6.13 ± 0.07	−6.12 ± 0.06	−6.15 ± 0.06	−6.05 ± 0.06	−6.16 ± 0.07	−6.09 ± 0.06
Bacteria	*Coxiella burnetii*	−2.13 ± 0.05	−2.36 ± 0.06	−2.96 ± 0.07	−3.19 ± 0.07	−3.32 ± 0.07	−3.48 ± 0.07	−3.62 ± 0.07	−3.43 ± 0.07	−3.43 ± 0.07	−3.32 ± 0.06	−3.45 ± 0.07	−3.46 ± 0.07	−3.36 ± 0.07
Bacteria	*Cronobacter sakazakii*	−3.24 ± 0.04	−4.19 ± 0.05	−5.39 ± 0.06	−5.63 ± 0.05	−5.65 ± 0.05	−5.67 ± 0.05	−5.64 ± 0.05	−5.63 ± 0.05	−5.75 ± 0.05	−5.75 ± 0.05	−5.75 ± 0.05	−5.82 ± 0.05	−5.75 ± 0.05
Bacteria	*Cupriavidus taiwanensis*	−4.21 ± 0.04	−5.57 ± 0.05	−7.06 ± 0.05	−7.66 ± 0.05	−7.98 ± 0.05	−8.15 ± 0.05	−8.07 ± 0.05	−8.13 ± 0.05	−8.16 ± 0.05	−8.05 ± 0.05	−8.03 ± 0.05	−8.09 ± 0.05	−8.01 ± 0.05
Bacteria	*Cyanothece sp. PCC 7424*	−1.61 ± 0.02	−1.76 ± 0.03	−2.22 ± 0.03	−2.60 ± 0.03	−2.81 ± 0.03	−2.90 ± 0.03	−2.78 ± 0.03	−2.70 ± 0.03	−2.68 ± 0.03	−2.67 ± 0.03	−2.64 ± 0.03	−2.65 ± 0.03	−2.61 ± 0.03
Bacteria	*Cytophaga hutchinsonii*	−1.47 ± 0.03	−1.71 ± 0.03	−2.47 ± 0.04	−2.99 ± 0.04	−3.07 ± 0.04	−2.97 ± 0.04	−2.81 ± 0.04	−2.77 ± 0.04	−2.84 ± 0.04	−2.82 ± 0.04	−2.90 ± 0.04	−2.91 ± 0.04	−2.81 ± 0.04
Bacteria	*Dechloromonas aromatica*	−3.15 ± 0.04	−4.09 ± 0.05	−5.46 ± 0.05	−6.12 ± 0.05	−6.20 ± 0.05	−6.23 ± 0.05	−6.07 ± 0.05	−6.03 ± 0.05	−6.12 ± 0.05	−6.10 ± 0.05	−6.11 ± 0.05	−6.14 ± 0.05	−6.07 ± 0.05
Bacteria	*Dehalococcoides ethenogenes*	−2.54 ± 0.06	−3.16 ± 0.07	−3.76 ± 0.07	−4.27 ± 0.08	−4.62 ± 0.08	−4.78 ± 0.08	−4.62 ± 0.08	−4.54 ± 0.08	−4.60 ± 0.08	−4.41 ± 0.07	−4.56 ± 0.08	−4.48 ± 0.08	−4.47 ± 0.07
Bacteria	*Deinococcus deserti*	−4.03 ± 0.06	−4.78 ± 0.06	−6.00 ± 0.07	−6.58 ± 0.07	−6.84 ± 0.07	−6.93 ± 0.07	−6.93 ± 0.06	−6.71 ± 0.07	−6.83 ± 0.06	−6.72 ± 0.06	−6.72 ± 0.06	−6.82 ± 0.06	−6.70 ± 0.06
Bacteria	*Delftia acidovorans*	−3.64 ± 0.04	−4.89 ± 0.04	−6.54 ± 0.05	−7.20 ± 0.05	−7.43 ± 0.05	−7.63 ± 0.05	−7.54 ± 0.05	−7.60 ± 0.05	−7.70 ± 0.05	−7.61 ± 0.05	−7.60 ± 0.05	−7.69 ± 0.05	−7.63 ± 0.05

Continued on next page

Type	Species	Window 1	Window 2	Window 3	Window 4	Window 5	Window 6	Window 7	Window 8	Window 9	Window 10	Window 11	Window 12	Window 13
Bacteria	*Desulfatibacillum alkenivorans*	−2.53 ± 0.03	−3.31 ± 0.04	−4.29 ± 0.04	−4.91 ± 0.05	−5.14 ± 0.05	−5.33 ± 0.05	−5.35 ± 0.05	−5.32 ± 0.04	−5.37 ± 0.04	−5.22 ± 0.04	−5.35 ± 0.04	−5.37 ± 0.04	−5.32 ± 0.04
Bacteria	*Desulfitobacterium hafniense*	−2.40 ± 0.03	−2.97 ± 0.04	−3.46 ± 0.04	−3.70 ± 0.04	−3.84 ± 0.04	−3.99 ± 0.04	−4.04 ± 0.04	−4.05 ± 0.04	−4.11 ± 0.04	−4.22 ± 0.04	−4.16 ± 0.04	−4.20 ± 0.04	−4.15 ± 0.04
Bacteria	*Desulfobacterium autotrophicum*	−2.28 ± 0.03	−2.80 ± 0.04	−3.50 ± 0.04	−3.90 ± 0.04	−4.00 ± 0.04	−4.17 ± 0.04	−4.19 ± 0.04	−4.18 ± 0.04	−4.21 ± 0.04	−4.19 ± 0.04	−4.22 ± 0.04	−4.27 ± 0.04	−4.21 ± 0.04
Bacteria	*Desulfococcus oleovorans*	−2.77 ± 0.04	−3.58 ± 0.05	−4.65 ± 0.06	−5.34 ± 0.06	−5.74 ± 0.06	−5.84 ± 0.06	−5.84 ± 0.06	−5.77 ± 0.06	−5.80 ± 0.06	−5.69 ± 0.06	−5.74 ± 0.06	−5.72 ± 0.06	−5.61 ± 0.06
Bacteria	*Desulfotalea psychrophila*	−2.16 ± 0.04	−2.50 ± 0.04	−3.06 ± 0.05	−3.50 ± 0.05	−3.58 ± 0.05	−3.73 ± 0.05	−3.81 ± 0.05	−3.73 ± 0.05	−3.75 ± 0.05	−3.75 ± 0.05	−3.77 ± 0.05	−3.84 ± 0.05	−3.89 ± 0.05
Bacteria	*Desulfotomaculum reducens*	−2.33 ± 0.04	−2.67 ± 0.04	−3.15 ± 0.05	−3.20 ± 0.05	−3.37 ± 0.05	−3.42 ± 0.05	−3.35 ± 0.05	−3.31 ± 0.05	−3.45 ± 0.05	−3.44 ± 0.05	−3.43 ± 0.05	−3.41 ± 0.05	−3.32 ± 0.05
Bacteria	*Desulfovibrio desulfuricans*	−3.24 ± 0.05	−4.19 ± 0.06	−5.47 ± 0.07	−5.95 ± 0.07	−6.11 ± 0.07	−6.29 ± 0.07	−6.11 ± 0.07	−6.10 ± 0.07	−6.11 ± 0.07	−6.03 ± 0.07	−5.93 ± 0.07	−6.15 ± 0.07	−5.94 ± 0.07
Bacteria	*Diaphorobacter sp. TPSY*	−3.43 ± 0.05	−4.74 ± 0.06	−6.79 ± 0.07	−7.71 ± 0.07	−7.87 ± 0.07	−7.97 ± 0.06	−7.85 ± 0.06	−7.79 ± 0.06	−7.92 ± 0.06	−7.83 ± 0.06	−7.87 ± 0.06	−7.93 ± 0.06	−7.73 ± 0.06
Bacteria	*Dichelobacter nodosus*	−2.20 ± 0.06	−2.84 ± 0.07	−3.62 ± 0.08	−3.70 ± 0.08	−3.82 ± 0.08	−3.92 ± 0.08	−3.85 ± 0.08	−3.70 ± 0.08	−3.80 ± 0.08	−3.77 ± 0.08	−3.75 ± 0.08	−3.83 ± 0.07	−3.75 ± 0.08
Bacteria	*Dickeya dadantii*	−2.73 ± 0.03	−3.81 ± 0.05	−5.12 ± 0.05	−5.40 ± 0.05	−5.44 ± 0.05	−5.42 ± 0.05	−5.49 ± 0.05	−5.36 ± 0.05	−5.43 ± 0.05	−5.49 ± 0.05	−5.51 ± 0.05	−5.54 ± 0.05	−5.44 ± 0.05
Bacteria	*Dictyoglomus thermophilum*	−1.76 ± 0.04	−1.74 ± 0.04	−1.96 ± 0.05	−2.10 ± 0.05	−2.23 ± 0.05	−2.36 ± 0.06	−2.31 ± 0.05	−2.36 ± 0.05	−2.25 ± 0.05	−2.36 ± 0.05	−2.31 ± 0.05	−2.34 ± 0.05	−2.29 ± 0.05
Bacteria	*Dinoroseobacter shibae*	−4.05 ± 0.05	−5.21 ± 0.06	−6.74 ± 0.06	−7.39 ± 0.06	−7.47 ± 0.06	−7.55 ± 0.06	−7.37 ± 0.06	−7.32 ± 0.06	−7.38 ± 0.06	−7.40 ± 0.06	−7.36 ± 0.06	−7.40 ± 0.06	−7.32 ± 0.06
Bacteria	*Edwardsiella ictaluri*	−3.36 ± 0.05	−4.28 ± 0.05	−5.42 ± 0.06	−5.69 ± 0.06	−5.84 ± 0.06	−5.96 ± 0.06	−5.90 ± 0.06	−5.85 ± 0.06	−5.89 ± 0.06	−5.85 ± 0.06	−5.95 ± 0.06	−6.00 ± 0.06	−5.89 ± 0.06
Bacteria	*Ehrlichia canis*	−1.48 ± 0.05	−1.62 ± 0.06	−2.00 ± 0.06	−2.12 ± 0.07	−2.09 ± 0.07	−2.06 ± 0.07	−1.99 ± 0.06	−1.85 ± 0.06	−1.87 ± 0.06	−1.93 ± 0.06	−1.88 ± 0.06	−1.94 ± 0.06	−1.94 ± 0.06
Bacteria	*Elusimicrobium minutum*	−1.31 ± 0.04	−1.81 ± 0.05	−2.69 ± 0.07	−3.27 ± 0.07	−3.59 ± 0.07	−3.68 ± 0.08	−3.40 ± 0.07	−3.24 ± 0.07	−3.27 ± 0.07	−3.32 ± 0.07	−3.33 ± 0.07	−3.26 ± 0.07	−3.14 ± 0.07
Bacteria	*Enterobacter sp. 638*	−2.76 ± 0.04	−3.66 ± 0.04	−4.86 ± 0.05	−5.14 ± 0.05	−5.16 ± 0.05	−5.17 ± 0.05	−5.07 ± 0.05	−4.96 ± 0.05	−5.04 ± 0.05	−5.12 ± 0.05	−5.05 ± 0.05	−5.13 ± 0.05	−5.12 ± 0.05
Bacteria	*Enterococcus faecalis*	−1.62 ± 0.03	−2.00 ± 0.04	−2.45 ± 0.04	−2.59 ± 0.04	−2.64 ± 0.04	−2.73 ± 0.04	−2.75 ± 0.04	−2.61 ± 0.04	−2.72 ± 0.04	−2.73 ± 0.04	−2.61 ± 0.04	−2.61 ± 0.04	−2.67 ± 0.04
Bacteria	*Erwinia tasmaniensis*	−2.76 ± 0.04	−3.72 ± 0.05	−4.84 ± 0.06	−5.11 ± 0.06	−5.21 ± 0.06	−5.32 ± 0.06	−5.25 ± 0.06	−5.15 ± 0.06	−5.21 ± 0.05	−5.16 ± 0.05	−5.23 ± 0.06	−5.30 ± 0.06	−5.15 ± 0.05
Bacteria	*Erythrobacter litoralis*	−4.18 ± 0.05	−5.03 ± 0.06	−6.42 ± 0.06	−7.15 ± 0.06	−7.24 ± 0.06	−7.25 ± 0.06	−7.04 ± 0.06	−6.97 ± 0.06	−6.95 ± 0.06	−6.75 ± 0.06	−6.79 ± 0.06	−6.78 ± 0.06	−6.77 ± 0.06
Bacteria	*Escherichia coli*	−2.62 ± 0.03	−3.55 ± 0.04	−4.67 ± 0.05	−4.88 ± 0.05	−4.79 ± 0.05	−4.82 ± 0.05	−4.75 ± 0.05	−4.67 ± 0.05	−4.70 ± 0.05	−4.73 ± 0.05	−4.70 ± 0.05	−4.76 ± 0.05	−4.65 ± 0.05
Bacteria	*Eubacterium eligens*	−1.71 ± 0.04	−2.12 ± 0.05	−2.65 ± 0.06	−2.75 ± 0.05	−2.79 ± 0.06	−2.87 ± 0.05	−2.89 ± 0.06	−2.84 ± 0.05	−2.86 ± 0.05	−2.94 ± 0.05	−2.81 ± 0.05	−2.83 ± 0.05	−2.82 ± 0.05
Bacteria	*Exiguobacterium sibiricum*	−2.21 ± 0.04	−2.89 ± 0.04	−3.67 ± 0.05	−3.83 ± 0.05	−3.88 ± 0.05	−3.85 ± 0.05	−3.87 ± 0.05	−3.91 ± 0.05	−3.92 ± 0.05	−4.02 ± 0.05	−4.03 ± 0.05	−4.07 ± 0.05	−4.08 ± 0.05
Bacteria	*Fervidobacterium nodosum*	−1.74 ± 0.04	−1.75 ± 0.04	−2.01 ± 0.05	−2.07 ± 0.05	−2.04 ± 0.05	−2.12 ± 0.05	−2.06 ± 0.05	−2.21 ± 0.05	−2.32 ± 0.05	−2.34 ± 0.05	−2.33 ± 0.06	−2.32 ± 0.05	−2.32 ± 0.05
Bacteria	*Finegoldia magna*	−1.17 ± 0.04	−1.58 ± 0.05	−2.05 ± 0.06	−2.14 ± 0.05	−2.07 ± 0.05	−2.18 ± 0.05	−2.20 ± 0.05	−2.17 ± 0.05	−2.16 ± 0.05	−2.04 ± 0.05	−2.14 ± 0.05	−2.15 ± 0.05	−2.07 ± 0.05
Bacteria	*Flavobacterium johnsoniae*	−1.03 ± 0.02	−1.42 ± 0.02	−2.15 ± 0.03	−2.59 ± 0.03	−2.61 ± 0.03	−2.40 ± 0.03	−2.34 ± 0.03	−2.31 ± 0.03	−2.35 ± 0.03	−2.39 ± 0.03	−2.38 ± 0.03	−2.43 ± 0.03	−2.42 ± 0.03
Bacteria	*Francisella novicida*	−1.22 ± 0.03	−1.58 ± 0.04	−2.22 ± 0.05	−2.38 ± 0.05	−2.49 ± 0.05	−2.44 ± 0.05	−2.45 ± 0.05	−2.28 ± 0.05	−2.31 ± 0.05	−2.34 ± 0.05	−2.23 ± 0.05	−2.19 ± 0.05	−2.19 ± 0.05
Bacteria	*Frankia alni*	−7.27 ± 0.04	−7.98 ± 0.05	−8.49 ± 0.05	−8.55 ± 0.05	−8.61 ± 0.05	−8.72 ± 0.05	−8.61 ± 0.05	−8.68 ± 0.05	−8.74 ± 0.05	−8.62 ± 0.05	−8.59 ± 0.05	−8.68 ± 0.04	−8.61 ± 0.05
Bacteria	*Fusobacterium nucleatum*	−1.18 ± 0.03	−1.34 ± 0.04	−1.58 ± 0.04	−1.65 ± 0.04	−1.58 ± 0.04	−1.62 ± 0.04	−1.65 ± 0.04	−1.66 ± 0.04	−1.65 ± 0.04	−1.70 ± 0.04	−1.68 ± 0.04	−1.68 ± 0.04	−1.66 ± 0.04
Bacteria	*Gemmatimonas aurantiaca*	−5.00 ± 0.05	−5.86 ± 0.05	−6.95 ± 0.06	−7.33 ± 0.06	−7.34 ± 0.05	−7.48 ± 0.06	−7.34 ± 0.05	−7.30 ± 0.05	−7.34 ± 0.05	−7.34 ± 0.05	−7.22 ± 0.05	−7.30 ± 0.05	−7.25 ± 0.05
Bacteria	*Geobacillus kaustophilus*	−3.29 ± 0.04	−3.85 ± 0.05	−4.53 ± 0.06	−4.60 ± 0.05	−4.72 ± 0.06	−4.82 ± 0.06	−4.73 ± 0.05	−4.75 ± 0.05	−4.85 ± 0.05	−4.83 ± 0.05	−4.82 ± 0.05	−4.91 ± 0.05	−4.85 ± 0.05
Bacteria	*Geobacter bemidjiensis*	−3.01 ± 0.04	−3.80 ± 0.04	−4.80 ± 0.05	−5.47 ± 0.05	−5.84 ± 0.05	−6.02 ± 0.05	−6.00 ± 0.05	−5.98 ± 0.05	−6.02 ± 0.05	−5.94 ± 0.05	−5.94 ± 0.05	−6.02 ± 0.05	−6.00 ± 0.05
Bacteria	*Gloeobacter violaceus*	−4.17 ± 0.04	−4.71 ± 0.04	−5.64 ± 0.05	−6.10 ± 0.05	−6.41 ± 0.05	−6.44 ± 0.05	−6.39 ± 0.05	−6.39 ± 0.05	−6.46 ± 0.05	−6.38 ± 0.05	−6.29 ± 0.05	−6.44 ± 0.05	−6.34 ± 0.05
Bacteria	*Gluconacetobacter diazotrophicus*	−4.53 ± 0.05	−5.69 ± 0.06	−7.11 ± 0.06	−7.49 ± 0.06	−7.81 ± 0.07	−8.05 ± 0.06	−7.93 ± 0.06	−7.88 ± 0.06	−7.86 ± 0.06	−7.76 ± 0.06	−7.80 ± 0.06	−7.88 ± 0.06	−7.72 ± 0.06
Bacteria	*Gluconobacter oxydans*	−3.90 ± 0.06	−4.78 ± 0.06	−5.91 ± 0.07	−6.18 ± 0.07	−6.42 ± 0.07	−6.40 ± 0.07	−6.37 ± 0.07	−6.32 ± 0.07	−6.42 ± 0.06	−6.42 ± 0.06	−6.31 ± 0.06	−6.36 ± 0.06	−6.46 ± 0.07
Bacteria	*Gramella forsetii*	−1.35 ± 0.03	−1.70 ± 0.03	−2.42 ± 0.04	−2.85 ± 0.04	−2.99 ± 0.04	−2.82 ± 0.04	−2.69 ± 0.04	−2.64 ± 0.04	−2.69 ± 0.04	−2.82 ± 0.04	−2.69 ± 0.04	−2.71 ± 0.04	−2.66 ± 0.04
Bacteria	*Granulibacter bethesdensis*	−4.23 ± 0.06	−4.75 ± 0.06	−5.56 ± 0.07	−6.00 ± 0.07	−6.16 ± 0.07	−6.28 ± 0.07	−6.26 ± 0.07	−6.21 ± 0.07	−6.22 ± 0.07	−6.21 ± 0.07	−6.24 ± 0.07	−6.29 ± 0.07	−6.17 ± 0.07
Bacteria	*Haemophilus ducreyi*	−1.70 ± 0.05	−2.14 ± 0.05	−2.78 ± 0.06	−2.94 ± 0.06	−2.90 ± 0.06	−2.93 ± 0.06	−2.92 ± 0.06	−2.87 ± 0.06	−2.80 ± 0.06	−2.91 ± 0.06	−2.90 ± 0.06	−2.86 ± 0.06	−2.96 ± 0.06
Bacteria	*Hahella chejuensis*	−2.91 ± 0.03	−3.55 ± 0.04	−4.51 ± 0.04	−4.90 ± 0.04	−5.10 ± 0.04	−5.19 ± 0.04	−5.16 ± 0.04	−5.10 ± 0.04	−5.10 ± 0.04	−5.07 ± 0.04	−5.02 ± 0.04	−5.09 ± 0.04	−4.97 ± 0.04
Bacteria	*Halorhodospira halophila*	−4.88 ± 0.06	−5.86 ± 0.07	−7.19 ± 0.07	−7.86 ± 0.08	−8.21 ± 0.08	−8.37 ± 0.08	−8.11 ± 0.07	−7.92 ± 0.07	−8.02 ± 0.07	−7.74 ± 0.07	−7.92 ± 0.07	−7.89 ± 0.07	−7.78 ± 0.07

Continued on next page

Type	Species	Window 1	Window 2	Window 3	Window 4	Window 5	Window 6	Window 7	Window 8	Window 9	Window 10	Window 11	Window 12	Window 13
Bacteria	*Halothermothrix orenii*	−1.87 ± 0.04	−2.15 ± 0.05	−2.51 ± 0.05	−2.57 ± 0.05	−2.81 ± 0.06	−2.82 ± 0.05	−2.78 ± 0.05	−2.79 ± 0.05	−2.81 ± 0.05	−2.95 ± 0.05	−3.03 ± 0.06	−2.99 ± 0.06	−2.92 ± 0.05
Bacteria	*Helicobacter acinonychis*	−1.84 ± 0.05	−2.04 ± 0.05	−2.54 ± 0.06	−2.81 ± 0.06	−2.80 ± 0.06	−2.78 ± 0.06	−2.67 ± 0.06	−2.67 ± 0.06	−2.77 ± 0.06	−2.72 ± 0.06	−2.72 ± 0.06	−2.66 ± 0.06	−2.64 ± 0.06
Bacteria	*Heliobacterium modesticaldum*	−3.93 ± 0.05	−4.55 ± 0.06	−5.12 ± 0.06	−5.12 ± 0.06	−5.41 ± 0.06	−5.48 ± 0.06	−5.46 ± 0.06	−5.54 ± 0.06	−5.61 ± 0.06	−5.57 ± 0.06	−5.53 ± 0.06	−5.51 ± 0.06	−5.40 ± 0.06
Bacteria	*Herminiimonas arsenicoxydans*	−2.70 ± 0.04	−3.51 ± 0.05	−4.57 ± 0.06	−5.17 ± 0.06	−5.30 ± 0.06	−5.52 ± 0.06	−5.42 ± 0.05	−5.36 ± 0.06	−5.42 ± 0.05	−5.43 ± 0.06	−5.38 ± 0.06	−5.42 ± 0.05	−5.32 ± 0.05
Bacteria	*Herpetosiphon aurantiacus*	−3.06 ± 0.03	−3.50 ± 0.04	−4.23 ± 0.04	−4.60 ± 0.04	−4.72 ± 0.04	−4.75 ± 0.04	−4.72 ± 0.04	−4.61 ± 0.04	−4.62 ± 0.04	−4.64 ± 0.04	−4.61 ± 0.04	−4.65 ± 0.04	−4.66 ± 0.04
Bacteria	*Hydrogenobaculum sp. Y04AAS1*	−1.86 ± 0.04	−2.05 ± 0.05	−2.41 ± 0.06	−2.57 ± 0.06	−2.69 ± 0.06	−2.61 ± 0.06	−2.58 ± 0.06	−2.40 ± 0.06	−2.45 ± 0.05	−2.42 ± 0.06	−2.35 ± 0.06	−2.31 ± 0.06	−2.34 ± 0.05
Bacteria	*Hyphomonas neptunium*	−3.75 ± 0.05	−4.68 ± 0.05	−6.17 ± 0.06	−6.84 ± 0.06	−6.94 ± 0.06	−6.93 ± 0.06	−6.83 ± 0.06	−6.71 ± 0.06	−6.74 ± 0.06	−6.63 ± 0.06	−6.62 ± 0.06	−6.68 ± 0.06	−6.63 ± 0.06
Bacteria	*Idiomarina loihiensis*	−2.46 ± 0.04	−3.00 ± 0.05	−3.97 ± 0.06	−4.48 ± 0.06	−4.43 ± 0.06	−4.32 ± 0.06	−4.21 ± 0.05	−4.09 ± 0.05	−4.12 ± 0.05	−4.21 ± 0.05	−4.20 ± 0.05	−4.13 ± 0.05	−4.15 ± 0.05
Bacteria	*Jannaschia sp. CCS1*	−3.59 ± 0.04	−4.55 ± 0.05	−5.98 ± 0.05	−6.66 ± 0.05	−6.82 ± 0.05	−6.80 ± 0.05	−6.60 ± 0.05	−6.46 ± 0.05	−6.54 ± 0.05	−6.47 ± 0.05	−6.55 ± 0.05	−6.56 ± 0.05	−6.51 ± 0.05
Bacteria	*Janthinobacterium sp. Marseille*	−2.56 ± 0.04	−3.46 ± 0.05	−4.64 ± 0.05	−5.33 ± 0.05	−5.40 ± 0.05	−5.50 ± 0.05	−5.41 ± 0.05	−5.39 ± 0.05	−5.42 ± 0.05	−5.31 ± 0.05	−5.32 ± 0.05	−5.45 ± 0.05	−5.35 ± 0.05
Bacteria	*Kineococcus radiotolerans*	−6.38 ± 0.05	−7.41 ± 0.06	−8.55 ± 0.06	−8.75 ± 0.06	−8.95 ± 0.06	−9.03 ± 0.06	−9.01 ± 0.06	−9.01 ± 0.06	−9.15 ± 0.06	−9.00 ± 0.06	−9.07 ± 0.06	−8.96 ± 0.06	−8.85 ± 0.06
Bacteria	*Klebsiella pneumoniae*	−2.81 ± 0.03	−4.01 ± 0.04	−5.38 ± 0.05	−5.72 ± 0.05	−5.83 ± 0.05	−5.89 ± 0.05	−5.80 ± 0.05	−5.73 ± 0.05	−5.79 ± 0.05	−5.85 ± 0.05	−5.77 ± 0.05	−5.92 ± 0.05	−5.81 ± 0.05
Bacteria	*Kocuria rhizophila*	−5.16 ± 0.07	−6.17 ± 0.07	−7.59 ± 0.08	−8.07 ± 0.08	−8.41 ± 0.07	−8.56 ± 0.07	−8.31 ± 0.07	−8.37 ± 0.07	−8.41 ± 0.07	−8.36 ± 0.07	−8.37 ± 0.07	−8.45 ± 0.07	−8.38 ± 0.07
Bacteria	*Kosmotoga olearia*	−2.31 ± 0.04	−2.44 ± 0.05	−2.66 ± 0.05	−2.83 ± 0.05	−2.93 ± 0.05	−2.99 ± 0.05	−3.06 ± 0.05	−3.11 ± 0.06	−3.22 ± 0.06	−3.24 ± 0.05	−3.15 ± 0.05	−3.21 ± 0.06	−3.06 ± 0.05
Bacteria	*Lactobacillus acidophilus*	−1.52 ± 0.04	−1.91 ± 0.05	−2.55 ± 0.06	−2.53 ± 0.06	−2.58 ± 0.05	−2.59 ± 0.05	−2.55 ± 0.05	−2.44 ± 0.05	−2.45 ± 0.05	−2.47 ± 0.05	−2.57 ± 0.05	−2.52 ± 0.05	−2.49 ± 0.05
Bacteria	*Lactococcus lactis*	−1.37 ± 0.03	−1.90 ± 0.04	−2.59 ± 0.05	−2.67 ± 0.05	−2.53 ± 0.05	−2.62 ± 0.05	−2.60 ± 0.05	−2.50 ± 0.04	−2.62 ± 0.05	−2.56 ± 0.05	−2.57 ± 0.05	−2.56 ± 0.04	−2.64 ± 0.05
Bacteria	*Laribacter hongkongensis*	−3.61 ± 0.05	−4.86 ± 0.06	−6.44 ± 0.07	−6.73 ± 0.07	−6.85 ± 0.07	−6.93 ± 0.06	−6.89 ± 0.07	−6.80 ± 0.06	−6.93 ± 0.06	−6.74 ± 0.06	−6.73 ± 0.06	−6.94 ± 0.06	−6.77 ± 0.06
Bacteria	*Lawsonia intracellularis*	−1.78 ± 0.05	−1.78 ± 0.05	−2.21 ± 0.06	−2.43 ± 0.07	−2.36 ± 0.06	−2.30 ± 0.06	−2.25 ± 0.06	−2.10 ± 0.06	−2.26 ± 0.06	−2.31 ± 0.06	−2.24 ± 0.06	−2.32 ± 0.06	−2.38 ± 0.06
Bacteria	*Legionella pneumophila*	−1.75 ± 0.03	−1.99 ± 0.04	−2.46 ± 0.04	−2.73 ± 0.04	−2.84 ± 0.04	−2.83 ± 0.04	−2.80 ± 0.04	−2.72 ± 0.04	−2.74 ± 0.04	−2.76 ± 0.04	−2.75 ± 0.04	−2.79 ± 0.04	−2.79 ± 0.04
Bacteria	*Leifsonia xyli*	−5.53 ± 0.07	−6.40 ± 0.08	−7.39 ± 0.08	−7.42 ± 0.08	−7.63 ± 0.08	−7.56 ± 0.08	−7.61 ± 0.08	−7.51 ± 0.08	−7.56 ± 0.08	−7.52 ± 0.08	−7.65 ± 0.08	−7.72 ± 0.08	−7.52 ± 0.08
Bacteria	*Leptospira biflexa*	−1.66 ± 0.03	−1.76 ± 0.03	−2.25 ± 0.04	−2.78 ± 0.04	−3.03 ± 0.05	−2.98 ± 0.04	−2.83 ± 0.04	−2.80 ± 0.04	−2.78 ± 0.04	−2.83 ± 0.04	−2.85 ± 0.04	−2.85 ± 0.04	−2.94 ± 0.04
Bacteria	*Leptothrix cholodnii*	−3.85 ± 0.05	−5.26 ± 0.05	−7.16 ± 0.06	−7.85 ± 0.06	−8.13 ± 0.06	−8.26 ± 0.06	−8.23 ± 0.05	−8.30 ± 0.05	−8.36 ± 0.05	−8.25 ± 0.05	−8.20 ± 0.05	−8.33 ± 0.05	−8.18 ± 0.05
Bacteria	*Leuconostoc citreum*	−1.68 ± 0.04	−2.04 ± 0.05	−2.84 ± 0.06	−3.00 ± 0.06	−2.92 ± 0.06	−3.00 ± 0.06	−3.00 ± 0.06	−2.92 ± 0.06	−2.93 ± 0.06	−3.13 ± 0.06	−3.02 ± 0.06	−3.13 ± 0.06	−3.00 ± 0.06
Bacteria	*Listeria innocua*	−1.50 ± 0.03	−1.82 ± 0.03	−2.26 ± 0.04	−2.42 ± 0.04	−2.41 ± 0.04	−2.45 ± 0.04	−2.44 ± 0.04	−2.40 ± 0.04	−2.46 ± 0.04	−2.47 ± 0.04	−2.45 ± 0.04	−2.37 ± 0.04	−2.48 ± 0.04
Bacteria	*Lysinibacillus sphaericus*	−1.91 ± 0.03	−2.01 ± 0.03	−2.32 ± 0.03	−2.48 ± 0.03	−2.55 ± 0.03	−2.59 ± 0.03	−2.64 ± 0.03	−2.55 ± 0.03	−2.61 ± 0.03	−2.64 ± 0.03	−2.58 ± 0.03	−2.65 ± 0.03	−2.65 ± 0.03
Bacteria	*Macrococcus caseolyticus*	−1.52 ± 0.04	−1.75 ± 0.04	−2.23 ± 0.05	−2.43 ± 0.05	−2.42 ± 0.05	−2.44 ± 0.05	−2.50 ± 0.05	−2.41 ± 0.05	−2.57 ± 0.05	−2.55 ± 0.05	−2.58 ± 0.05	−2.61 ± 0.05	−2.58 ± 0.05
Bacteria	*Magnetococcus sp. MC-1*	−3.14 ± 0.04	−3.71 ± 0.05	−4.58 ± 0.05	−5.15 ± 0.05	−5.37 ± 0.05	−5.36 ± 0.05	−5.26 ± 0.05	−5.26 ± 0.05	−5.27 ± 0.05	−5.23 ± 0.05	−5.15 ± 0.05	−5.27 ± 0.05	−5.31 ± 0.05
Bacteria	*Magnetospirillum magneticum*	−4.77 ± 0.05	−5.46 ± 0.05	−6.58 ± 0.05	−7.08 ± 0.05	−7.37 ± 0.06	−7.39 ± 0.06	−7.32 ± 0.05	−7.23 ± 0.05	−7.40 ± 0.05	−7.29 ± 0.05	−7.40 ± 0.05	−7.44 ± 0.05	−7.28 ± 0.05
Bacteria	*Mannheimia succiniciproducens*	−1.77 ± 0.04	−2.28 ± 0.05	−3.09 ± 0.06	−3.34 ± 0.06	−3.51 ± 0.06	−3.53 ± 0.06	−3.50 ± 0.06	−3.43 ± 0.06	−3.43 ± 0.06	−3.54 ± 0.06	−3.55 ± 0.06	−3.60 ± 0.06	−3.57 ± 0.06
Bacteria	*Maricaulis maris*	−3.78 ± 0.05	−4.72 ± 0.06	−6.21 ± 0.06	−6.76 ± 0.06	−7.02 ± 0.06	−7.06 ± 0.06	−6.90 ± 0.06	−6.87 ± 0.06	−6.94 ± 0.06	−6.85 ± 0.06	−6.86 ± 0.06	−6.84 ± 0.06	−6.77 ± 0.06
Bacteria	*Marinobacter aquaeolei*	−3.28 ± 0.04	−4.04 ± 0.05	−5.17 ± 0.05	−5.66 ± 0.05	−5.82 ± 0.05	−5.89 ± 0.05	−5.87 ± 0.05	−5.81 ± 0.05	−5.75 ± 0.05	−5.75 ± 0.05	−5.79 ± 0.05	−5.71 ± 0.05	−5.74 ± 0.05
Bacteria	*Marinomonas sp. MWYL1*	−1.94 ± 0.03	−2.42 ± 0.03	−3.21 ± 0.04	−3.62 ± 0.04	−3.59 ± 0.04	−3.65 ± 0.04	−3.52 ± 0.04	−3.48 ± 0.04	−3.43 ± 0.04	−3.46 ± 0.04	−3.43 ± 0.04	−3.50 ± 0.04	−3.49 ± 0.04
Bacteria	*Mesoplasma florum*	−0.93 ± 0.11	−1.29 ± 0.13	−1.97 ± 0.18	−1.92 ± 0.18	−1.91 ± 0.20	−1.83 ± 0.18	−1.86 ± 0.19	−1.89 ± 0.19	−2.07 ± 0.18	−1.95 ± 0.18	−1.94 ± 0.18	−2.05 ± 0.17	−2.00 ± 0.18
Bacteria	*Mesorhizobium loti*	−4.54 ± 0.04	−5.15 ± 0.04	−6.26 ± 0.04	−6.77 ± 0.04	−6.92 ± 0.04	−7.03 ± 0.04	−6.92 ± 0.04	−6.87 ± 0.04	−6.86 ± 0.04	−6.79 ± 0.04	−6.83 ± 0.04	−6.79 ± 0.04	−6.75 ± 0.04
Bacteria	*Methylacidiphilum infernorum*	−2.51 ± 0.05	−2.65 ± 0.05	−3.01 ± 0.06	−3.21 ± 0.06	−3.36 ± 0.06	−3.44 ± 0.06	−3.42 ± 0.06	−3.52 ± 0.06	−3.47 ± 0.06	−3.53 ± 0.06	−3.53 ± 0.06	−3.54 ± 0.06	−3.51 ± 0.06
Bacteria	*Methylibium petroleiphilum*	−4.61 ± 0.05	−5.82 ± 0.06	−7.48 ± 0.06	−8.09 ± 0.06	−8.38 ± 0.06	−8.44 ± 0.06	−8.43 ± 0.06	−8.44 ± 0.06	−8.45 ± 0.06	−8.30 ± 0.06	−8.16 ± 0.06	−8.31 ± 0.06	−8.14 ± 0.06
Bacteria	*Methylobacillus flagellatus*	−2.97 ± 0.05	−3.77 ± 0.05	−4.90 ± 0.06	−5.39 ± 0.06	−5.62 ± 0.06	−5.62 ± 0.06	−5.50 ± 0.06	−5.51 ± 0.06	−5.51 ± 0.06	−5.39 ± 0.06	−5.46 ± 0.06	−5.36 ± 0.06	−5.32 ± 0.06
Bacteria	*Methylobacterium chloromethanicum*	−4.92 ± 0.04	−5.94 ± 0.05	−7.39 ± 0.05	−7.93 ± 0.05	−8.15 ± 0.05	−8.27 ± 0.05	−8.12 ± 0.05	−8.11 ± 0.05	−8.10 ± 0.05	−7.95 ± 0.05	−7.90 ± 0.05	−7.99 ± 0.05	−7.83 ± 0.05
Bacteria	*Methylocella silvestris*	−4.33 ± 0.05	−5.32 ± 0.05	−6.62 ± 0.06	−7.14 ± 0.06	−7.28 ± 0.06	−7.42 ± 0.06	−7.40 ± 0.06	−7.19 ± 0.06	−7.06 ± 0.06	−6.96 ± 0.06	−6.95 ± 0.06	−7.04 ± 0.06	−6.97 ± 0.06

Continued on next page

Type	Species	Window 1	Window 2	Window 3	Window 4	Window 5	Window 6	Window 7	Window 8	Window 9	Window 10	Window 11	Window 12	Window 13
Bacteria	*Methylococcus capsulatus*	-4.20 ± 0.05	-5.09 ± 0.06	-6.30 ± 0.07	-6.68 ± 0.06	-6.80 ± 0.07	-6.97 ± 0.07	-6.82 ± 0.06	-6.78 ± 0.06	-6.82 ± 0.06	-6.78 ± 0.06	-6.76 ± 0.06	-6.80 ± 0.06	-6.80 ± 0.06
Bacteria	*Micrococcus luteus*	-4.89 ± 0.07	-6.28 ± 0.08	-7.98 ± 0.08	-8.46 ± 0.08	-8.83 ± 0.08	-8.95 ± 0.08	-8.75 ± 0.08	-8.74 ± 0.08	-8.84 ± 0.08	-8.82 ± 0.08	-8.82 ± 0.08	-8.89 ± 0.08	-8.65 ± 0.07
Bacteria	*Microcystis aeruginosa*	-2.18 ± 0.03	-2.31 ± 0.03	-2.67 ± 0.03	-3.07 ± 0.03	-3.29 ± 0.03	-3.29 ± 0.03	-3.16 ± 0.03	-3.11 ± 0.03	-3.06 ± 0.03	-3.10 ± 0.03	-3.01 ± 0.03	-3.03 ± 0.03	-3.07 ± 0.03
Bacteria	*Moorella thermoacetica*	-3.89 ± 0.05	-4.47 ± 0.06	-5.30 ± 0.07	-5.52 ± 0.07	-5.69 ± 0.07	-5.82 ± 0.07	-5.72 ± 0.07	-5.66 ± 0.07	-5.84 ± 0.07	-5.77 ± 0.07	-5.75 ± 0.07	-5.67 ± 0.07	-5.67 ± 0.07
Bacteria	*Mycobacterium avium*	-6.06 ± 0.05	-6.98 ± 0.05	-7.87 ± 0.05	-8.06 ± 0.05	-8.18 ± 0.05	-8.25 ± 0.05	-8.14 ± 0.05	-8.09 ± 0.05	-8.21 ± 0.05	-8.02 ± 0.05	-8.01 ± 0.05	-8.06 ± 0.05	-7.95 ± 0.05
Bacteria	*Mycoplasma agalactiae*	-1.18 ± 0.12	-1.24 ± 0.13	-1.97 ± 0.16	-2.19 ± 0.17	-2.00 ± 0.17	-2.14 ± 0.17	-2.28 ± 0.18	-2.10 ± 0.19	-2.17 ± 0.17	-2.37 ± 0.19	-2.29 ± 0.20	-2.29 ± 0.19	-1.97 ± 0.17
Bacteria	*Myxococcus xanthus*	-5.51 ± 0.04	-5.95 ± 0.04	-6.76 ± 0.04	-7.30 ± 0.04	-7.60 ± 0.04	-7.70 ± 0.04	-7.56 ± 0.04	-7.62 ± 0.04	-7.77 ± 0.04	-7.77 ± 0.04	-7.78 ± 0.04	-7.87 ± 0.04	-7.76 ± 0.04
Bacteria	*Natranaerobius thermophilus*	-1.83 ± 0.03	-1.90 ± 0.04	-2.15 ± 0.04	-2.29 ± 0.04	-2.39 ± 0.04	-2.52 ± 0.04	-2.60 ± 0.04	-2.44 ± 0.04	-2.45 ± 0.04	-2.56 ± 0.04	-2.51 ± 0.04	-2.53 ± 0.04	-2.47 ± 0.04
Bacteria	*Nautilia profundicola*	-1.41 ± 0.04	-1.65 ± 0.05	-2.16 ± 0.06	-2.34 ± 0.06	-2.21 ± 0.06	-2.20 ± 0.06	-2.16 ± 0.05	-2.15 ± 0.05	-2.12 ± 0.05	-2.19 ± 0.05	-2.07 ± 0.05	-2.08 ± 0.05	-2.11 ± 0.05
Bacteria	*Neisseria gonorrhoeae*	-2.52 ± 0.06	-3.17 ± 0.06	-4.17 ± 0.07	-4.64 ± 0.07	-4.84 ± 0.08	-4.87 ± 0.08	-4.77 ± 0.07	-4.82 ± 0.08	-4.84 ± 0.07	-4.77 ± 0.07	-4.82 ± 0.08	-4.82 ± 0.07	-4.84 ± 0.07
Bacteria	*Neorickettsia sennetsu*	-2.36 ± 0.07	-2.65 ± 0.08	-3.06 ± 0.08	-3.29 ± 0.09	-3.40 ± 0.09	-3.38 ± 0.09	-3.35 ± 0.09	-3.24 ± 0.08	-3.15 ± 0.08	-3.16 ± 0.08	-3.25 ± 0.08	-3.23 ± 0.08	-3.26 ± 0.08
Bacteria	*Nitratiruptor sp. SB155-2*	-1.99 ± 0.04	-2.27 ± 0.05	-2.77 ± 0.06	-2.91 ± 0.06	-2.88 ± 0.06	-2.94 ± 0.06	-2.80 ± 0.06	-2.71 ± 0.05	-2.79 ± 0.06	-2.72 ± 0.05	-2.81 ± 0.05	-2.82 ± 0.06	-2.75 ± 0.05
Bacteria	*Nitrobacter hamburgensis*	-4.33 ± 0.05	-4.93 ± 0.05	-5.94 ± 0.05	-6.51 ± 0.06	-6.81 ± 0.06	-6.96 ± 0.06	-6.82 ± 0.05	-6.67 ± 0.05	-6.68 ± 0.05	-6.73 ± 0.05	-6.64 ± 0.05	-6.75 ± 0.05	-6.67 ± 0.05
Bacteria	*Nitrosococcus oceani*	-2.77 ± 0.04	-3.24 ± 0.05	-4.05 ± 0.06	-4.45 ± 0.06	-4.59 ± 0.05	-4.74 ± 0.06	-4.66 ± 0.06	-4.68 ± 0.06	-4.55 ± 0.05	-4.55 ± 0.05	-4.58 ± 0.06	-4.52 ± 0.05	-4.54 ± 0.05
Bacteria	*Nitrosomonas europaea*	-2.75 ± 0.04	-3.32 ± 0.05	-4.24 ± 0.06	-4.58 ± 0.06	-4.71 ± 0.06	-4.68 ± 0.06	-4.65 ± 0.06	-4.57 ± 0.06	-4.64 ± 0.06	-4.55 ± 0.06	-4.67 ± 0.06	-4.65 ± 0.06	-4.64 ± 0.06
Bacteria	*Nitrosospira multiformis*	-3.03 ± 0.05	-3.64 ± 0.05	-4.42 ± 0.06	-4.81 ± 0.06	-5.04 ± 0.06	-5.07 ± 0.06	-5.08 ± 0.06	-4.96 ± 0.06	-5.04 ± 0.06	-5.04 ± 0.06	-5.10 ± 0.06	-5.10 ± 0.06	-4.97 ± 0.06
Bacteria	*Nocardia farcinica*	-5.78 ± 0.04	-6.91 ± 0.05	-7.90 ± 0.05	-8.17 ± 0.05	-8.35 ± 0.05	-8.44 ± 0.05	-8.38 ± 0.05	-8.35 ± 0.05	-8.43 ± 0.05	-8.28 ± 0.05	-8.32 ± 0.05	-8.31 ± 0.05	-8.23 ± 0.05
Bacteria	*Nocardioides sp. JS614*	-6.06 ± 0.05	-7.48 ± 0.05	-8.39 ± 0.05	-8.62 ± 0.05	-8.74 ± 0.05	-8.85 ± 0.05	-8.69 ± 0.05	-8.75 ± 0.05	-8.79 ± 0.05	-8.78 ± 0.05	-8.76 ± 0.05	-8.75 ± 0.05	-8.65 ± 0.05
Bacteria	*Nostoc punctiforme*	-2.05 ± 0.03	-2.27 ± 0.03	-2.78 ± 0.03	-3.17 ± 0.03	-3.27 ± 0.03	-3.35 ± 0.03	-3.27 ± 0.03	-3.22 ± 0.03	-3.25 ± 0.03	-3.35 ± 0.03	-3.20 ± 0.03	-3.18 ± 0.03	-3.27 ± 0.03
Bacteria	*Novosphingobium aromaticivorans*	-4.43 ± 0.05	-5.41 ± 0.06	-6.78 ± 0.06	-7.29 ± 0.06	-7.38 ± 0.06	-7.50 ± 0.06	-7.29 ± 0.06	-7.32 ± 0.06	-7.18 ± 0.06	-7.22 ± 0.06	-7.18 ± 0.06	-7.22 ± 0.06	-7.08 ± 0.06
Bacteria	*Oceanobacillus iheyensis*	-1.64 ± 0.03	-1.79 ± 0.03	-2.12 ± 0.04	-2.22 ± 0.04	-2.24 ± 0.04	-2.26 ± 0.04	-2.30 ± 0.04	-2.25 ± 0.03	-2.33 ± 0.04	-2.44 ± 0.04	-2.37 ± 0.04	-2.36 ± 0.04	-2.40 ± 0.04
Bacteria	*Ochrobactrum anthropi*	-3.33 ± 0.04	-4.02 ± 0.04	-5.14 ± 0.05	-5.70 ± 0.05	-5.85 ± 0.05	-5.90 ± 0.05	-5.75 ± 0.05	-5.83 ± 0.05	-5.72 ± 0.05	-5.64 ± 0.05	-5.62 ± 0.05	-5.71 ± 0.05	-5.79 ± 0.05
Bacteria	*Oenococcus oeni*	-1.65 ± 0.05	-1.90 ± 0.05	-2.46 ± 0.06	-2.77 ± 0.06	-2.87 ± 0.06	-2.83 ± 0.06	-2.78 ± 0.06	-2.84 ± 0.06	-2.87 ± 0.06	-2.96 ± 0.06	-2.99 ± 0.06	-2.96 ± 0.06	-2.94 ± 0.06
Bacteria	*Oligotropha carboxidovorans*	-4.24 ± 0.05	-4.95 ± 0.05	-6.08 ± 0.06	-6.79 ± 0.06	-7.05 ± 0.06	-7.06 ± 0.06	-6.96 ± 0.06	-6.83 ± 0.06	-6.80 ± 0.06	-6.87 ± 0.06	-6.77 ± 0.06	-6.89 ± 0.06	-6.75 ± 0.05
Bacteria	*Onion yellows*	-1.07 ± 0.05	-1.27 ± 0.06	-1.46 ± 0.07	-1.43 ± 0.07	-1.43 ± 0.07	-1.37 ± 0.06	-1.44 ± 0.07	-1.35 ± 0.07	-1.41 ± 0.07	-1.51 ± 0.06	-1.60 ± 0.07	-1.52 ± 0.07	-1.53 ± 0.07
Bacteria	*Opitutus terrae*	-3.55 ± 0.04	-4.57 ± 0.05	-6.09 ± 0.05	-7.11 ± 0.05	-7.38 ± 0.05	-7.42 ± 0.05	-7.31 ± 0.05	-7.34 ± 0.05	-7.35 ± 0.05	-7.22 ± 0.05	-7.20 ± 0.05	-7.17 ± 0.05	-7.01 ± 0.05
Bacteria	*Orientia tsutsugamushi*	-1.70 ± 0.05	-1.77 ± 0.06	-2.04 ± 0.06	-2.07 ± 0.06	-2.07 ± 0.06	-2.13 ± 0.06	-2.06 ± 0.06	-1.98 ± 0.06	-1.97 ± 0.06	-2.08 ± 0.06	-2.05 ± 0.06	-2.23 ± 0.06	-2.30 ± 0.06
Bacteria	*Parabacteroides distasonis*	-1.55 ± 0.03	-2.36 ± 0.04	-3.69 ± 0.05	-4.30 ± 0.05	-4.11 ± 0.05	-3.92 ± 0.05	-3.77 ± 0.04	-3.77 ± 0.04	-3.86 ± 0.04	-3.80 ± 0.04	-3.82 ± 0.04	-3.86 ± 0.04	-3.82 ± 0.04
Bacteria	*Paracoccus denitrificans*	-4.41 ± 0.04	-5.57 ± 0.05	-6.95 ± 0.05	-7.62 ± 0.06	-7.72 ± 0.05	-7.78 ± 0.05	-7.73 ± 0.05	-7.64 ± 0.05	-7.69 ± 0.05	-7.71 ± 0.05	-7.70 ± 0.05	-7.78 ± 0.05	-7.66 ± 0.05
Bacteria	*Parvibaculum lavamentivorans*	-4.08 ± 0.05	-4.95 ± 0.05	-6.24 ± 0.06	-6.87 ± 0.06	-7.09 ± 0.06	-7.04 ± 0.06	-6.88 ± 0.06	-6.89 ± 0.06	-6.88 ± 0.06	-6.79 ± 0.06	-6.76 ± 0.06	-6.77 ± 0.06	-6.67 ± 0.06
Bacteria	*Pasteurella multocida*	-1.60 ± 0.04	-2.08 ± 0.05	-2.87 ± 0.06	-3.20 ± 0.06	-3.26 ± 0.06	-3.12 ± 0.06	-3.08 ± 0.05	-2.98 ± 0.05	-3.04 ± 0.05	-3.08 ± 0.05	-2.99 ± 0.05	-3.12 ± 0.05	-3.04 ± 0.05
Bacteria	*Pectobacterium atrosepticum*	-2.59 ± 0.03	-3.35 ± 0.04	-4.36 ± 0.05	-4.68 ± 0.05	-4.75 ± 0.05	-4.72 ± 0.04	-4.71 ± 0.05	-4.62 ± 0.04	-4.66 ± 0.04	-4.70 ± 0.04	-4.66 ± 0.04	-4.66 ± 0.04	-4.69 ± 0.04
Bacteria	*Pediococcus pentosaceus*	-1.55 ± 0.04	-1.93 ± 0.05	-2.61 ± 0.06	-2.66 ± 0.06	-2.69 ± 0.06	-2.82 ± 0.06	-2.82 ± 0.05	-2.68 ± 0.05	-2.66 ± 0.05	-2.80 ± 0.05	-2.77 ± 0.06	-2.74 ± 0.06	-2.69 ± 0.05
Bacteria	*Pelobacter carbinolicus*	-2.95 ± 0.04	-3.63 ± 0.05	-4.50 ± 0.05	-4.99 ± 0.06	-5.27 ± 0.06	-5.40 ± 0.06	-5.29 ± 0.05	-5.20 ± 0.05	-5.34 ± 0.06	-5.31 ± 0.05	-5.34 ± 0.06	-5.37 ± 0.05	-5.33 ± 0.05
Bacteria	*Pelodictyon luteolum*	-2.96 ± 0.05	-3.80 ± 0.06	-4.97 ± 0.07	-5.44 ± 0.07	-5.40 ± 0.07	-5.40 ± 0.07	-5.52 ± 0.07	-5.42 ± 0.07	-5.42 ± 0.07	-5.46 ± 0.07	-5.46 ± 0.07	-5.54 ± 0.07	-5.51 ± 0.07
Bacteria	*Pelotomaculum thermopropionicum*	-3.52 ± 0.05	-3.97 ± 0.06	-4.62 ± 0.06	-4.87 ± 0.06	-5.03 ± 0.07	-5.33 ± 0.07	-5.20 ± 0.06	-5.20 ± 0.06	-5.25 ± 0.06	-5.24 ± 0.06	-5.16 ± 0.06	-5.28 ± 0.06	-5.19 ± 0.06
Bacteria	*Persephonella marina*	-1.96 ± 0.04	-2.08 ± 0.05	-2.46 ± 0.06	-2.54 ± 0.05	-2.68 ± 0.06	-2.73 ± 0.05	-2.61 ± 0.05	-2.62 ± 0.05	-2.64 ± 0.05	-2.63 ± 0.05	-2.58 ± 0.05	-2.59 ± 0.05	-2.63 ± 0.05
Bacteria	*Petrotoga mobilis*	-1.59 ± 0.04	-1.65 ± 0.04	-1.92 ± 0.05	-1.96 ± 0.05	-2.03 ± 0.05	-2.06 ± 0.05	-2.09 ± 0.05	-2.12 ± 0.05	-2.19 ± 0.05	-2.28 ± 0.05	-2.22 ± 0.05	-2.11 ± 0.05	-2.15 ± 0.05
Bacteria	*Phenylobacterium zucineum*	-5.69 ± 0.05	-6.80 ± 0.06	-8.27 ± 0.06	-8.83 ± 0.06	-8.93 ± 0.07	-8.88 ± 0.06	-8.78 ± 0.06	-8.76 ± 0.06	-8.69 ± 0.06	-8.60 ± 0.06	-8.62 ± 0.06	-8.70 ± 0.06	-8.45 ± 0.06

Continued on next page

Type	Species	Window 1	Window 2	Window 3	Window 4	Window 5	Window 6	Window 7	Window 8	Window 9	Window 10	Window 11	Window 12	Window 13
Bacteria	*Photobacterium profundum*	-2.17 ± 0.03	-2.50 ± 0.03	-3.09 ± 0.04	-3.26 ± 0.04	-3.35 ± 0.04	-3.33 ± 0.04	-3.22 ± 0.03	-3.22 ± 0.03	-3.26 ± 0.03	-3.32 ± 0.03	-3.31 ± 0.03	-3.30 ± 0.03	-3.35 ± 0.03
Bacteria	*Photorhabdus luminescens*	-2.14 ± 0.03	-2.63 ± 0.04	-3.33 ± 0.04	-3.51 ± 0.04	-3.55 ± 0.04	-3.68 ± 0.04	-3.59 ± 0.04	-3.52 ± 0.04	-3.53 ± 0.04	-3.58 ± 0.04	-3.48 ± 0.04	-3.44 ± 0.04	-3.47 ± 0.04
Bacteria	*Polaromonas naphthalenivorans*	-2.95 ± 0.04	-4.12 ± 0.05	-5.79 ± 0.06	-6.52 ± 0.06	-6.88 ± 0.06	-6.94 ± 0.06	-6.89 ± 0.06	-6.92 ± 0.06	-7.04 ± 0.06	-6.97 ± 0.06	-7.01 ± 0.06	-7.05 ± 0.05	-6.99 ± 0.06
Bacteria	*Polynucleobacter necessarius*	-1.92 ± 0.04	-2.39 ± 0.05	-3.39 ± 0.06	-4.35 ± 0.07	-4.46 ± 0.06	-4.31 ± 0.06	-4.12 ± 0.06	-4.06 ± 0.06	-4.08 ± 0.06	-3.88 ± 0.06	-3.85 ± 0.06	-3.99 ± 0.06	-4.01 ± 0.06
Bacteria	*Porphyromonas gingivalis*	-2.27 ± 0.05	-3.00 ± 0.06	-4.18 ± 0.07	-4.36 ± 0.07	-4.39 ± 0.07	-4.34 ± 0.06	-4.20 ± 0.07	-4.08 ± 0.07	-4.32 ± 0.07	-4.12 ± 0.06	-4.23 ± 0.06	-4.36 ± 0.07	-4.39 ± 0.06
Bacteria	*Prochlorococcus marinus*	-1.51 ± 0.04	-1.55 ± 0.04	-2.03 ± 0.05	-2.29 ± 0.05	-2.17 ± 0.05	-2.14 ± 0.05	-2.11 ± 0.05	-1.95 ± 0.05	-2.03 ± 0.05	-2.11 ± 0.05	-2.04 ± 0.05	-2.09 ± 0.05	-2.09 ± 0.05
Bacteria	*Propionibacterium acnes*	-4.43 ± 0.06	-5.18 ± 0.06	-5.92 ± 0.07	-6.03 ± 0.07	-6.08 ± 0.07	-6.04 ± 0.07	-5.98 ± 0.07	-5.93 ± 0.07	-5.99 ± 0.06	-5.99 ± 0.07	-5.95 ± 0.07	-5.94 ± 0.07	-5.96 ± 0.06
Bacteria	*Prosthecochloris aestuarii*	-2.37 ± 0.04	-3.06 ± 0.05	-3.95 ± 0.06	-4.39 ± 0.06	-4.62 ± 0.06	-4.42 ± 0.06	-4.42 ± 0.06	-4.34 ± 0.06	-4.46 ± 0.06	-4.38 ± 0.06	-4.40 ± 0.06	-4.46 ± 0.06	-4.35 ± 0.06
Bacteria	*Proteus mirabilis*	-1.89 ± 0.03	-2.41 ± 0.04	-3.07 ± 0.05	-3.24 ± 0.05	-3.23 ± 0.04	-3.29 ± 0.04	-3.19 ± 0.04	-3.05 ± 0.04	-3.09 ± 0.04	-3.19 ± 0.04	-3.08 ± 0.04	-3.04 ± 0.04	-3.02 ± 0.04
Bacteria	*Pseudoalteromonas atlantica*	-1.99 ± 0.03	-2.51 ± 0.03	-3.35 ± 0.04	-3.71 ± 0.04	-3.74 ± 0.04	-3.77 ± 0.04	-3.68 ± 0.04	-3.65 ± 0.04	-3.69 ± 0.04	-3.68 ± 0.04	-3.74 ± 0.04	-3.70 ± 0.04	-3.68 ± 0.04
Bacteria	*Pseudomonas aeruginosa*	-3.93 ± 0.04	-5.09 ± 0.04	-6.58 ± 0.05	-7.09 ± 0.05	-7.28 ± 0.05	-7.38 ± 0.05	-7.36 ± 0.05	-7.45 ± 0.05	-7.51 ± 0.05	-7.38 ± 0.05	-7.46 ± 0.05	-7.43 ± 0.05	-7.37 ± 0.04
Bacteria	*Psychrobacter arcticus*	-1.74 ± 0.04	-2.17 ± 0.05	-2.94 ± 0.05	-3.32 ± 0.06	-3.54 ± 0.06	-3.65 ± 0.06	-3.67 ± 0.06	-3.71 ± 0.06	-3.71 ± 0.06	-3.66 ± 0.06	-3.83 ± 0.06	-3.82 ± 0.06	-3.80 ± 0.06
Bacteria	*Psychromonas ingrahamii*	-1.52 ± 0.03	-1.98 ± 0.03	-2.71 ± 0.04	-3.02 ± 0.04	-3.10 ± 0.04	-3.08 ± 0.04	-3.09 ± 0.04	-3.08 ± 0.04	-3.07 ± 0.04	-3.11 ± 0.04	-3.15 ± 0.04	-3.12 ± 0.04	-3.13 ± 0.04
Bacteria	*Ralstonia solanacearum*	-4.14 ± 0.05	-5.35 ± 0.06	-6.77 ± 0.06	-7.50 ± 0.07	-7.67 ± 0.06	-7.93 ± 0.06	-7.87 ± 0.06	-7.77 ± 0.06	-7.89 ± 0.06	-7.83 ± 0.06	-7.71 ± 0.06	-7.77 ± 0.06	-7.58 ± 0.06
Bacteria	*Renibacterium salmoninarum*	-4.36 ± 0.05	-4.74 ± 0.05	-5.26 ± 0.05	-5.72 ± 0.06	-5.56 ± 0.05	-5.59 ± 0.05	-5.68 ± 0.05	-5.65 ± 0.05	-5.67 ± 0.05	-5.74 ± 0.05	-5.66 ± 0.05	-5.69 ± 0.05	-5.63 ± 0.05
Bacteria	*Rhizobium etli*	-3.95 ± 0.04	-4.77 ± 0.05	-5.89 ± 0.05	-6.28 ± 0.05	-6.49 ± 0.05	-6.55 ± 0.05	-6.42 ± 0.05	-6.40 ± 0.05	-6.54 ± 0.05	-6.44 ± 0.05	-6.47 ± 0.05	-6.45 ± 0.05	-6.35 ± 0.05
Bacteria	*Rhodobacter sphaeroides*	-4.66 ± 0.05	-5.92 ± 0.06	-7.38 ± 0.07	-7.84 ± 0.07	-8.06 ± 0.06	-8.10 ± 0.07	-7.92 ± 0.06	-8.02 ± 0.06	-8.07 ± 0.06	-7.92 ± 0.06	-7.98 ± 0.06	-7.95 ± 0.06	-8.01 ± 0.06
Bacteria	*Rhodococcus erythropolis*	-4.51 ± 0.04	-5.17 ± 0.04	-6.14 ± 0.04	-6.47 ± 0.04	-6.54 ± 0.04	-6.59 ± 0.04	-6.56 ± 0.04	-6.52 ± 0.04	-6.52 ± 0.04	-6.45 ± 0.04	-6.51 ± 0.04	-6.50 ± 0.04	-6.50 ± 0.04
Bacteria	*Rhodoferax ferrireducens*	-2.85 ± 0.04	-3.94 ± 0.05	-5.60 ± 0.05	-6.22 ± 0.05	-6.38 ± 0.05	-6.49 ± 0.05	-6.44 ± 0.05	-6.42 ± 0.05	-6.47 ± 0.05	-6.41 ± 0.05	-6.51 ± 0.05	-6.53 ± 0.05	-6.35 ± 0.05
Bacteria	*Rhodopirellula baltica*	-3.95 ± 0.03	-4.12 ± 0.04	-4.51 ± 0.04	-4.78 ± 0.04	-4.96 ± 0.04	-5.03 ± 0.04	-5.07 ± 0.04	-5.16 ± 0.04	-5.13 ± 0.04	-5.04 ± 0.04	-5.14 ± 0.04	-5.19 ± 0.04	-5.23 ± 0.04
Bacteria	*Rhodopseudomonas palustris*	-4.35 ± 0.04	-5.16 ± 0.04	-6.47 ± 0.05	-7.28 ± 0.05	-7.51 ± 0.05	-7.64 ± 0.05	-7.50 ± 0.05	-7.44 ± 0.05	-7.43 ± 0.05	-7.29 ± 0.05	-7.30 ± 0.05	-7.31 ± 0.05	-7.24 ± 0.05
Bacteria	*Rhodospirillum centenum*	-5.21 ± 0.05	-6.31 ± 0.06	-7.71 ± 0.06	-8.26 ± 0.06	-8.64 ± 0.06	-8.70 ± 0.06	-8.59 ± 0.06	-8.66 ± 0.06	-8.66 ± 0.06	-8.48 ± 0.06	-8.55 ± 0.06	-8.50 ± 0.06	-8.40 ± 0.06
Bacteria	*Rickettsia akari*	-1.42 ± 0.05	-1.49 ± 0.05	-1.94 ± 0.06	-2.07 ± 0.06	-2.20 ± 0.07	-2.18 ± 0.07	-2.10 ± 0.06	-2.08 ± 0.06	-2.05 ± 0.06	-2.08 ± 0.06	-2.13 ± 0.06	-2.04 ± 0.06	-2.14 ± 0.06
Bacteria	*Roseiflexus castenholzii*	-3.86 ± 0.04	-4.50 ± 0.04	-5.55 ± 0.05	-6.08 ± 0.05	-6.23 ± 0.05	-6.36 ± 0.05	-6.37 ± 0.05	-6.35 ± 0.05	-6.36 ± 0.05	-6.23 ± 0.05	-6.18 ± 0.05	-6.25 ± 0.05	-6.22 ± 0.05
Bacteria	*Roseobacter denitrificans*	-3.41 ± 0.04	-4.29 ± 0.05	-5.58 ± 0.05	-6.17 ± 0.05	-6.18 ± 0.05	-6.21 ± 0.05	-6.08 ± 0.05	-6.02 ± 0.05	-6.05 ± 0.05	-6.10 ± 0.05	-6.06 ± 0.05	-6.02 ± 0.05	-6.02 ± 0.05
Bacteria	*Rubrobacter xylanophilus*	-6.54 ± 0.06	-7.18 ± 0.06	-8.07 ± 0.07	-8.52 ± 0.07	-8.85 ± 0.07	-8.76 ± 0.07	-8.76 ± 0.07	-8.67 ± 0.07	-8.70 ± 0.07	-8.54 ± 0.07	-8.52 ± 0.07	-8.47 ± 0.07	-8.35 ± 0.07
Bacteria	*Ruegeria pomeroyi*	-4.08 ± 0.05	-5.03 ± 0.05	-6.46 ± 0.06	-7.08 ± 0.06	-7.29 ± 0.06	-7.38 ± 0.06	-7.17 ± 0.06	-7.27 ± 0.06	-7.30 ± 0.06	-7.27 ± 0.06	-7.26 ± 0.06	-7.31 ± 0.06	-7.20 ± 0.05
Bacteria	*Saccharophagus degradans*	-2.11 ± 0.03	-2.59 ± 0.04	-3.55 ± 0.05	-4.08 ± 0.05	-4.33 ± 0.05	-4.25 ± 0.05	-4.22 ± 0.04	-4.07 ± 0.04	-4.02 ± 0.04	-4.04 ± 0.05	-3.97 ± 0.04	-4.02 ± 0.04	-4.00 ± 0.04
Bacteria	*Saccharopolyspora erythraea*	-6.51 ± 0.04	-7.59 ± 0.04	-8.50 ± 0.04	-8.62 ± 0.04	-8.71 ± 0.04	-8.77 ± 0.04	-8.70 ± 0.04	-8.74 ± 0.04	-8.80 ± 0.04	-8.60 ± 0.04	-8.60 ± 0.04	-8.60 ± 0.04	-8.54 ± 0.04
Bacteria	*Salinibacter ruber*	-4.67 ± 0.06	-5.11 ± 0.06	-5.89 ± 0.07	-6.33 ± 0.07	-6.64 ± 0.07	-6.70 ± 0.07	-6.64 ± 0.07	-6.64 ± 0.07	-6.83 ± 0.07	-6.81 ± 0.07	-6.87 ± 0.07	-6.92 ± 0.07	-6.88 ± 0.07
Bacteria	*Salinispora arenicola*	-5.95 ± 0.04	-7.05 ± 0.05	-8.06 ± 0.05	-8.20 ± 0.05	-8.18 ± 0.05	-8.28 ± 0.05	-8.15 ± 0.05	-8.14 ± 0.05	-8.32 ± 0.05	-8.14 ± 0.05	-8.12 ± 0.05	-8.17 ± 0.05	-8.10 ± 0.05
Bacteria	*Salmonella enterica*	-2.94 ± 0.04	-3.56 ± 0.04	-4.45 ± 0.05	-4.72 ± 0.05	-4.73 ± 0.05	-4.81 ± 0.05	-4.69 ± 0.05	-4.67 ± 0.05	-4.75 ± 0.05	-4.69 ± 0.05	-4.70 ± 0.05	-4.84 ± 0.05	-4.75 ± 0.05
Bacteria	*Serratia proteamaculans*	-2.78 ± 0.03	-3.85 ± 0.04	-5.09 ± 0.05	-5.44 ± 0.05	-5.57 ± 0.05	-5.60 ± 0.05	-5.54 ± 0.05	-5.44 ± 0.05	-5.60 ± 0.05	-5.57 ± 0.05	-5.48 ± 0.05	-5.58 ± 0.05	-5.58 ± 0.05
Bacteria	*Shewanella amazonensis*	-2.77 ± 0.04	-3.76 ± 0.05	-4.88 ± 0.05	-5.23 ± 0.05	-5.42 ± 0.05	-5.27 ± 0.05	-5.09 ± 0.05	-5.04 ± 0.05	-5.11 ± 0.05	-5.12 ± 0.05	-5.12 ± 0.05	-5.18 ± 0.05	-5.09 ± 0.05
Bacteria	*Shigella boydii*	-3.07 ± 0.04	-3.63 ± 0.04	-4.75 ± 0.05	-5.01 ± 0.05	-4.89 ± 0.05	-4.70 ± 0.05	-4.85 ± 0.05	-4.74 ± 0.05	-4.71 ± 0.05	-4.71 ± 0.05	-4.66 ± 0.05	-4.76 ± 0.05	-4.72 ± 0.05
Bacteria	*Sinorhizobium medicae*	-3.83 ± 0.04	-4.72 ± 0.05	-6.05 ± 0.06	-6.59 ± 0.06	-6.71 ± 0.06	-6.75 ± 0.06	-6.60 ± 0.06	-6.57 ± 0.05	-6.59 ± 0.05	-6.52 ± 0.05	-6.43 ± 0.05	-6.50 ± 0.05	-6.43 ± 0.05
Bacteria	*Sodalis glossinidius*	-3.02 ± 0.05	-3.87 ± 0.06	-5.08 ± 0.07	-5.34 ± 0.07	-5.52 ± 0.07	-5.43 ± 0.07	-5.41 ± 0.07	-5.31 ± 0.07	-5.28 ± 0.07	-5.34 ± 0.07	-5.44 ± 0.07	-5.48 ± 0.07	-5.37 ± 0.07
Bacteria	*Solibacter usitatus*	-4.17 ± 0.03	-4.89 ± 0.03	-5.92 ± 0.04	-6.55 ± 0.04	-6.53 ± 0.04	-6.46 ± 0.04	-6.33 ± 0.04	-6.35 ± 0.04	-6.32 ± 0.04	-6.30 ± 0.04	-6.29 ± 0.04	-6.31 ± 0.04	-6.19 ± 0.04
Bacteria	*Sorangium cellulosum*	-5.70 ± 0.03	-6.41 ± 0.04	-7.41 ± 0.04	-7.98 ± 0.04	-8.35 ± 0.04	-8.61 ± 0.04	-8.63 ± 0.04	-8.65 ± 0.04	-8.69 ± 0.04	-8.62 ± 0.04	-8.57 ± 0.04	-8.65 ± 0.04	-8.58 ± 0.04

Continued on next page

Type	Species	Window 1	Window 2	Window 3	Window 4	Window 5	Window 6	Window 7	Window 8	Window 9	Window 10	Window 11	Window 12	Window 13
Bacteria	*Sphingomonas wittichii*	-5.00 ± 0.04	-6.16 ± 0.05	-7.61 ± 0.05	-8.17 ± 0.05	-8.21 ± 0.05	-8.41 ± 0.05	-8.25 ± 0.05	-8.26 ± 0.05	-8.25 ± 0.05	-8.15 ± 0.05	-8.11 ± 0.05	-8.14 ± 0.05	-8.05 ± 0.05
Bacteria	*Sphingopyxis alaskensis*	-4.50 ± 0.05	-5.52 ± 0.06	-6.95 ± 0.07	-7.42 ± 0.07	-7.65 ± 0.06	-7.64 ± 0.06	-7.63 ± 0.06	-7.41 ± 0.06	-7.52 ± 0.06	-7.44 ± 0.06	-7.40 ± 0.06	-7.49 ± 0.06	-7.43 ± 0.06
Bacteria	*Staphylococcus aureus*	-1.52 ± 0.03	-1.66 ± 0.04	-2.02 ± 0.04	-2.19 ± 0.04	-2.14 ± 0.04	-2.21 ± 0.04	-2.11 ± 0.04	-2.07 ± 0.04	-2.16 ± 0.04	-2.17 ± 0.04	-2.12 ± 0.04	-2.09 ± 0.04	-2.14 ± 0.04
Bacteria	*Stenotrophomonas maltophilia*	-4.29 ± 0.05	-5.41 ± 0.05	-6.86 ± 0.06	-7.43 ± 0.06	-7.65 ± 0.06	-7.82 ± 0.05	-7.70 ± 0.05	-7.63 ± 0.05	-7.68 ± 0.05	-7.67 ± 0.05	-7.62 ± 0.05	-7.68 ± 0.05	-7.55 ± 0.05
Bacteria	*Streptococcus agalactiae*	-1.47 ± 0.04	-2.04 ± 0.05	-2.59 ± 0.05	-2.68 ± 0.05	-2.53 ± 0.05	-2.51 ± 0.05	-2.55 ± 0.05	-2.39 ± 0.05	-2.50 ± 0.05	-2.64 ± 0.05	-2.73 ± 0.05	-2.56 ± 0.05	-2.49 ± 0.05
Bacteria	*Streptomyces avermitilis*	-5.71 ± 0.04	-6.64 ± 0.04	-7.58 ± 0.04	-7.91 ± 0.04	-8.06 ± 0.04	-8.31 ± 0.04	-8.25 ± 0.04	-8.20 ± 0.04	-8.27 ± 0.04	-8.16 ± 0.04	-8.14 ± 0.04	-8.21 ± 0.04	-8.07 ± 0.04
Bacteria	*Sulfurihydrogenibium azorense*	-1.58 ± 0.04	-1.69 ± 0.04	-1.87 ± 0.05	-1.94 ± 0.05	-2.00 ± 0.05	-2.05 ± 0.05	-2.04 ± 0.05	-1.94 ± 0.05	-2.01 ± 0.05	-1.99 ± 0.05	-1.93 ± 0.05	-1.97 ± 0.05	-1.97 ± 0.05
Bacteria	*Sulfurimonas denitrificans*	-1.38 ± 0.03	-1.72 ± 0.04	-2.24 ± 0.05	-2.48 ± 0.05	-2.54 ± 0.05	-2.52 ± 0.05	-2.49 ± 0.05	-2.40 ± 0.05	-2.43 ± 0.05	-2.54 ± 0.05	-2.44 ± 0.05	-2.45 ± 0.05	-2.42 ± 0.05
Bacteria	*Sulfurovum sp. NBC37-1*	-1.62 ± 0.04	-2.24 ± 0.04	-3.14 ± 0.05	-3.46 ± 0.06	-3.50 ± 0.06	-3.54 ± 0.05	-3.42 ± 0.05	-3.30 ± 0.05	-3.48 ± 0.05	-3.48 ± 0.05	-3.46 ± 0.05	-3.50 ± 0.05	-3.53 ± 0.05
Bacteria	*Symbiobacterium thermophilum*	-6.05 ± 0.05	-6.86 ± 0.06	-7.52 ± 0.06	-7.73 ± 0.06	-7.86 ± 0.06	-7.92 ± 0.06	-7.91 ± 0.06	-7.98 ± 0.06	-8.04 ± 0.06	-7.83 ± 0.06	-7.85 ± 0.06	-8.06 ± 0.06	-7.96 ± 0.06
Bacteria	*Synechococcus elongatus*	-3.92 ± 0.05	-4.20 ± 0.05	-5.14 ± 0.06	-5.40 ± 0.06	-5.56 ± 0.06	-5.48 ± 0.06	-5.44 ± 0.06	-5.40 ± 0.06	-5.49 ± 0.06	-5.48 ± 0.06	-5.42 ± 0.06	-5.45 ± 0.06	-5.37 ± 0.06
Bacteria	*Synechocystis sp. PCC 6803*	-2.42 ± 0.04	-2.65 ± 0.04	-3.32 ± 0.05	-3.85 ± 0.05	-4.10 ± 0.05	-4.19 ± 0.05	-4.20 ± 0.05	-4.22 ± 0.05	-4.17 ± 0.05	-4.07 ± 0.05	-4.03 ± 0.05	-4.06 ± 0.05	-4.02 ± 0.05
Bacteria	*Syntrophobacter fumaroxidans*	-3.88 ± 0.04	-4.44 ± 0.04	-5.16 ± 0.05	-5.66 ± 0.05	-5.93 ± 0.05	-6.02 ± 0.05	-6.02 ± 0.05	-5.99 ± 0.05	-6.07 ± 0.05	-5.96 ± 0.05	-6.03 ± 0.05	-5.98 ± 0.05	-6.03 ± 0.05
Bacteria	*Syntrophomonas wolfei*	-2.44 ± 0.04	-2.80 ± 0.05	-3.30 ± 0.05	-3.40 ± 0.06	-3.60 ± 0.06	-3.74 ± 0.06	-3.79 ± 0.06	-3.72 ± 0.06	-3.79 ± 0.06	-3.80 ± 0.06	-3.75 ± 0.06	-3.78 ± 0.06	-3.75 ± 0.05
Bacteria	*Syntrophus aciditrophicus*	-2.82 ± 0.04	-3.19 ± 0.05	-3.72 ± 0.05	-3.98 ± 0.05	-4.28 ± 0.05	-4.39 ± 0.06	-4.39 ± 0.05	-4.34 ± 0.05	-4.49 ± 0.05	-4.42 ± 0.05	-4.51 ± 0.05	-4.60 ± 0.05	-4.52 ± 0.05
Bacteria	*Thauera sp. MZ1T*	-3.85 ± 0.05	-5.21 ± 0.05	-6.99 ± 0.06	-7.52 ± 0.06	-7.76 ± 0.06	-7.91 ± 0.06	-7.91 ± 0.06	-7.88 ± 0.06	-7.83 ± 0.06	-7.89 ± 0.06	-7.88 ± 0.06	-7.95 ± 0.06	-7.82 ± 0.06
Bacteria	*Thermoanaerobacter pseudethanolicus*	-1.72 ± 0.04	-1.86 ± 0.04	-2.11 ± 0.05	-2.18 ± 0.05	-2.21 ± 0.05	-2.27 ± 0.05	-2.25 ± 0.05	-2.18 ± 0.05	-2.25 ± 0.05	-2.26 ± 0.05	-2.15 ± 0.04	-2.19 ± 0.04	-2.19 ± 0.04
Bacteria	*Thermobifida fusca*	-5.35 ± 0.05	-6.13 ± 0.06	-6.93 ± 0.06	-7.06 ± 0.06	-7.23 ± 0.06	-7.22 ± 0.06	-7.31 ± 0.06	-7.30 ± 0.06	-7.32 ± 0.06	-7.31 ± 0.06	-7.24 ± 0.06	-7.19 ± 0.06	-7.17 ± 0.06
Bacteria	*Thermodesulfovibrio yellowstonii*	-1.65 ± 0.04	-1.71 ± 0.04	-1.96 ± 0.05	-2.19 ± 0.05	-2.40 ± 0.05	-2.38 ± 0.05	-2.33 ± 0.05	-2.23 ± 0.05	-2.32 ± 0.05	-2.31 ± 0.05	-2.22 ± 0.05	-2.28 ± 0.05	-2.17 ± 0.05
Bacteria	*Thermomicrobium roseum*	-5.67 ± 0.06	-5.88 ± 0.07	-6.28 ± 0.07	-6.44 ± 0.07	-6.75 ± 0.08	-6.88 ± 0.07	-6.81 ± 0.07	-6.78 ± 0.08	-6.67 ± 0.07	-6.67 ± 0.07	-6.69 ± 0.07	-6.66 ± 0.07	-6.60 ± 0.08
Bacteria	*Thermosipho africanus*	-1.65 ± 0.04	-1.58 ± 0.04	-1.76 ± 0.05	-1.83 ± 0.05	-1.77 ± 0.05	-1.79 ± 0.04	-1.88 ± 0.05	-1.86 ± 0.04	-1.90 ± 0.05	-1.93 ± 0.05	-1.92 ± 0.05	-2.01 ± 0.05	-2.03 ± 0.05
Bacteria	*Thermosynechococcus elongatus*	-3.42 ± 0.05	-3.68 ± 0.05	-4.29 ± 0.06	-4.78 ± 0.06	-4.95 ± 0.06	-4.94 ± 0.06	-4.80 ± 0.06	-5.02 ± 0.06	-4.91 ± 0.06	-4.95 ± 0.06	-4.86 ± 0.06	-5.02 ± 0.06	-4.83 ± 0.06
Bacteria	*Thermotoga lettingae*	-2.25 ± 0.04	-2.38 ± 0.05	-2.51 ± 0.05	-2.64 ± 0.05	-2.56 ± 0.05	-2.63 ± 0.05	-2.66 ± 0.05	-2.75 ± 0.05	-2.81 ± 0.05	-2.92 ± 0.05	-2.71 ± 0.05	-2.71 ± 0.05	-2.74 ± 0.05
Bacteria	*Thermus thermophilus*	-6.09 ± 0.07	-6.41 ± 0.07	-7.34 ± 0.08	-8.21 ± 0.09	-8.41 ± 0.09	-8.54 ± 0.09	-8.15 ± 0.08	-8.28 ± 0.09	-8.45 ± 0.09	-8.24 ± 0.09	-8.31 ± 0.09	-8.34 ± 0.09	-8.09 ± 0.09
Bacteria	*Thioalkalivibrio sp. HL-EbGR7*	-3.68 ± 0.05	-4.86 ± 0.06	-6.29 ± 0.06	-7.17 ± 0.06	-7.43 ± 0.06	-7.56 ± 0.06	-7.37 ± 0.06	-7.37 ± 0.06	-7.39 ± 0.06	-7.23 ± 0.06	-7.31 ± 0.06	-7.30 ± 0.06	-7.23 ± 0.06
Bacteria	*Thiobacillus denitrificans*	-4.19 ± 0.06	-5.20 ± 0.06	-6.48 ± 0.07	-7.23 ± 0.07	-7.47 ± 0.07	-7.51 ± 0.07	-7.34 ± 0.07	-7.35 ± 0.07	-7.39 ± 0.07	-7.31 ± 0.07	-7.23 ± 0.07	-7.27 ± 0.07	-7.16 ± 0.06
Bacteria	*Thiomicrospira crunogena*	-1.83 ± 0.04	-2.20 ± 0.05	-2.97 ± 0.05	-3.45 ± 0.06	-3.62 ± 0.06	-3.64 ± 0.06	-3.49 ± 0.06	-3.50 ± 0.06	-3.57 ± 0.06	-3.58 ± 0.05	-3.50 ± 0.05	-3.55 ± 0.06	-3.57 ± 0.06
Bacteria	*Tolumonas auensis*	-2.46 ± 0.04	-3.27 ± 0.05	-4.33 ± 0.06	-4.56 ± 0.05	-4.65 ± 0.05	-4.63 ± 0.05	-4.61 ± 0.05	-4.43 ± 0.05	-4.44 ± 0.05	-4.47 ± 0.05	-4.53 ± 0.05	-4.56 ± 0.05	-4.50 ± 0.05
Bacteria	*Treponema denticola*	-1.49 ± 0.03	-1.89 ± 0.04	-2.51 ± 0.05	-2.65 ± 0.05	-2.71 ± 0.05	-2.88 ± 0.05	-2.80 ± 0.05	-2.84 ± 0.05	-2.91 ± 0.05	-2.96 ± 0.05	-2.90 ± 0.05	-2.86 ± 0.05	-2.82 ± 0.05
Bacteria	*Trichodesmium erythraeum*	-1.54 ± 0.03	-1.68 ± 0.03	-2.11 ± 0.03	-2.43 ± 0.04	-2.48 ± 0.04	-2.54 ± 0.03	-2.47 ± 0.03	-2.38 ± 0.03	-2.40 ± 0.03	-2.48 ± 0.03	-2.41 ± 0.03	-2.36 ± 0.03	-2.40 ± 0.03
Bacteria	*Tropheryma whipplei*	-3.33 ± 0.09	-3.55 ± 0.09	-3.95 ± 0.10	-4.05 ± 0.10	-3.98 ± 0.10	-4.07 ± 0.10	-4.07 ± 0.10	-3.87 ± 0.09	-3.97 ± 0.09	-3.98 ± 0.09	-3.94 ± 0.10	-3.97 ± 0.10	-4.02 ± 0.09
Bacteria	*Ureaplasma parvum*	-1.38 ± 0.12	-1.45 ± 0.15	-1.74 ± 0.15	-1.77 ± 0.15	-1.62 ± 0.14	-1.56 ± 0.16	-1.68 ± 0.16	-1.52 ± 0.14	-1.67 ± 0.15	-1.64 ± 0.14	-1.68 ± 0.15	-1.56 ± 0.15	-1.45 ± 0.14
Bacteria	*Variovorax paradoxus*	-3.63 ± 0.04	-5.10 ± 0.04	-6.99 ± 0.05	-7.81 ± 0.05	-7.97 ± 0.05	-8.07 ± 0.05	-8.05 ± 0.05	-7.98 ± 0.04	-8.06 ± 0.05	-7.98 ± 0.04	-7.92 ± 0.04	-8.04 ± 0.04	-7.93 ± 0.04
Bacteria	*Verminephrobacter eiseniae*	-4.11 ± 0.04	-5.22 ± 0.05	-6.76 ± 0.05	-7.30 ± 0.05	-7.48 ± 0.05	-7.51 ± 0.05	-7.41 ± 0.05	-7.45 ± 0.05	-7.57 ± 0.05	-7.40 ± 0.05	-7.47 ± 0.05	-7.52 ± 0.05	-7.41 ± 0.05
Bacteria	*Vibrio cholerae*	-2.48 ± 0.04	-2.91 ± 0.04	-3.62 ± 0.05	-3.98 ± 0.05	-4.09 ± 0.05	-4.10 ± 0.05	-3.90 ± 0.05	-3.93 ± 0.05	-4.14 ± 0.05	-4.07 ± 0.05	-4.05 ± 0.05	-4.16 ± 0.05	-4.14 ± 0.05
Bacteria	*Wigglesworthia glossinidia*	-1.17 ± 0.06	-1.19 ± 0.06	-1.49 ± 0.07	-1.65 ± 0.08	-1.64 ± 0.08	-1.52 ± 0.08	-1.52 ± 0.07	-1.50 ± 0.07	-1.53 ± 0.07	-1.64 ± 0.07	-1.47 ± 0.07	-1.43 ± 0.07	-1.34 ± 0.06
Bacteria	*Wolbachia endosymbiont*	-1.52 ± 0.04	-1.69 ± 0.05	-2.08 ± 0.06	-2.41 ± 0.06	-2.52 ± 0.06	-2.43 ± 0.06	-2.32 ± 0.06	-2.11 ± 0.06	-2.17 ± 0.06	-2.21 ± 0.06	-2.31 ± 0.06	-2.16 ± 0.06	-2.13 ± 0.06
Bacteria	*Wolinella succinogenes*	-2.56 ± 0.05	-3.10 ± 0.06	-3.96 ± 0.07	-4.19 ± 0.07	-4.22 ± 0.07	-4.20 ± 0.06	-3.99 ± 0.06	-4.10 ± 0.06	-4.15 ± 0.06	-4.14 ± 0.06	-4.07 ± 0.06	-4.07 ± 0.06	-4.07 ± 0.06
Bacteria	*Xanthobacter autotrophicus*	-4.79 ± 0.04	-5.71 ± 0.05	-6.98 ± 0.05	-7.51 ± 0.06	-7.75 ± 0.05	-7.81 ± 0.05	-7.77 ± 0.05	-7.69 ± 0.05	-7.74 ± 0.05	-7.68 ± 0.05	-7.62 ± 0.05	-7.75 ± 0.05	-7.71 ± 0.05

Continued on next page

Type	Species	Window 1	Window 2	Window 3	Window 4	Window 5	Window 6	Window 7	Window 8	Window 9	Window 10	Window 11	Window 12	Window 13
Bacteria	*Xanthomonas axonopodis*	-4.52 ± 0.05	-5.34 ± 0.05	-6.58 ± 0.05	-7.15 ± 0.05	-7.36 ± 0.05	-7.44 ± 0.05	-7.37 ± 0.05	-7.41 ± 0.05	-7.49 ± 0.05	-7.36 ± 0.05	-7.40 ± 0.05	-7.37 ± 0.05	-7.35 ± 0.05
Bacteria	*Xylella fastidiosa*	-3.24 ± 0.05	-3.50 ± 0.06	-4.08 ± 0.06	-4.47 ± 0.06	-4.58 ± 0.06	-4.61 ± 0.06	-4.66 ± 0.06	-4.61 ± 0.06	-4.56 ± 0.06	-4.62 ± 0.06	-4.57 ± 0.06	-4.62 ± 0.06	-4.55 ± 0.06
Bacteria	*Yersinia enterocolitica*	-2.51 ± 0.03	-3.21 ± 0.04	-4.11 ± 0.05	-4.37 ± 0.05	-4.32 ± 0.05	-4.41 ± 0.05	-4.33 ± 0.05	-4.22 ± 0.04	-4.24 ± 0.05	-4.25 ± 0.05	-4.29 ± 0.05	-4.24 ± 0.04	-4.21 ± 0.04
Bacteria	*Zymomonas mobilis*	-2.92 ± 0.06	-3.20 ± 0.06	-3.91 ± 0.07	-4.18 ± 0.07	-4.30 ± 0.07	-4.38 ± 0.07	-4.18 ± 0.07	-4.20 ± 0.07	-4.23 ± 0.07	-4.33 ± 0.07	-4.21 ± 0.07	-4.19 ± 0.06	-4.20 ± 0.07
Archaea	*Aeropyrum pernix*	-5.01 ± 0.07	-5.13 ± 0.08	-5.31 ± 0.08	-5.36 ± 0.08	-5.35 ± 0.08	-5.42 ± 0.08	-5.40 ± 0.08	-5.37 ± 0.08	-5.47 ± 0.08	-5.38 ± 0.08	-5.40 ± 0.08	-5.36 ± 0.08	-5.38 ± 0.08
Archaea	*Caldivirga maquilingensis*	-3.16 ± 0.05	-3.21 ± 0.06	-3.44 ± 0.06	-3.47 ± 0.06	-3.42 ± 0.06	-3.54 ± 0.06	-3.51 ± 0.06	-3.33 ± 0.06	-3.40 ± 0.06	-3.41 ± 0.06	-3.46 ± 0.06	-3.46 ± 0.06	-3.50 ± 0.06
Archaea	*Desulfurococcus kamchatkensis*	-3.15 ± 0.06	-3.13 ± 0.06	-3.37 ± 0.07	-3.48 ± 0.07	-3.50 ± 0.07	-3.54 ± 0.07	-3.47 ± 0.07	-3.60 ± 0.07	-3.60 ± 0.07	-3.47 ± 0.07	-3.51 ± 0.07	-3.48 ± 0.07	-3.43 ± 0.07
Archaea	*Haloarcula marismortui*	-4.79 ± 0.05	-5.56 ± 0.05	-6.12 ± 0.06	-6.28 ± 0.06	-6.40 ± 0.06	-6.50 ± 0.06	-6.50 ± 0.06	-6.52 ± 0.06	-6.53 ± 0.06	-6.51 ± 0.06	-6.44 ± 0.05	-6.48 ± 0.06	-6.32 ± 0.05
Archaea	*Halobacterium salinarum*	-5.54 ± 0.06	-6.60 ± 0.07	-7.40 ± 0.08	-7.33 ± 0.07	-7.66 ± 0.08	-7.71 ± 0.08	-7.65 ± 0.08	-7.64 ± 0.08	-7.64 ± 0.08	-7.56 ± 0.08	-7.48 ± 0.08	-7.64 ± 0.08	-7.56 ± 0.08
Archaea	*Haloquadratum walsbyi*	-3.44 ± 0.05	-3.79 ± 0.05	-4.13 ± 0.06	-4.17 ± 0.05	-4.27 ± 0.05	-4.23 ± 0.05	-4.36 ± 0.06	-4.19 ± 0.05	-4.20 ± 0.05	-4.14 ± 0.05	-4.15 ± 0.05	-4.16 ± 0.05	-4.09 ± 0.05
Archaea	*Halorubrum lacusprofundi*	-5.10 ± 0.05	-6.24 ± 0.06	-6.92 ± 0.06	-7.05 ± 0.06	-7.21 ± 0.06	-7.53 ± 0.06	-7.40 ± 0.06	-7.39 ± 0.06	-7.41 ± 0.06	-7.28 ± 0.06	-7.29 ± 0.06	-7.37 ± 0.06	-7.25 ± 0.06
Archaea	*Hyperthermus butylicus*	-4.92 ± 0.07	-4.87 ± 0.07	-4.89 ± 0.07	-4.95 ± 0.07	-4.84 ± 0.07	-5.06 ± 0.07	-5.08 ± 0.07	-5.08 ± 0.07	-4.90 ± 0.07	-4.82 ± 0.07	-4.89 ± 0.07	-4.92 ± 0.07	-4.91 ± 0.07
Archaea	*Ignicoccus hospitalis*	-4.92 ± 0.08	-5.09 ± 0.09	-5.11 ± 0.09	-5.25 ± 0.09	-5.23 ± 0.09	-5.19 ± 0.09	-5.16 ± 0.09	-5.13 ± 0.08	-5.12 ± 0.09	-5.11 ± 0.09	-5.25 ± 0.09	-5.19 ± 0.08	-5.11 ± 0.09
Archaea	*Metallosphaera sedula*	-2.95 ± 0.05	-3.07 ± 0.05	-3.43 ± 0.05	-3.60 ± 0.06	-3.63 ± 0.06	-3.72 ± 0.06	-3.70 ± 0.06	-3.67 ± 0.06	-3.62 ± 0.05	-3.62 ± 0.05	-3.61 ± 0.06	-3.67 ± 0.06	-3.75 ± 0.05
Archaea	*Methanobrevibacter smithii*	-1.37 ± 0.04	-1.62 ± 0.04	-2.01 ± 0.05	-2.16 ± 0.05	-2.17 ± 0.05	-2.16 ± 0.05	-2.12 ± 0.05	-2.14 ± 0.05	-2.14 ± 0.05	-2.20 ± 0.05	-2.20 ± 0.05	-2.11 ± 0.05	-2.15 ± 0.05
Archaea	*Methanocaldococcus jannaschii*	-1.71 ± 0.04	-1.65 ± 0.04	-1.71 ± 0.05	-1.83 ± 0.05	-1.79 ± 0.05	-1.78 ± 0.05	-1.84 ± 0.05	-1.78 ± 0.05	-1.76 ± 0.05	-1.85 ± 0.05	-1.86 ± 0.05	-1.88 ± 0.05	-1.93 ± 0.05
Archaea	*Methanococcoides burtonii*	-1.92 ± 0.04	-2.26 ± 0.04	-2.98 ± 0.05	-3.24 ± 0.05	-3.34 ± 0.05	-3.40 ± 0.05	-3.34 ± 0.05	-3.33 ± 0.05	-3.28 ± 0.05	-3.30 ± 0.05	-3.19 ± 0.05	-3.23 ± 0.05	-3.23 ± 0.05
Archaea	*Methanococcus aeolicus*	-1.31 ± 0.04	-1.40 ± 0.04	-1.82 ± 0.05	-2.00 ± 0.06	-1.91 ± 0.05	-1.86 ± 0.05	-1.93 ± 0.05	-1.90 ± 0.05	-1.97 ± 0.06	-1.92 ± 0.05	-1.97 ± 0.05	-2.03 ± 0.06	-1.98 ± 0.05
Archaea	*Methanocorpusculum labreanum*	-2.58 ± 0.05	-3.29 ± 0.06	-4.24 ± 0.07	-4.49 ± 0.07	-4.25 ± 0.07	-4.44 ± 0.07	-4.42 ± 0.07	-4.41 ± 0.07	-4.37 ± 0.07	-4.43 ± 0.07	-4.37 ± 0.07	-4.46 ± 0.07	-4.49 ± 0.07
Archaea	*Methanoculleus marisnigri*	-4.18 ± 0.06	-4.98 ± 0.06	-5.82 ± 0.07	-6.19 ± 0.07	-6.30 ± 0.07	-6.47 ± 0.07	-6.46 ± 0.07	-6.61 ± 0.07	-6.48 ± 0.07	-6.32 ± 0.07	-6.45 ± 0.07	-6.50 ± 0.07	-6.33 ± 0.06
Archaea	*Methanopyrus kandleri*	-5.57 ± 0.07	-5.78 ± 0.07	-5.92 ± 0.07	-6.01 ± 0.08	-6.11 ± 0.08	-6.02 ± 0.07	-6.05 ± 0.07	-5.97 ± 0.07	-5.92 ± 0.07	-5.91 ± 0.07	-5.91 ± 0.07	-5.90 ± 0.07	-5.96 ± 0.07
Archaea	*Methanosaeta thermophila*	-3.88 ± 0.06	-4.36 ± 0.07	-4.82 ± 0.07	-5.04 ± 0.07	-5.25 ± 0.07	-5.32 ± 0.07	-5.29 ± 0.07	-5.20 ± 0.07	-5.09 ± 0.07	-5.06 ± 0.07	-5.12 ± 0.07	-5.18 ± 0.07	-5.15 ± 0.07
Archaea	*Methanosarcina acetivorans*	-2.31 ± 0.03	-2.52 ± 0.03	-2.95 ± 0.04	-3.13 ± 0.04	-3.25 ± 0.04	-3.40 ± 0.04	-3.50 ± 0.04	-3.45 ± 0.04	-3.48 ± 0.04	-3.36 ± 0.04	-3.37 ± 0.04	-3.44 ± 0.04	-3.43 ± 0.04
Archaea	*Methanosphaera stadtmanae*	-1.18 ± 0.04	-1.39 ± 0.04	-1.71 ± 0.05	-1.86 ± 0.05	-1.76 ± 0.05	-1.81 ± 0.05	-1.80 ± 0.05	-1.75 ± 0.05	-1.82 ± 0.05	-1.83 ± 0.05	-1.87 ± 0.05	-1.82 ± 0.05	-1.85 ± 0.05
Archaea	*Methanosphaerula palustris*	-3.31 ± 0.05	-4.02 ± 0.06	-4.90 ± 0.06	-5.10 ± 0.06	-5.13 ± 0.06	-5.20 ± 0.06	-5.35 ± 0.06	-5.28 ± 0.06	-5.28 ± 0.06	-5.25 ± 0.06	-5.35 ± 0.06	-5.46 ± 0.06	-5.36 ± 0.06
Archaea	*Methanospirillum hungatei*	-2.68 ± 0.04	-3.01 ± 0.04	-3.41 ± 0.05	-3.65 ± 0.05	-3.63 ± 0.05	-3.75 ± 0.05	-3.63 ± 0.05	-3.59 ± 0.05	-3.65 ± 0.05	-3.67 ± 0.05	-3.64 ± 0.05	-3.69 ± 0.05	-3.70 ± 0.05
Archaea	*Methanothermobacter thermautotrophicus*	-2.81 ± 0.05	-3.23 ± 0.06	-3.71 ± 0.06	-3.95 ± 0.07	-4.16 ± 0.07	-4.28 ± 0.07	-4.23 ± 0.07	-4.15 ± 0.07	-4.25 ± 0.07	-4.31 ± 0.07	-4.45 ± 0.07	-4.27 ± 0.07	-4.28 ± 0.07
Archaea	*Nanoarchaeum equitans*	-2.10 ± 0.09	-1.82 ± 0.08	-1.87 ± 0.09	-1.88 ± 0.10	-1.86 ± 0.09	-1.77 ± 0.09	-1.77 ± 0.09	-1.78 ± 0.08	-1.85 ± 0.08	-1.91 ± 0.09	-1.83 ± 0.08	-1.96 ± 0.09	-1.98 ± 0.09
Archaea	*Natronomonas pharaonis*	-5.15 ± 0.05	-6.17 ± 0.06	-6.85 ± 0.07	-7.06 ± 0.07	-7.08 ± 0.07	-7.10 ± 0.07	-7.07 ± 0.07	-7.05 ± 0.07	-6.96 ± 0.07	-6.82 ± 0.07	-6.90 ± 0.06	-6.89 ± 0.06	-6.77 ± 0.07
Archaea	*Nitrosopumilus maritimus*	-1.91 ± 0.04	-2.02 ± 0.05	-2.38 ± 0.05	-2.47 ± 0.05	-2.37 ± 0.05	-2.37 ± 0.05	-2.26 ± 0.05	-2.25 ± 0.05	-2.19 ± 0.05	-2.27 ± 0.05	-2.24 ± 0.05	-2.18 ± 0.05	-2.32 ± 0.05
Archaea	*Picrophilus torridus*	-2.03 ± 0.05	-2.26 ± 0.05	-2.60 ± 0.06	-2.66 ± 0.06	-2.64 ± 0.06	-2.76 ± 0.06	-2.64 ± 0.06	-2.61 ± 0.06	-2.68 ± 0.06	-2.65 ± 0.06	-2.67 ± 0.06	-2.62 ± 0.06	-2.67 ± 0.06
Archaea	*Pyrobaculum aerophilum*	-3.85 ± 0.05	-4.12 ± 0.06	-4.50 ± 0.06	-4.56 ± 0.06	-4.49 ± 0.06	-4.48 ± 0.06	-4.46 ± 0.06	-4.60 ± 0.06	-4.63 ± 0.06	-4.65 ± 0.06	-4.67 ± 0.06	-4.65 ± 0.06	-4.61 ± 0.06
Archaea	*Pyrococcus abyssi*	-2.99 ± 0.06	-3.05 ± 0.06	-3.24 ± 0.06	-3.30 ± 0.06	-3.23 ± 0.06	-3.29 ± 0.06	-3.38 ± 0.06	-3.40 ± 0.06	-3.38 ± 0.06	-3.45 ± 0.06	-3.30 ± 0.06	-3.37 ± 0.06	-3.36 ± 0.06
Archaea	*Staphylothermus marinus*	-2.19 ± 0.05	-2.09 ± 0.05	-2.21 ± 0.05	-2.37 ± 0.06	-2.33 ± 0.05	-2.30 ± 0.05	-2.32 ± 0.05	-2.26 ± 0.05	-2.36 ± 0.05	-2.24 ± 0.05	-2.32 ± 0.05	-2.47 ± 0.05	-2.38 ± 0.05
Archaea	*Sulfolobus acidocaldarius*	-2.09 ± 0.04	-2.15 ± 0.04	-2.17 ± 0.04	-2.33 ± 0.05	-2.27 ± 0.05	-2.41 ± 0.05	-2.31 ± 0.04	-2.25 ± 0.04	-2.36 ± 0.05	-2.44 ± 0.05	-2.35 ± 0.05	-2.42 ± 0.05	-2.39 ± 0.05
Archaea	*Thermococcus gammatolerans*	-3.95 ± 0.05	-4.13 ± 0.06	-4.38 ± 0.06	-4.55 ± 0.06	-4.76 ± 0.06	-4.91 ± 0.06	-4.80 ± 0.06	-4.73 ± 0.06	-4.74 ± 0.06	-4.69 ± 0.06	-4.74 ± 0.06	-4.70 ± 0.06	-4.55 ± 0.06
Archaea	*Thermofilum pendens*	-4.74 ± 0.07	-4.94 ± 0.07	-5.33 ± 0.07	-5.36 ± 0.07	-5.46 ± 0.07	-5.39 ± 0.07	-5.37 ± 0.07	-5.52 ± 0.07	-5.49 ± 0.07	-5.47 ± 0.07	-5.62 ± 0.07	-5.62 ± 0.07	-5.46 ± 0.07
Archaea	*Thermoplasma acidophilum*	-2.95 ± 0.06	-3.21 ± 0.06	-3.62 ± 0.07	-3.61 ± 0.07	-3.75 ± 0.07	-3.69 ± 0.07	-3.63 ± 0.07	-3.62 ± 0.07	-3.78 ± 0.07	-3.77 ± 0.07	-3.80 ± 0.07	-3.71 ± 0.07	-3.76 ± 0.07
Archaea	*Thermoproteus neutrophilus*	-5.11 ± 0.07	-5.51 ± 0.07	-5.95 ± 0.08	-5.89 ± 0.08	-5.92 ± 0.08	-5.88 ± 0.07	-5.93 ± 0.07	-6.07 ± 0.08	-6.13 ± 0.08	-5.94 ± 0.08	-6.02 ± 0.07	-6.01 ± 0.07	-6.00 ± 0.08

Continued on next page

Type	Species	Window 1	Window 2	Window 3	Window 4	Window 5	Window 6	Window 7	Window 8	Window 9	Window 10	Window 11	Window 12	Window 13
Fungi	*Aspergillus fumigatus*	-3.77 ± 0.03	-4.11 ± 0.03	-4.69 ± 0.03	-4.55 ± 0.03	-4.22 ± 0.03	-4.04 ± 0.03	-3.92 ± 0.03	-3.85 ± 0.03	-3.97 ± 0.03	-3.99 ± 0.03	-3.94 ± 0.03	-3.97 ± 0.03	-3.98 ± 0.03
Fungi	*Candida dubliniensis*	-1.65 ± 0.02	-1.77 ± 0.02	-2.29 ± 0.03	-2.30 ± 0.03	-2.16 ± 0.03	-2.10 ± 0.03	-2.05 ± 0.03	-2.01 ± 0.03	-1.97 ± 0.03	-1.96 ± 0.03	-1.92 ± 0.03	-1.97 ± 0.03	-1.96 ± 0.03
Fungi	*Cryptococcus neoformans*	-3.01 ± 0.03	-3.11 ± 0.03	-3.57 ± 0.04	-3.67 ± 0.04	-3.53 ± 0.04	-3.42 ± 0.04	-3.43 ± 0.03	-3.41 ± 0.03	-3.42 ± 0.03	-3.46 ± 0.03	-3.46 ± 0.03	-3.50 ± 0.03	-3.51 ± 0.03
Fungi	*Debaryomyces hansenii*	-2.12 ± 0.03	-2.32 ± 0.03	-2.86 ± 0.03	-2.90 ± 0.03	-2.79 ± 0.03	-2.62 ± 0.03	-2.55 ± 0.03	-2.47 ± 0.03	-2.44 ± 0.03	-2.38 ± 0.03	-2.36 ± 0.03	-2.39 ± 0.03	-2.38 ± 0.03
Fungi	*Encephalitozoon cuniculi*	-3.02 ± 0.05	-3.21 ± 0.06	-3.44 ± 0.06	-3.53 ± 0.05	-3.57 ± 0.06	-3.80 ± 0.06	-3.76 ± 0.06	-3.69 ± 0.06	-3.74 ± 0.06	-3.61 ± 0.05	-3.69 ± 0.06	-3.61 ± 0.06	-3.59 ± 0.06
Fungi	*Eremothecium gossypii*	-3.69 ± 0.04	-4.33 ± 0.04	-4.98 ± 0.05	-5.35 ± 0.05	-5.29 ± 0.05	-5.28 ± 0.05	-5.13 ± 0.05	-5.17 ± 0.05	-5.18 ± 0.05	-5.12 ± 0.05	-5.11 ± 0.05	-5.23 ± 0.05	-5.18 ± 0.05
Fungi	*Kluyveromyces lactis*	-2.49 ± 0.03	-2.90 ± 0.03	-3.39 ± 0.03	-3.47 ± 0.04	-3.22 ± 0.03	-3.04 ± 0.03	-2.92 ± 0.03	-2.83 ± 0.03	-2.76 ± 0.03	-2.80 ± 0.03	-2.75 ± 0.03	-2.79 ± 0.03	-2.85 ± 0.03
Fungi	*Pichia stipitis*	-2.73 ± 0.03	-3.18 ± 0.03	-3.89 ± 0.04	-3.91 ± 0.04	-3.69 ± 0.04	-3.52 ± 0.04	-3.34 ± 0.04	-3.14 ± 0.03	-3.10 ± 0.03	-3.03 ± 0.03	-2.91 ± 0.03	-2.94 ± 0.03	-2.98 ± 0.03
Fungi	*Saccharomyces cerevisiae*	-2.31 ± 0.02	-2.43 ± 0.03	-2.90 ± 0.03	-3.01 ± 0.03	-2.96 ± 0.03	-2.85 ± 0.03	-2.75 ± 0.03	-2.71 ± 0.03	-2.69 ± 0.03	-2.67 ± 0.03	-2.66 ± 0.03	-2.67 ± 0.03	-2.72 ± 0.03
Fungi	*Schizosaccharomyces pombe*	-2.53 ± 0.03	-2.51 ± 0.03	-2.89 ± 0.03	-3.01 ± 0.04	-2.87 ± 0.03	-2.84 ± 0.03	-2.79 ± 0.03	-2.67 ± 0.03	-2.73 ± 0.03	-2.70 ± 0.03	-2.68 ± 0.03	-2.69 ± 0.03	-2.64 ± 0.03
Fungi	*Yarrowia lipolytica*	-3.03 ± 0.03	-3.75 ± 0.03	-4.78 ± 0.04	-5.02 ± 0.04	-4.75 ± 0.04	-4.63 ± 0.04	-4.46 ± 0.04	-4.32 ± 0.04	-4.26 ± 0.04	-4.25 ± 0.04	-4.23 ± 0.04	-4.26 ± 0.03	-4.25 ± 0.03
Plant	*Arabidopsis thaliana*	-3.12 ± 0.01	-2.90 ± 0.01	-3.04 ± 0.02	-3.07 ± 0.02	-3.06 ± 0.02	-3.06 ± 0.02	-3.04 ± 0.01	-3.01 ± 0.01	-3.06 ± 0.01	-3.07 ± 0.01	-3.07 ± 0.01	-3.10 ± 0.01	-3.10 ± 0.01
Plant	*Oryza sativa*	-5.98 ± 0.02	-6.05 ± 0.03	-6.32 ± 0.03	-6.38 ± 0.03	-6.42 ± 0.03	-6.40 ± 0.03	-6.28 ± 0.03	-6.21 ± 0.03	-6.15 ± 0.03	-6.08 ± 0.03	-6.06 ± 0.03	-6.03 ± 0.03	-6.00 ± 0.03
Insect	*Anopheles gambiae*	-3.89 ± 0.03	-4.34 ± 0.03	-5.00 ± 0.03	-5.17 ± 0.03	-5.01 ± 0.03	-4.88 ± 0.03	-4.75 ± 0.03	-4.72 ± 0.03	-4.80 ± 0.03	-4.70 ± 0.03	-4.69 ± 0.03	-4.78 ± 0.03	-4.78 ± 0.03
Insect	*Drosophila melanogaster*	-3.76 ± 0.02	-4.17 ± 0.02	-4.88 ± 0.03	-5.02 ± 0.03	-4.84 ± 0.03	-4.70 ± 0.03	-4.63 ± 0.03	-4.61 ± 0.03	-4.59 ± 0.02	-4.55 ± 0.03	-4.56 ± 0.02	-4.58 ± 0.02	-4.56 ± 0.02
Fish	*Danio rerio*	-3.86 ± 0.02	-3.89 ± 0.02	-4.28 ± 0.02	-4.30 ± 0.02	-4.13 ± 0.02	-4.08 ± 0.02	-3.99 ± 0.02	-4.00 ± 0.02	-3.95 ± 0.02	-3.98 ± 0.02	-3.98 ± 0.02	-3.98 ± 0.02	-3.95 ± 0.02
Fish	*Gasterosteus aculeatus*	-4.63 ± 0.03	-4.87 ± 0.03	-5.16 ± 0.03	-5.22 ± 0.03	-5.08 ± 0.03	-5.08 ± 0.03	-4.99 ± 0.03	-4.99 ± 0.03	-4.98 ± 0.03	-4.91 ± 0.03	-5.00 ± 0.03	-5.03 ± 0.03	-5.01 ± 0.03
Fish	*Oryzias latipes*	-4.30 ± 0.03	-4.42 ± 0.03	-4.75 ± 0.03	-4.77 ± 0.03	-4.65 ± 0.03	-4.61 ± 0.03	-4.51 ± 0.03	-4.55 ± 0.03	-4.56 ± 0.03	-4.50 ± 0.03	-4.56 ± 0.03	-4.58 ± 0.03	-4.55 ± 0.03
Fish	*Takifugu rubripes*	-4.28 ± 0.03	-4.43 ± 0.03	-4.81 ± 0.03	-4.84 ± 0.03	-4.70 ± 0.03	-4.66 ± 0.03	-4.58 ± 0.03	-4.59 ± 0.03	-4.57 ± 0.03	-4.54 ± 0.03	-4.54 ± 0.03	-4.63 ± 0.03	-4.62 ± 0.03
Bird	*Gallus gallus*	-5.25 ± 0.04	-5.38 ± 0.04	-5.53 ± 0.04	-5.50 ± 0.04	-5.33 ± 0.04	-5.32 ± 0.04	-5.13 ± 0.04	-4.89 ± 0.04	-4.92 ± 0.04	-4.79 ± 0.04	-4.84 ± 0.04	-4.76 ± 0.04	-4.63 ± 0.03
Bird	*Taeniopygia guttata*	-5.05 ± 0.05	-5.17 ± 0.05	-5.37 ± 0.05	-5.37 ± 0.05	-5.24 ± 0.05	-5.13 ± 0.05	-5.04 ± 0.05	-4.86 ± 0.05	-4.78 ± 0.05	-4.69 ± 0.04	-4.68 ± 0.05	-4.76 ± 0.05	-4.67 ± 0.05
Mammal	*Bos taurus*	-5.33 ± 0.03	-5.45 ± 0.03	-5.76 ± 0.03	-5.76 ± 0.03	-5.62 ± 0.03	-5.47 ± 0.03	-5.27 ± 0.03	-5.10 ± 0.03	-5.12 ± 0.03	-4.96 ± 0.03	-5.03 ± 0.03	-5.01 ± 0.03	-4.92 ± 0.03
Mammal	*Canis familiaris*	-5.38 ± 0.03	-5.50 ± 0.04	-5.78 ± 0.04	-5.75 ± 0.04	-5.67 ± 0.04	-5.44 ± 0.04	-5.27 ± 0.03	-5.13 ± 0.03	-5.12 ± 0.03	-4.96 ± 0.03	-4.96 ± 0.03	-4.94 ± 0.03	-4.87 ± 0.03
Mammal	*Equus caballus*	-4.99 ± 0.03	-5.02 ± 0.03	-5.32 ± 0.03	-5.27 ± 0.03	-5.21 ± 0.03	-5.04 ± 0.03	-4.88 ± 0.03	-4.69 ± 0.03	-4.66 ± 0.03	-4.60 ± 0.03	-4.66 ± 0.03	-4.71 ± 0.03	-4.61 ± 0.03
Mammal	*Homo sapiens*	-5.38 ± 0.02	-5.51 ± 0.03	-5.67 ± 0.03	-5.61 ± 0.03	-5.54 ± 0.03	-5.48 ± 0.02	-5.29 ± 0.02	-5.17 ± 0.02	-5.12 ± 0.02	-5.01 ± 0.02	-5.03 ± 0.02	-5.00 ± 0.02	-4.96 ± 0.02
Mammal	*Loxodonta africana*	-4.93 ± 0.06	-5.03 ± 0.06	-5.51 ± 0.06	-5.53 ± 0.06	-5.41 ± 0.06	-5.22 ± 0.06	-5.06 ± 0.06	-4.83 ± 0.06	-4.73 ± 0.06	-4.68 ± 0.06	-4.80 ± 0.06	-4.89 ± 0.06	-4.69 ± 0.06
Mammal	*Mus musculus*	-5.04 ± 0.02	-5.09 ± 0.02	-5.35 ± 0.02	-5.30 ± 0.02	-5.22 ± 0.02	-5.10 ± 0.02	-4.93 ± 0.02	-4.76 ± 0.02	-4.74 ± 0.02	-4.64 ± 0.02	-4.67 ± 0.02	-4.68 ± 0.02	-4.62 ± 0.02
Mammal	*Pteropus vampyrus*	-5.49 ± 0.04	-5.63 ± 0.04	-5.86 ± 0.04	-5.81 ± 0.04	-5.66 ± 0.04	-5.57 ± 0.04	-5.41 ± 0.04	-5.29 ± 0.04	-5.21 ± 0.04	-5.09 ± 0.04	-5.05 ± 0.04	-5.05 ± 0.04	-4.99 ± 0.04
Mammal	*Tursiops truncatus*	-5.52 ± 0.04	-5.68 ± 0.04	-5.87 ± 0.04	-5.86 ± 0.04	-5.78 ± 0.04	-5.65 ± 0.04	-5.41 ± 0.04	-5.33 ± 0.04	-5.29 ± 0.04	-5.14 ± 0.04	-5.11 ± 0.04	-5.10 ± 0.04	-5.05 ± 0.04
