# Supplementary material for: A Universal Trend of Reduced mRNA Stability near the Translation-Initiation Site in Prokaryotes and Eukaryotes
Source: PLoS Comput Biol. 2010 Feb 5;6(2):e1000664. doi: 10.1371/journal.pcbi.1000664 (PMC2816680; doi:10.1371/journal.pcbi.1000664)
Supplement: Table S3 — Intra-genome comparison on mean ZΔG of the first window. (0.06 MB PDF) [file pcbi.1000664.s003.pdf]

Table S3: Intra-genome comparison on mean $Z_{\Delta G}$ of the first window

		GC			ENC		
Type	Species	Highest 5%	Lowest 5%	P-value	Highest 5%	Lowest 5%	P-value
Bacteria	*Acaryochloris marina*	0.37	0.03	1.0×10^{-5}	0.21	0.09	1.5×10^{-1}
Bacteria	*Acholeplasma laidlawii*	-0.02	0.09	4.6×10^{-1}	0.02	0.29	9.6×10^{-2}
Bacteria	*Acidiphilium cryptum*	0.48	0.09	2.8×10^{-3}	0.02	0.45	1.0×10^{-3}
Bacteria	*Acidithiobacillus ferrooxidans*	0.29	0.11	1.0×10^{-1}	0.30	0.57	1.5×10^{-2}
Bacteria	*Acidobacteria bacterium*	0.44	0.27	6.7×10^{-2}	0.29	0.42	1.5×10^{-1}
Bacteria	*Acidobacterium capsulatum*	0.73	0.14	4.1×10^{-7}	0.18	0.40	7.3×10^{-2}
Bacteria	*Acidothermus cellulolyticus*	0.26	0.12	3.1×10^{-1}	0.22	0.15	6.4×10^{-1}
Bacteria	*Acidovorax citrulli*	0.69	0.21	2.2×10^{-7}	0.22	1.00	2.7×10^{-14}
Bacteria	*Acinetobacter baumannii*	0.22	0.02	3.6×10^{-2}	0.07	0.07	9.6×10^{-1}
Bacteria	*Actinobacillus pleuropneumoniae*	0.33	-0.06	3.9×10^{-3}	0.14	0.17	8.3×10^{-1}
Bacteria	*Aeromonas hydrophila*	0.68	0.36	2.2×10^{-3}	0.31	0.66	1.1×10^{-3}
Bacteria	*Agrobacterium radiobacter*	0.72	0.16	3.1×10^{-11}	0.28	0.67	4.1×10^{-6}
Bacteria	*Akkermansia muciniphila*	0.57	0.18	2.4×10^{-3}	0.25	0.74	9.9×10^{-5}
Bacteria	*Alcanivorax borkumensis*	0.44	0.22	5.6×10^{-2}	0.37	0.44	5.2×10^{-1}
Bacteria	*Aliivibrio salmonicida*	0.25	-0.13	1.3×10^{-4}	0.67	0.23	9.1×10^{-5}
Bacteria	*Alkalilimnicola ehrlichii*	0.54	0.30	4.1×10^{-2}	0.29	0.58	1.8×10^{-2}
Bacteria	*Alkaliphilus metalliredigens*	0.18	-0.06	9.5×10^{-3}	0.22	0.21	9.6×10^{-1}
Bacteria	*Alteromonas macleodii*	0.20	0.13	4.3×10^{-1}	0.21	0.25	7.3×10^{-1}
Bacteria	*Anabaena variabilis*	0.17	-0.01	3.5×10^{-2}	0.07	0.14	4.6×10^{-1}
Bacteria	*Anaerocellum thermophilum*	0.08	-0.05	2.4×10^{-1}	0.09	0.18	4.5×10^{-1}
Bacteria	*Anaeromyxobacter dehalogenans*	0.69	0.49	4.5×10^{-2}	0.39	0.74	2.8×10^{-4}
Bacteria	*Anaplasma marginale*	0.09	-0.13	3.3×10^{-1}	0.09	0.16	7.3×10^{-1}
Bacteria	*Anoxybacillus flavithermus*	0.09	-0.14	6.3×10^{-2}	-0.07	0.00	5.5×10^{-1}
Bacteria	*Aquifex aeolicus*	0.29	-0.28	8.4×10^{-4}	0.03	0.09	6.8×10^{-1}
Bacteria	*Archaeoglobus fulgidus*	0.26	-0.04	2.0×10^{-2}	-0.03	-0.02	9.7×10^{-1}
Bacteria	*Arcobacter butzleri*	0.04	0.08	7.5×10^{-1}	-0.03	0.26	1.8×10^{-2}
Bacteria	*Aromatoleum aromaticum*	0.63	0.12	1.8×10^{-6}	-0.10	0.70	9.9×10^{-14}
Bacteria	*Arthrobacter aurescens*	0.30	0.09	3.8×10^{-2}	0.26	0.28	8.5×10^{-1}
Bacteria	*Aster yellows*	-0.03	-0.26	3.2×10^{-1}	-0.07	0.16	3.0×10^{-1}
Bacteria	*Azoarcus sp. BH72*	0.72	0.32	1.8×10^{-4}	0.21	0.91	9.7×10^{-11}
Bacteria	*Azorhizobium caulinodans*	0.49	0.30	4.7×10^{-2}	0.22	0.54	1.8×10^{-3}
Bacteria	*Azotobacter vinelandii*	0.67	0.08	2.4×10^{-9}	-0.13	0.69	1.0×10^{-16}
Bacteria	*Bacillus amyloliquefaciens*	0.29	0.12	5.6×10^{-2}	0.24	0.31	5.2×10^{-1}
Bacteria	*Bacteroides fragilis*	0.69	0.08	5.5×10^{-11}	0.39	0.24	1.2×10^{-1}
Bacteria	*Bartonella bacilliformis*	0.06	-0.08	4.6×10^{-1}	0.16	0.06	5.6×10^{-1}
Bacteria	*Baumannia cicadellinicola*	0.24	-0.18	8.6×10^{-2}	0.06	0.02	8.4×10^{-1}
Bacteria	*Bdellovibrio bacteriovorus*	0.46	0.24	3.9×10^{-2}	0.30	0.30	1
Bacteria	*Beijerinckia indica*	0.47	0.11	8.9×10^{-4}	0.18	0.55	2.6×10^{-4}
Bacteria	*Beutenbergia cavernae*	0.33	0.38	6.2×10^{-1}	0.30	0.61	3.6×10^{-3}
Bacteria	*Bifidobacterium adolescentis*	0.35	0.23	4.8×10^{-1}	0.16	0.52	4.5×10^{-2}
Bacteria	*Bordetella avium*	0.62	0.39	3.8×10^{-2}	0.35	0.65	7.6×10^{-3}
Bacteria	*Borrelia afzelii*	0.10	-0.02	6.3×10^{-1}	-0.31	-0.05	2.6×10^{-1}
Bacteria	*Brachyspira hyodysenteriae*	-0.04	0.07	3.9×10^{-1}	0.23	0.17	5.6×10^{-1}
Bacteria	*Bradyrhizobium japonicum*	0.62	0.01	4.9×10^{-16}	0.04	0.72	1.7×10^{-19}
Bacteria	*Brevibacillus brevis*	0.24	0.07	3.7×10^{-2}	0.12	0.09	6.9×10^{-1}
Bacteria	*Brucella abortus*	0.57	0.03	2.7×10^{-6}	-0.06	0.57	8.3×10^{-7}
Bacteria	*Buchnera aphidicola*	0.07	-0.05	5.9×10^{-1}	0.11	-0.16	2.9×10^{-1}
Bacteria	*Burkholderia ambifaria*	0.83	0.36	1.5×10^{-8}	0.18	1.12	3.2×10^{-25}
Bacteria	*Caldicellulosiruptor saccharolyticus*	0.15	0.03	3.1×10^{-1}	0.12	-0.06	1.7×10^{-1}
Bacteria	*Campylobacter concisus*	0.42	0.10	1.7×10^{-2}	0.12	0.14	9.2×10^{-1}
Bacteria	*Candidatus Amoebophilus*	0.12	0.25	4.1×10^{-1}	0.07	0.08	9.5×10^{-1}
Bacteria	*Carboxydothermus hydrogenoformans*	0.21	-0.05	6.6×10^{-2}	0.04	-0.03	5.7×10^{-1}
Bacteria	*Caulobacter crescentus*	0.64	0.12	6.2×10^{-6}	-0.13	0.78	5.3×10^{-14}
Bacteria	*Cellvibrio japonicus*	0.62	0.01	2.4×10^{-8}	0.04	0.54	4.7×10^{-6}
Bacteria	*Chlamydia muridarum*	0.07	-0.02	6.8×10^{-1}	0.16	-0.02	3.9×10^{-1}
Bacteria	*Chlamydophila abortus*	0.10	0.04	7.6×10^{-1}	-0.13	0.04	3.9×10^{-1}
Bacteria	*Chlorobaculum parvum*	0.51	0.15	7.8×10^{-3}	0.30	0.69	2.4×10^{-3}
Bacteria	*Chlorobium chlorochromatii*	0.23	0.06	2.7×10^{-1}	0.32	0.26	5.9×10^{-1}
Bacteria	*Chloroflexus aggregans*	0.24	0.17	4.9×10^{-1}	0.24	0.25	9.0×10^{-1}
Bacteria	*Chloroherpeton thalassium*	0.52	0.11	3.1×10^{-4}	0.25	0.26	9.1×10^{-1}
Bacteria	*Chromobacterium violaceum*	0.53	-0.03	2.1×10^{-7}	0.04	0.85	9.9×10^{-13}
Bacteria	*Chromohalobacter salexigens*	0.51	0.15	5.4×10^{-4}	0.19	0.62	2.3×10^{-5}
Bacteria	*Citrobacter koseri*	0.41	-0.16	1.1×10^{-8}	0.16	0.38	3.0×10^{-2}
Bacteria	*Clavibacter michiganensis*	0.65	0.36	1.6×10^{-2}	0.44	0.63	1.0×10^{-1}
Bacteria	*Clostridium acetobutylicum*	0.12	-0.04	1.2×10^{-1}	0.12	0.17	5.8×10^{-1}
Bacteria	*Colwellia psychrerythraea*	0.22	0.12	2.7×10^{-1}	0.10	0.09	9.4×10^{-1}
Bacteria	*Coprothermobacter proteolyticus*	0.09	-0.06	3.8×10^{-1}	0.19	-0.01	2.5×10^{-1}
Bacteria	*Corynebacterium aurimucosum*	0.55	-0.07	1.5×10^{-6}	0.05	0.62	6.9×10^{-5}
Bacteria	*Coxiella burnetii*	0.01	-0.02	8.1×10^{-1}	0.03	0.08	7.3×10^{-1}
Bacteria	*Cronobacter sakazakii*	0.60	0.01	6.6×10^{-9}	0.27	0.44	9.4×10^{-2}
Bacteria	*Cupriavidus taiwanensis*	0.80	0.28	1.1×10^{-8}	0.13	1.02	3.2×10^{-18}
Bacteria	*Cyanothece sp. PCC 7424*	0.23	0.07	7.1×10^{-2}	0.31	0.07	5.1×10^{-3}

Continued on next page

		GC			ENC		
Type	Species	Highest 5%	Lowest 5%	P-value	Highest 5%	Lowest 5%	P-value
Bacteria	*Cytophaga hutchinsonii*	0.34	-0.06	1.5×10^{-4}	0.19	0.12	4.5×10^{-1}
Bacteria	*Dechloromonas aromatica*	0.63	0.22	3.1×10^{-5}	0.21	0.79	9.0×10^{-10}
Bacteria	*Dehalococcoides ethenogenes*	0.32	0.05	1.1×10^{-1}	0.19	0.27	6.5×10^{-1}
Bacteria	*Deinococcus deserti*	0.45	0.31	2.4×10^{-1}	0.23	0.83	7.0×10^{-6}
Bacteria	*Delftia acidovorans*	0.78	0.17	6.5×10^{-12}	0.11	0.98	3.3×10^{-21}
Bacteria	*Desulfatibacillum alkenivorans*	0.66	0.24	9.1×10^{-6}	0.41	0.75	4.1×10^{-5}
Bacteria	*Desulfitobacterium hafniense*	0.52	0.12	2.9×10^{-6}	0.17	0.22	5.5×10^{-1}
Bacteria	*Desulfobacterium autotrophicum*	0.40	0.25	6.9×10^{-2}	0.24	0.16	3.1×10^{-1}
Bacteria	*Desulfococcus oleovorans*	0.60	0.06	1.3×10^{-6}	0.09	0.58	4.8×10^{-5}
Bacteria	*Desulfotalea psychrophila*	0.38	0.03	1.8×10^{-3}	0.16	0.48	5.0×10^{-3}
Bacteria	*Desulfotomaculum reducens*	0.10	0.06	6.8×10^{-1}	0.08	0.00	4.4×10^{-1}
Bacteria	*Desulfovibrio desulfuricans*	0.33	0.10	5.8×10^{-2}	0.05	0.46	1.8×10^{-3}
Bacteria	*Diaphorobacter sp. TPSY*	0.78	0.52	1.2×10^{-2}	0.42	1.14	9.1×10^{-10}
Bacteria	*Dichelobacter nodosus*	0.24	0.08	4.3×10^{-1}	0.12	0.08	8.4×10^{-1}
Bacteria	*Dickeya dadantii*	0.60	0.19	2.9×10^{-5}	0.19	0.53	1.7×10^{-3}
Bacteria	*Dictyoglomus thermophilum*	0.21	-0.24	1.9×10^{-3}	0.06	-0.09	3.0×10^{-1}
Bacteria	*Dinoroseobacter shibae*	0.65	0.43	4.4×10^{-2}	0.44	0.77	2.3×10^{-3}
Bacteria	*Edwardsiella ictaluri*	0.58	-0.08	5.1×10^{-9}	0.07	0.53	3.9×10^{-5}
Bacteria	*Ehrlichia canis*	-0.30	0.03	1.8×10^{-1}	0.13	-0.18	1.3×10^{-1}
Bacteria	*Elusimicrobium minutum*	0.24	0.13	5.3×10^{-1}	0.37	0.28	5.4×10^{-1}
Bacteria	*Enterobacter sp. 638*	0.53	0.11	9.9×10^{-6}	0.14	0.46	8.6×10^{-4}
Bacteria	*Enterococcus faecalis*	0.13	-0.05	1.3×10^{-1}	0.02	0.01	9.7×10^{-1}
Bacteria	*Erwinia tasmaniensis*	0.44	0.05	4.6×10^{-4}	0.18	0.55	5.7×10^{-4}
Bacteria	*Erythrobacter litoralis*	0.58	0.13	5.9×10^{-5}	0.23	0.63	3.5×10^{-4}
Bacteria	*Escherichia coli*	0.51	0.07	9.3×10^{-6}	0.18	0.39	2.9×10^{-2}
Bacteria	*Eubacterium eligens*	0.26	-0.06	4.7×10^{-2}	0.24	0.38	3.8×10^{-1}
Bacteria	*Exiguobacterium sibiricum*	0.53	0.14	2.2×10^{-4}	0.32	0.24	5.1×10^{-1}
Bacteria	*Fervidobacterium nodosum*	0.03	0.00	8.5×10^{-1}	0.17	0.02	3.0×10^{-1}
Bacteria	*Finegoldia magna*	0.36	-0.08	1.5×10^{-3}	0.14	-0.32	2.8×10^{-1}
Bacteria	*Flavobacterium johnsoniae*	0.38	0.23	4.1×10^{-2}	0.26	0.23	7.1×10^{-1}
Bacteria	*Francisella novicida*	0.29	0.01	4.6×10^{-2}	0.25	0.13	3.9×10^{-1}
Bacteria	*Frankia alni*	0.22	0.06	5.8×10^{-2}	-0.14	0.18	2.2×10^{-4}
Bacteria	*Fusobacterium nucleatum*	0.23	-0.04	8.2×10^{-2}	-0.25	-0.10	3.9×10^{-1}
Bacteria	*Gemmatimonas aurantiaca*	0.46	0.27	6.6×10^{-2}	0.07	0.52	1.1×10^{-5}
Bacteria	*Geobacillus kaustophilus*	0.28	0.06	3.0×10^{-2}	0.06	0.31	2.3×10^{-2}
Bacteria	*Geobacter bemidjiensis*	0.54	0.24	4.2×10^{-3}	0.32	0.83	4.0×10^{-8}
Bacteria	*Gloeobacter violaceus*	0.49	0.14	4.2×10^{-4}	0.27	0.62	7.1×10^{-5}
Bacteria	*Gluconacetobacter diazotrophicus*	0.63	0.30	1.6×10^{-3}	0.37	0.66	7.0×10^{-3}
Bacteria	*Gluconobacter oxydans*	0.54	0.02	8.7×10^{-5}	-0.05	0.51	7.2×10^{-5}
Bacteria	*Gramella forsetii*	0.36	0.09	2.7×10^{-3}	0.36	0.20	7.7×10^{-2}
Bacteria	*Granulibacter bethesdensis*	0.33	0.04	3.9×10^{-2}	0.07	0.20	3.7×10^{-1}
Bacteria	*Haemophilus ducreyi*	0.17	-0.16	1.9×10^{-1}	0.04	0.24	2.3×10^{-1}
Bacteria	*Hahella chejuensis*	0.54	0.05	1.8×10^{-9}	0.17	0.44	4.0×10^{-4}
Bacteria	*Halorhodospira halophila*	0.43	0.32	4.1×10^{-1}	0.23	0.60	4.7×10^{-3}
Bacteria	*Halothermothrix orenii*	0.15	-0.08	8.7×10^{-2}	0.20	0.04	2.0×10^{-1}
Bacteria	*Helicobacter acinonychis*	0.07	0.16	5.2×10^{-1}	-0.08	0.10	2.0×10^{-1}
Bacteria	*Heliobacterium modesticaldum*	0.43	-0.11	1.1×10^{-4}	0.12	0.18	6.5×10^{-1}
Bacteria	*Herminiimonas arsenicoxydans*	0.58	0.13	3.4×10^{-5}	0.24	0.59	1.4×10^{-3}
Bacteria	*Herpetosiphon aurantiacus*	0.39	0.06	3.9×10^{-5}	0.24	0.22	8.3×10^{-1}
Bacteria	*Hydrogenobaculum sp. Y04AAS1*	-0.11	-0.08	8.7×10^{-1}	-0.25	-0.07	3.2×10^{-1}
Bacteria	*Hyphomonas neptunium*	0.44	0.26	1.2×10^{-1}	0.21	0.58	1.3×10^{-3}
Bacteria	*Idiomarina loihiensis*	0.24	0.09	2.0×10^{-1}	0.05	0.15	4.3×10^{-1}
Bacteria	*Jannaschia sp. CCS1*	0.68	0.29	3.2×10^{-5}	0.36	0.69	8.6×10^{-4}
Bacteria	*Janthinobacterium sp. Marseille*	0.54	0.23	2.3×10^{-3}	0.18	0.70	1.7×10^{-6}
Bacteria	*Kineococcus radiotolerans*	0.40	0.21	7.2×10^{-2}	0.08	0.27	6.3×10^{-2}
Bacteria	*Klebsiella pneumoniae*	0.72	0.12	2.3×10^{-11}	0.26	0.77	2.7×10^{-8}
Bacteria	*Kocuria rhizophila*	0.34	0.33	9.5×10^{-1}	0.35	0.51	2.7×10^{-1}
Bacteria	*Kosmotoga olearia*	0.17	-0.06	9.1×10^{-2}	0.11	-0.10	1.4×10^{-1}
Bacteria	*Lactobacillus acidophilus*	0.14	0.05	4.8×10^{-1}	0.12	0.08	8.2×10^{-1}
Bacteria	*Lactococcus lactis*	0.03	0.22	1.4×10^{-1}	0.23	0.10	3.2×10^{-1}
Bacteria	*Laribacter hongkongensis*	0.67	0.03	3.6×10^{-8}	0.27	0.63	8.0×10^{-4}
Bacteria	*Lawsonia intracellularis*	0.10	-0.29	5.9×10^{-2}	-0.12	0.06	3.6×10^{-1}
Bacteria	*Legionella pneumophila*	0.21	-0.04	2.4×10^{-2}	0.13	0.08	6.3×10^{-1}
Bacteria	*Leifsonia xyli*	0.37	0.09	4.8×10^{-2}	0.25	0.39	4.1×10^{-1}
Bacteria	*Leptospira biflexa*	0.39	0.06	4.7×10^{-4}	0.07	0.04	7.2×10^{-1}
Bacteria	*Leptothrix cholodnii*	0.82	0.48	6.5×10^{-4}	0.49	0.89	1.2×10^{-4}
Bacteria	*Leuconostoc citreum*	0.11	0.16	7.2×10^{-1}	0.29	-0.05	2.8×10^{-2}
Bacteria	*Listeria innocua*	0.15	-0.08	1.1×10^{-1}	0.15	0.06	5.3×10^{-1}
Bacteria	*Lysinibacillus sphaericus*	0.03	0.06	7.0×10^{-1}	0.14	-0.04	9.2×10^{-2}
Bacteria	*Macrococcus caseolyticus*	0.43	0.21	8.6×10^{-2}	0.26	0.18	4.6×10^{-1}
Bacteria	*Magnetococcus sp. MC-1*	0.34	-0.09	1.0×10^{-4}	0.10	0.26	1.3×10^{-1}
Bacteria	*Magnetospirillum magneticum*	0.39	0.29	2.9×10^{-1}	0.18	0.61	7.7×10^{-5}
Bacteria	*Mannheimia succiniciproducens*	0.37	0.04	1.2×10^{-2}	0.24	0.01	1.6×10^{-1}

Continued on next page

		GC			ENC		
Type	Species	Highest 5%	Lowest 5%	P-value	Highest 5%	Lowest 5%	P-value
Bacteria	*Maricaulis maris*	0.46	0.42	7.3×10^{-1}	0.36	0.47	3.0×10^{-1}
Bacteria	*Marinobacter aquaeolei*	0.57	0.15	3.4×10^{-5}	0.24	0.67	9.8×10^{-6}
Bacteria	*Marinomonas sp. MWYL1*	0.33	0.20	1.3×10^{-1}	0.23	0.27	6.5×10^{-1}
Bacteria	*Mesoplasma florum*	0.42	-0.12	2.7×10^{-1}	-0.15	0.11	6.0×10^{-1}
Bacteria	*Mesorhizobium loti*	0.54	0.06	3.8×10^{-9}	0.05	0.62	3.1×10^{-11}
Bacteria	*Methylacidiphilum infernorum*	0.19	-0.25	3.0×10^{-3}	0.18	-0.09	6.1×10^{-2}
Bacteria	*Methylibium petroleiphilum*	0.61	0.39	4.3×10^{-2}	0.25	0.83	5.3×10^{-7}
Bacteria	*Methylobacillus flagellatus*	0.65	0.14	2.8×10^{-5}	0.26	0.63	3.7×10^{-3}
Bacteria	*Methylobacterium chloromethanicum*	0.56	0.21	3.9×10^{-5}	0.11	0.67	8.7×10^{-10}
Bacteria	*Methylocella silvestris*	0.51	0.18	9.0×10^{-4}	0.16	0.63	1.8×10^{-5}
Bacteria	*Methylococcus capsulatus*	0.36	0.31	6.8×10^{-1}	0.10	0.54	4.8×10^{-4}
Bacteria	*Micrococcus luteus*	0.70	0.19	6.9×10^{-4}	-0.01	0.42	2.2×10^{-3}
Bacteria	*Microcystis aeruginosa*	0.28	0.12	5.0×10^{-2}	0.18	0.08	2.0×10^{-1}
Bacteria	*Moorella thermoacetica*	0.29	0.14	2.1×10^{-1}	0.22	0.32	4.1×10^{-1}
Bacteria	*Mycobacterium avium*	0.33	0.07	6.8×10^{-3}	-0.01	0.53	5.2×10^{-9}
Bacteria	*Mycoplasma agalactiae*	0.04	-0.21	5.8×10^{-1}	0.16	-0.08	5.1×10^{-1}
Bacteria	*Myxococcus xanthus*	0.56	0.34	6.3×10^{-3}	0.16	0.56	1.0×10^{-6}
Bacteria	*Natranaerobius thermophilus*	-0.07	0.13	1.2×10^{-1}	0.05	0.01	7.8×10^{-1}
Bacteria	*Nautilia profundicola*	0.19	0.14	7.2×10^{-1}	0.16	0.04	3.6×10^{-1}
Bacteria	*Neisseria gonorrhoeae*	0.46	0.05	4.3×10^{-3}	-0.20	0.50	3.5×10^{-5}
Bacteria	*Neorickettsia sennetsu*	-0.14	0.19	1.3×10^{-1}	-0.09	0.19	2.2×10^{-1}
Bacteria	*Nitratiruptor sp. SB155-2*	0.41	-0.07	4.8×10^{-4}	0.22	-0.09	3.0×10^{-2}
Bacteria	*Nitrobacter hamburgensis*	0.52	0.24	4.6×10^{-3}	0.06	0.79	1.6×10^{-12}
Bacteria	*Nitrosococcus oceani*	0.35	0.17	1.3×10^{-1}	0.06	0.23	1.9×10^{-1}
Bacteria	*Nitrosomonas europaea*	0.40	0.06	6.2×10^{-3}	0.21	0.36	2.5×10^{-1}
Bacteria	*Nitrosospira multiformis*	0.34	0.12	6.0×10^{-2}	0.32	0.35	8.1×10^{-1}
Bacteria	*Nocardia farcinica*	0.35	0.12	1.2×10^{-2}	0.11	0.62	1.0×10^{-8}
Bacteria	*Nocardioides sp. JS614*	0.39	0.21	7.1×10^{-2}	0.19	0.50	1.2×10^{-3}
Bacteria	*Nostoc punctiforme*	0.13	0.09	6.0×10^{-1}	0.19	0.12	3.7×10^{-1}
Bacteria	*Novosphingobium aromaticivorans*	0.42	0.34	4.5×10^{-1}	0.34	0.63	1.7×10^{-2}
Bacteria	*Oceanobacillus iheyensis*	0.01	0.06	7.0×10^{-1}	-0.03	0.13	1.5×10^{-1}
Bacteria	*Ochrobactrum anthropi*	0.53	0.16	4.0×10^{-5}	0.21	0.53	4.1×10^{-4}
Bacteria	*Oenococcus oeni*	0.29	0.12	2.1×10^{-1}	0.05	0.07	9.0×10^{-1}
Bacteria	*Oligotropha carboxidovorans*	0.59	0.14	1.5×10^{-5}	0.18	0.66	2.2×10^{-5}
Bacteria	*Onion yellows*	0.11	-0.03	5.8×10^{-1}	0.07	0.16	6.7×10^{-1}
Bacteria	*Opitutus terrae*	0.76	0.40	7.5×10^{-5}	0.32	0.96	2.2×10^{-11}
Bacteria	*Orientia tsutsugamushi*	-1.45	-0.26	4.2×10^{-5}	-0.18	-0.30	5.7×10^{-1}
Bacteria	*Parabacteroides distasonis*	0.75	0.17	4.8×10^{-9}	0.45	0.46	9.0×10^{-1}
Bacteria	*Paracoccus denitrificans*	0.51	0.20	1.7×10^{-3}	0.08	0.81	2.4×10^{-14}
Bacteria	*Parvibaculum lavamentivorans*	0.56	0.27	6.0×10^{-3}	0.18	0.70	9.1×10^{-7}
Bacteria	*Pasteurella multocida*	0.38	0.06	3.6×10^{-2}	-0.03	0.21	1.2×10^{-1}
Bacteria	*Pectobacterium atrosepticum*	0.40	0.03	6.9×10^{-5}	0.19	0.37	4.6×10^{-2}
Bacteria	*Pediococcus pentosaceus*	0.34	0.02	2.1×10^{-2}	0.32	0.27	6.6×10^{-1}
Bacteria	*Pelobacter carbinolicus*	0.52	-0.12	1.5×10^{-8}	0.17	0.37	7.4×10^{-2}
Bacteria	*Pelodictyon luteolum*	0.44	0.12	1.2×10^{-2}	0.26	0.53	4.9×10^{-2}
Bacteria	*Pelotomaculum thermopropionicum*	0.39	0.06	3.0×10^{-3}	0.03	0.33	7.8×10^{-3}
Bacteria	*Persephonella marina*	0.10	0.14	7.9×10^{-1}	-0.05	0.20	7.3×10^{-2}
Bacteria	*Petrotoga mobilis*	0.29	-0.04	2.8×10^{-2}	0.07	-0.05	4.6×10^{-1}
Bacteria	*Phenylobacterium zucineum*	0.66	0.45	6.2×10^{-2}	0.27	0.76	4.1×10^{-5}
Bacteria	*Photobacterium profundum*	0.19	-0.05	8.0×10^{-3}	0.17	0.08	3.0×10^{-1}
Bacteria	*Photorhabdus luminescens*	0.34	0.03	1.7×10^{-3}	0.12	0.07	6.2×10^{-1}
Bacteria	*Polaromonas naphthalenivorans*	0.97	0.10	1.1×10^{-17}	0.26	1.00	3.7×10^{-11}
Bacteria	*Polynucleobacter necessarius*	0.26	0.21	6.9×10^{-1}	0.18	0.20	8.7×10^{-1}
Bacteria	*Porphyromonas gingivalis*	0.54	0.12	1.2×10^{-3}	0.32	0.22	4.5×10^{-1}
Bacteria	*Prochlorococcus marinus*	-0.09	-0.08	9.7×10^{-1}	0.11	0.08	7.9×10^{-1}
Bacteria	*Propionibacterium acnes*	0.11	0.09	8.7×10^{-1}	0.19	0.08	4.5×10^{-1}
Bacteria	*Prosthecochloris aestuarii*	0.53	0.29	4.5×10^{-2}	0.34	0.12	9.1×10^{-2}
Bacteria	*Proteus mirabilis*	0.25	-0.07	4.9×10^{-3}	0.19	0.00	1.0×10^{-1}
Bacteria	*Pseudoalteromonas atlantica*	0.26	0.13	1.6×10^{-1}	0.14	0.26	2.1×10^{-1}
Bacteria	*Pseudomonas aeruginosa*	0.60	0.26	2.0×10^{-5}	0.19	0.77	3.3×10^{-11}
Bacteria	*Psychrobacter arcticus*	0.40	0.31	4.3×10^{-1}	0.29	0.25	7.6×10^{-1}
Bacteria	*Psychromonas ingrahamii*	0.40	0.24	7.7×10^{-2}	0.27	0.25	8.7×10^{-1}
Bacteria	*Ralstonia solanacearum*	0.98	0.27	1.4×10^{-10}	0.31	0.98	6.3×10^{-9}
Bacteria	*Renibacterium salmoninarum*	0.20	0.04	1.7×10^{-1}	0.04	0.09	6.9×10^{-1}
Bacteria	*Rhizobium etli*	0.51	0.23	2.7×10^{-3}	0.25	0.64	6.5×10^{-5}
Bacteria	*Rhodobacter sphaeroides*	0.65	0.47	1.2×10^{-1}	0.33	0.61	2.7×10^{-2}
Bacteria	*Rhodococcus erythropolis*	0.37	0.12	3.3×10^{-3}	0.07	0.29	1.4×10^{-2}
Bacteria	*Rhodoferax ferrireducens*	0.79	0.41	4.6×10^{-5}	0.37	0.82	2.6×10^{-6}
Bacteria	*Rhodopirellula baltica*	-0.04	-0.06	8.1×10^{-1}	-0.09	0.22	2.1×10^{-4}
Bacteria	*Rhodopseudomonas palustris*	0.67	0.06	3.2×10^{-10}	0.11	0.79	1.1×10^{-11}
Bacteria	*Rhodospirillum centenum*	0.66	0.25	3.2×10^{-4}	0.18	0.64	3.8×10^{-5}
Bacteria	*Rickettsia akari*	0.22	0.08	4.0×10^{-1}	0.11	0.01	5.4×10^{-1}
Bacteria	*Roseiflexus castenholzii*	0.40	0.19	2.6×10^{-2}	0.17	0.42	5.1×10^{-3}

Continued on next page

		GC			*ENC*		
Type	Species	Highest 5%	Lowest 5%	P-value	Highest 5%	Lowest 5%	P-value
Bacteria	*Roseobacter denitrificans*	0.43	0.43	9.9×10^{-1}	0.43	0.53	3.1×10^{-1}
Bacteria	*Rubrobacter xylanophilus*	0.24	0.14	4.0×10^{-1}	0.17	0.41	4.8×10^{-2}
Bacteria	*Ruegeria pomeroyi*	0.55	0.38	1.2×10^{-1}	0.34	0.75	4.1×10^{-4}
Bacteria	*Saccharophagus degradans*	0.37	0.25	1.9×10^{-1}	0.20	0.06	1.4×10^{-1}
Bacteria	*Saccharopolyspora erythraea*	0.33	0.08	1.2×10^{-3}	0.11	0.47	5.1×10^{-6}
Bacteria	*Salinibacter ruber*	0.67	0.42	4.3×10^{-2}	0.28	0.87	1.1×10^{-6}
Bacteria	*Salinispora arenicola*	0.38	0.24	1.4×10^{-1}	0.09	0.36	2.6×10^{-3}
Bacteria	*Salmonella enterica*	0.45	0.02	5.4×10^{-6}	0.09	0.39	3.0×10^{-3}
Bacteria	*Serratia proteamaculans*	0.58	0.08	4.9×10^{-8}	0.26	0.50	5.1×10^{-3}
Bacteria	*Shewanella amazonensis*	0.45	0.22	1.8×10^{-2}	0.18	0.39	4.1×10^{-2}
Bacteria	*Shigella boydii*	0.64	0.18	3.4×10^{-6}	0.15	0.40	1.2×10^{-2}
Bacteria	*Sinorhizobium medicae*	0.50	0.17	1.4×10^{-3}	0.21	0.61	2.3×10^{-4}
Bacteria	*Sodalis glossinidius*	0.55	0.20	9.3×10^{-3}	0.24	0.43	1.4×10^{-1}
Bacteria	*Solibacter usitatus*	0.35	0.24	1.1×10^{-1}	0.18	0.33	4.0×10^{-2}
Bacteria	*Sorangium cellulosum*	0.49	0.35	3.4×10^{-2}	0.20	0.62	8.3×10^{-10}
Bacteria	*Sphingomonas wittichii*	0.55	0.32	1.7×10^{-2}	0.27	0.66	3.2×10^{-5}
Bacteria	*Sphingopyxis alaskensis*	0.47	0.32	2.0×10^{-1}	0.27	0.56	1.3×10^{-2}
Bacteria	*Staphylococcus aureus*	0.06	-0.01	5.9×10^{-1}	-0.05	0.02	6.4×10^{-1}
Bacteria	*Stenotrophomonas maltophilia*	0.66	0.20	7.3×10^{-6}	0.11	0.96	6.9×10^{-14}
Bacteria	*Streptococcus agalactiae*	0.07	0.14	5.5×10^{-1}	0.20	-0.07	5.6×10^{-2}
Bacteria	*Streptomyces avermitilis*	0.54	0.19	8.9×10^{-6}	0.22	0.48	1.5×10^{-3}
Bacteria	*Sulfurihydrogenibium azorense*	0.05	-0.06	4.6×10^{-1}	-0.30	-0.05	1.6×10^{-1}
Bacteria	*Sulfurimonas denitrificans*	0.27	0.13	3.3×10^{-1}	0.09	0.14	6.5×10^{-1}
Bacteria	*Sulfurovum sp. NBC37-1*	0.43	0.20	3.7×10^{-2}	0.40	0.31	4.0×10^{-1}
Bacteria	*Symbiobacterium thermophilum*	0.44	0.05	9.2×10^{-4}	-0.01	0.39	1.6×10^{-3}
Bacteria	*Synechococcus elongatus*	0.38	0.04	6.0×10^{-3}	0.17	0.44	2.2×10^{-2}
Bacteria	*Synechocystis sp. PCC 6803*	0.43	-0.09	9.3×10^{-7}	0.22	0.35	2.4×10^{-1}
Bacteria	*Syntrophobacter fumaroxidans*	0.38	0.05	1.3×10^{-3}	0.19	0.47	6.0×10^{-3}
Bacteria	*Syntrophomonas wolfei*	0.20	-0.02	7.7×10^{-2}	0.16	0.23	5.8×10^{-1}
Bacteria	*Syntrophus aciditrophicus*	0.44	0.04	7.2×10^{-5}	0.07	0.33	2.2×10^{-2}
Bacteria	*Thauera sp. MZ1T*	0.72	0.35	5.4×10^{-4}	0.39	0.86	1.9×10^{-5}
Bacteria	*Thermoanaerobacter pseudethanolicus*	0.03	-0.09	3.7×10^{-1}	0.09	0.00	5.2×10^{-1}
Bacteria	*Thermobifida fusca*	0.44	0.29	1.9×10^{-1}	0.06	0.18	3.2×10^{-1}
Bacteria	*Thermodesulfovibrio yellowstonii*	0.21	0.02	1.3×10^{-1}	-0.05	-0.02	8.3×10^{-1}
Bacteria	*Thermomicrobium roseum*	0.34	0.10	8.8×10^{-2}	0.15	0.43	5.3×10^{-2}
Bacteria	*Thermosipho africanus*	-0.12	-0.10	9.1×10^{-1}	-0.01	-0.08	6.7×10^{-1}
Bacteria	*Thermosynechococcus elongatus*	0.23	0.16	5.6×10^{-1}	0.12	0.19	6.0×10^{-1}
Bacteria	*Thermotoga lettingae*	0.12	-0.10	1.1×10^{-1}	-0.07	-0.07	9.9×10^{-1}
Bacteria	*Thermus thermophilus*	0.11	0.09	8.6×10^{-1}	0.25	0.22	8.9×10^{-1}
Bacteria	*Thioalkalivibrio sp. HL-EbGR7*	0.73	0.13	3.3×10^{-7}	0.08	0.90	1.2×10^{-9}
Bacteria	*Thiobacillus denitrificans*	0.42	0.26	1.7×10^{-1}	0.20	0.87	1.0×10^{-6}
Bacteria	*Thiomicrospira crunogena*	0.36	0.21	2.2×10^{-1}	0.35	0.30	7.1×10^{-1}
Bacteria	*Tolumonas auensis*	0.53	0.21	1.5×10^{-3}	0.17	0.39	5.9×10^{-2}
Bacteria	*Treponema denticola*	0.37	-0.01	1.9×10^{-3}	0.34	0.01	8.6×10^{-3}
Bacteria	*Trichodesmium erythraeum*	0.17	-0.03	3.8×10^{-2}	0.10	0.13	7.9×10^{-1}
Bacteria	*Tropheryma whipplei*	-0.39	-0.23	5.0×10^{-1}	0.00	-0.10	6.6×10^{-1}
Bacteria	*Ureaplasma parvum*	-0.17	-0.50	6.2×10^{-1}	-0.32	0.29	2.5×10^{-1}
Bacteria	*Variovorax paradoxus*	0.82	0.46	8.3×10^{-6}	0.41	1.06	5.4×10^{-15}
Bacteria	*Verminephrobacter eiseniae*	0.53	0.29	1.3×10^{-2}	0.25	0.77	1.4×10^{-7}
Bacteria	*Vibrio cholerae*	0.41	0.10	2.1×10^{-3}	0.11	0.05	6.1×10^{-1}
Bacteria	*Wigglesworthia glossinidia*	-0.45	-0.22	4.3×10^{-1}	-0.15	-0.11	8.6×10^{-1}
Bacteria	*Wolbachia endosymbiont*	0.21	0.04	3.1×10^{-1}	0.06	0.06	9.8×10^{-1}
Bacteria	*Wolinella succinogenes*	0.21	-0.19	9.4×10^{-3}	0.23	0.40	1.9×10^{-1}
Bacteria	*Xanthobacter autotrophicus*	0.62	0.24	4.6×10^{-5}	0.16	0.68	4.5×10^{-7}
Bacteria	*Xanthomonas axonopodis*	0.59	0.17	2.8×10^{-5}	-0.03	0.80	1.5×10^{-14}
Bacteria	*Xylella fastidiosa*	0.45	-0.02	1.2×10^{-4}	0.05	0.21	2.5×10^{-1}
Bacteria	*Yersinia enterocolitica*	0.43	0.08	2.0×10^{-4}	0.13	0.24	2.4×10^{-1}
Bacteria	*Zymomonas mobilis*	0.08	-0.43	2.9×10^{-3}	0.21	-0.05	8.8×10^{-2}
Archaea	*Aeropyrum pernix*	0.32	-0.08	1.3×10^{-2}	-0.13	0.21	3.0×10^{-2}
Archaea	*Caldivirga maquilingensis*	-0.02	-0.03	9.1×10^{-1}	-0.10	-0.19	5.4×10^{-1}
Archaea	*Desulfurococcus kamchatkensis*	0.07	0.12	7.3×10^{-1}	0.22	-0.05	9.5×10^{-2}
Archaea	*Haloarcula marismortui*	0.30	0.13	1.1×10^{-1}	0.20	0.62	1.1×10^{-4}
Archaea	*Halobacterium salinarum*	0.24	0.17	5.8×10^{-1}	0.21	0.40	1.8×10^{-1}
Archaea	*Haloquadratum walsbyi*	0.02	-0.06	5.3×10^{-1}	0.12	0.10	8.9×10^{-1}
Archaea	*Halorubrum lacusprofundi*	0.38	0.28	3.8×10^{-1}	0.24	0.46	5.9×10^{-2}
Archaea	*Hyperthermus butylicus*	0.06	-0.14	2.5×10^{-1}	-0.12	-0.05	6.7×10^{-1}
Archaea	*Ignicoccus hospitalis*	-0.02	0.06	6.0×10^{-1}	0.24	0.10	4.1×10^{-1}
Archaea	*Metallosphaera sedula*	0.14	-0.21	7.7×10^{-3}	0.14	0.06	5.5×10^{-1}
Archaea	*Methanobrevibacter smithii*	0.22	0.18	7.4×10^{-1}	0.07	0.14	6.5×10^{-1}
Archaea	*Methanocaldococcus jannaschii*	0.06	-0.01	6.5×10^{-1}	0.25	-0.12	3.6×10^{-2}
Archaea	*Methanococcoides burtonii*	0.52	-0.02	5.8×10^{-6}	0.05	0.25	1.4×10^{-1}
Archaea	*Methanococcus aeolicus*	-0.07	0.02	6.1×10^{-1}	-0.02	0.04	7.2×10^{-1}
Archaea	*Methanocorpusculum labreanum*	0.45	0.00	7.3×10^{-3}	0.41	0.53	4.1×10^{-1}

Continued on next page

		GC			*ENC*		
Type	Species	Highest 5%	Lowest 5%	P-value	Highest 5%	Lowest 5%	P-value
Archaea	*Methanoculleus marisnigri*	0.46	0.03	3.3×10^{-3}	0.00	0.38	1.2×10^{-2}
Archaea	*Methanopyrus kandleri*	0.01	-0.19	1.6×10^{-1}	0.02	-0.03	7.6×10^{-1}
Archaea	*Methanosaeta thermophila*	0.55	-0.17	8.8×10^{-5}	-0.27	0.09	5.6×10^{-2}
Archaea	*Methanosarcina acetivorans*	0.36	-0.15	2.0×10^{-7}	0.06	0.01	5.9×10^{-1}
Archaea	*Methanosphaera stadtmanae*	0.05	0.12	6.5×10^{-1}	0.16	0.09	6.5×10^{-1}
Archaea	*Methanosphaerula palustris*	0.44	0.24	9.2×10^{-2}	0.20	0.49	4.5×10^{-2}
Archaea	*Methanospirillum hungatei*	0.18	-0.10	9.1×10^{-3}	0.15	0.14	9.3×10^{-1}
Archaea	*Methanothermobacter thermautotrophicus*	0.23	-0.03	1.0×10^{-1}	-0.01	0.18	2.8×10^{-1}
Archaea	*Nanoarchaeum equitans*	-0.19	-0.11	7.8×10^{-1}	-0.58	-0.15	1.5×10^{-1}
Archaea	*Natronomonas pharaonis*	0.20	0.08	3.3×10^{-1}	0.19	0.31	3.4×10^{-1}
Archaea	*Nitrosopumilus maritimus*	-0.14	-0.11	8.1×10^{-1}	-0.13	-0.02	4.9×10^{-1}
Archaea	*Picrophilus torridus*	0.21	-0.19	1.8×10^{-2}	-0.06	-0.02	8.0×10^{-1}
Archaea	*Pyrobaculum aerophilum*	0.21	-0.12	7.9×10^{-3}	-0.03	0.08	4.1×10^{-1}
Archaea	*Pyrococcus abyssi*	-0.02	0.02	8.1×10^{-1}	0.10	0.18	6.1×10^{-1}
Archaea	*Staphylothermus marinus*	-0.05	-0.03	8.6×10^{-1}	-0.06	-0.10	8.2×10^{-1}
Archaea	*Sulfolobus acidocaldarius*	0.30	-0.03	4.5×10^{-3}	0.03	-0.06	5.0×10^{-1}
Archaea	*Thermococcus gammatolerans*	0.17	-0.03	1.5×10^{-1}	0.01	0.28	5.8×10^{-2}
Archaea	*Thermofilum pendens*	0.40	0.15	7.7×10^{-2}	0.19	0.37	1.9×10^{-1}
Archaea	*Thermoplasma acidophilum*	0.28	0.09	2.3×10^{-1}	-0.10	0.37	1.1×10^{-2}
Archaea	*Thermoproteus neutrophilus*	0.24	-0.10	3.9×10^{-2}	-0.17	0.08	1.3×10^{-1}
Fungi	*Aspergillus fumigatus*	0.59	0.00	1.8×10^{-21}	0.03	0.38	2.1×10^{-8}
Fungi	*Candida dubliniensis*	0.40	-0.11	7.5×10^{-10}	0.11	0.11	9.9×10^{-1}
Fungi	*Cryptococcus neoformans*	0.39	0.12	5.6×10^{-4}	0.15	0.45	1.2×10^{-4}
Fungi	*Debaryomyces hansenii*	0.38	-0.06	2.4×10^{-7}	0.19	0.42	4.1×10^{-3}
Fungi	*Encephalitozoon cuniculi*	0.09	0.09	1	0.14	0.35	1.4×10^{-1}
Fungi	*Eremothecium gossypii*	0.80	-0.05	7.4×10^{-18}	0.12	0.87	8.5×10^{-15}
Fungi	*Kluyveromyces lactis*	0.33	0.00	4.0×10^{-4}	0.04	0.49	3.5×10^{-6}
Fungi	*Pichia stipitis*	0.34	-0.04	9.8×10^{-6}	-0.01	0.57	3.7×10^{-10}
Fungi	*Saccharomyces cerevisiae*	0.50	0.02	1.1×10^{-10}	0.13	0.46	8.2×10^{-5}
Fungi	*Schizosaccharomyces pombe*	0.26	0.00	6.4×10^{-3}	-0.02	0.19	2.1×10^{-2}
Fungi	*Yarrowia lipolytica*	0.52	0.06	4.1×10^{-8}	0.01	0.57	3.5×10^{-10}
Plant	*Arabidopsis thaliana*	0.17	-0.04	5.0×10^{-8}	0.03	0.05	6.1×10^{-1}
Plant	*Oryza sativa*	0.34	-0.24	1.1×10^{-31}	-0.57	0.46	4.8×10^{-81}
Insect	*Anopheles gambiae*	0.47	0.04	4.8×10^{-11}	0.09	0.64	2.2×10^{-15}
Insect	*Drosophila melanogaster*	0.34	-0.08	1.2×10^{-13}	0.07	0.55	7.4×10^{-17}
Fish	*Danio rerio*	0.24	0.07	2.9×10^{-4}	-0.02	0.23	6.6×10^{-7}
Fish	*Gasterosteus aculeatus*	0.21	0.02	1.3×10^{-3}	0.11	0.35	3.6×10^{-5}
Fish	*Oryzias latipes*	0.14	0.03	8.2×10^{-2}	-0.03	0.23	1.9×10^{-5}
Fish	*Takifugu rubripes*	0.26	-0.01	2.3×10^{-4}	-0.01	0.30	5.1×10^{-6}
Bird	*Gallus gallus*	0.07	-0.14	2.2×10^{-3}	-0.51	0.02	3.5×10^{-12}
Bird	*Taeniopygia guttata*	0.03	-0.38	6.5×10^{-6}	-0.39	0.01	2.3×10^{-5}
Mammal	*Bos taurus*	0.11	-0.19	3.8×10^{-7}	-0.46	0.16	4.8×10^{-24}
Mammal	*Canis familiaris*	0.12	-0.31	7.9×10^{-11}	-0.46	0.20	4.2×10^{-21}
Mammal	*Equus caballus*	0.13	-0.22	1.5×10^{-9}	-0.37	0.11	4.5×10^{-14}
Mammal	*Homo sapiens*	0.12	-0.30	2.2×10^{-18}	-0.39	0.12	4.4×10^{-25}
Mammal	*Loxodonta africana*	0.13	-0.18	4.6×10^{-3}	-0.20	0.17	2.5×10^{-3}
Mammal	*Mus musculus*	0.07	-0.26	3.1×10^{-13}	-0.32	0.08	5.1×10^{-19}
Mammal	*Pteropus vampyrus*	0.14	-0.28	7.2×10^{-8}	-0.37	0.09	2.4×10^{-8}
Mammal	*Tursiops truncatus*	0.11	-0.26	7.3×10^{-6}	-0.44	0.20	8.4×10^{-14}

Note.—The P-values were obtained by t-test.
